# Sex Differences in Hemostatic Factors in Patients With Ischemic Stroke and the Relation With Migraine—A Systematic Review

**DOI:** 10.3389/fncel.2021.711604

**Published:** 2021-11-11

**Authors:** Nelleke van der Weerd, Hine J. A. van Os, Mariam Ali, Jan W. Schoones, Arn M. J. M. van den Maagdenberg, Nyika D. Kruyt, Bob Siegerink, Marieke J. H. Wermer

**Affiliations:** ^1^Department of Neurology, Leiden University Medical Centre, Leiden, Netherlands; ^2^Department of Human Genetics, Leiden University Medical Centre, Leiden, Netherlands; ^3^Department of Neurology, Amsterdam University Medical Centre, Vrije Universiteit Amsterdam, Amsterdam, Netherlands; ^4^Directorate of Research Policy, Leiden University Medical Centre, Leiden, Netherlands; ^5^Department of Neurology, University Neurovascular Centre, The Hague, Netherlands; ^6^Department of Clinical Epidemiology, Leiden University Medical Centre, Leiden, Netherlands

**Keywords:** male, female, risk factor, migraine, coagulation, plasma, serum

## Abstract

**Background:** Women are more affected by stroke than men. This might, in part, be explained by sex differences in stroke pathophysiology. The hemostasis system is influenced by sex hormones and associated with female risk factors for stroke, such as migraine.

**Aim:** To systematically review possible sex differences in hemostatic related factors in patients with ischemic stroke in general, and the influence of migraine on these factors in women with ischemic stroke.

**Results:** We included 24 studies with data on sex differences of hemostatic factors in 7247 patients with ischemic stroke (mean age 57–72 years, 27–57% women) and 25 hemostatic related factors. Levels of several factors were higher in women compared with men; FVII:C (116% ± 30% vs. 104% ± 30%), FXI (0.14 UI/mL higher in women), PAI-1 (125.35 ± 49.37 vs. 96.67 ± 38.90 ng/mL), D-dimer (1.25 ± 0.31 vs. 0.95 ± 0.24 μg/mL), and aPS (18.7% vs. 12.0% positive). In contrast, protein-S (86.2% ± 23.0% vs. 104.7% ± 19.8% antigen) and P-selectin (48.9 ± 14.4 vs. 79.1 ± 66.7 pg/mL) were higher in men. Most factors were investigated in single studies, at different time points after stroke, and in different stroke subtypes. Only one small study reported data on migraine and hemostatic factors in women with ischemic stroke. No differences in fibrinogen, D-dimer, t-PA, and PAI-1 levels were found between women with and without migraine.

**Conclusion:** Our systematic review suggests that sex differences exist in the activation of the hemostatic system in ischemic stroke. Women seem to lean more toward increased levels of procoagulant factors whereas men exhibit increased levels of coagulation inhibitors. To obtain better insight in sex-related differences in hemostatic factors, additional studies are needed to confirm these findings with special attention for different stroke phases, stroke subtypes, and not in the least women specific risk factors, such as migraine.

## Introduction

Stroke is the third most disabling disease worldwide. Women are particularly affected because of a higher stroke incidence and a worse outcome compared with men (Girijala et al., 2017). Evidence accumulates that these differences might, in part, be explained by sex-specific pathophysiological mechanisms underlying stroke (Demel et al., 2018).

Multiple mechanisms and pathways are involved in the pathophysiology of stroke. More precise atherosclerosis, oxidative stress, endothelial and mitochondrial dysfunction, inflammation, complement activation, and hemostatic factors are associated with ischemic stroke (Periayah et al., 2017; Liu et al., 2018; Ma et al., 2019; Shao et al., 2020).

In addition to traditional vascular risk factors, women have specific stroke risk factors, such as the presence of reproductive disorders, the use of oral contraceptives, and the co-occurrence of migraine with aura. Especially the comorbidity between ischemic stroke and cardiovascular disease with migraine is recognized already for decades (Kurth et al., 2012; Sacco et al., 2017; Adelborg et al., 2018; Demel et al., 2018). Both men and women with migraine with aura have an approximately twofold increased risk of ischemic stroke (Adelborg et al., 2018). However, the point estimates of the association between migraine and stroke seem to be higher in women and the risk is further increased to sevenfold in women who use oral contraceptives (Etminan et al., 2005; Schürks et al., 2009) and even ninefold when they are also smoking (Schürks et al., 2009). A vascular mechanistic link between migraine and ischemic stroke has been proposed, linking the increased risk and underlying disease mechanisms (Kurth et al., 2012). A possible link between hemostatic factors for migraine and ischemic stroke has previously been shown in genetic studies. For instance, prothrombotic genotypes factor V Leiden and prothrombin G20210A were more frequently present in young patients with ischemic stroke and a history of migraine with aura compared with young patients with migraine without aura or no history of migraine (Pezzini et al., 2011). Furthermore, in a mendelian randomization study, genetically determined increased levels of hemostatic factors FVIII, von Willebrand factor (vWF), phosphorylated fibrinopeptide A, and a decrease of fibrinogen seemed causally related to susceptibility for migraine, especially migraine with aura (Guo Y. et al., 2021).

Sex differences in hemostatic factors, alone or in combination with female risk factors, could increase the risk of ischemic stroke in women (Siegerink et al., 2010; Sacco et al., 2017). The hemostatic system can be divided into primary hemostasis (platelet activation and aggregation), secondary hemostasis (coagulation cascade), and the fibrinolytic pathway. A systematic review suggested that some factors of the hemostatic system, for example vWF, FXI, prothrombin fragment 1 + 2 (F1.2), D-dimer, plasminogen activator inhibitor 1 (PAI-1) and anti-phosphatidyl serine antibodies (aPS), are related to poor clinical outcome after ischemic stroke. However, it is yet unclear whether these factors could be used as predictors for stroke outcome (Donkel et al., 2019). Also it is unknown whether they have additional value above other known prognostic factors in stroke (Deng et al., 2018; Li et al., 2019, 2020; Chang et al., 2021; Chen et al., 2021; Montellano et al., 2021). Female sex hormones can influence hemostatic factors causing the hemostasis system to function differently for men and women (Abou-Ismail et al., 2020). Sex differences in levels of hemostatic factors could therefore be a missing link in understanding sex differences in ischemic stroke risk and outcome.

Many hemostatic factors have previously been reviewed in relation to ischemic stroke, but sex differences have not yet been systemically evaluated (Periayah et al., 2017). We aimed to review possible sex differences of the hemostatic system, and the influence of migraine, by performing a systematic search on sex differences in plasma and/or serum levels of hemostatic related factors in ischemic stroke, and hemostatic related factors in women with ischemic stroke and migraine.

## Methods

This systematic review was performed conform the Preferred Reporting Items for Systematic Reviews and Meta-analysis (PRISMA) guidelines (Shamseer et al., 2015).

### Information Sources and Search Strategy

In cooperation with a trained librarian (JS), we composed two search strategies. The primary query consisted of the combination of three subjects: (1) stroke, (2) coagulation, platelet activation, hemostasis, primary hemostatic factors, and secondary hemostatic factors, and (3) sex differences. In the second query, we added to the subjects: (1) stroke, (2) coagulation platelet activation, hemostasis, primary hemostatic factors, and secondary hemostatic factors, the subject (3) migraine and changed sex differences to the subject (4) women, as migraine is a risk factor for stroke in women specifically. For the different concepts, all relevant keyword variations were used, not only keyword variations in the controlled vocabularies of the various databases, but the free text word variations of these concepts as well. The search strategy was optimized for all consulted databases, taking into account the differences of the various controlled vocabularies as well as the differences of database-specific technical variations (e.g., the use of quotation marks). Both searches were performed on January 2nd 2020 in the following databases: Pubmed, Embase (OVID-version), Emcare (OVID-version), Web of Science, and the Cochrane Library. Full details of the search strategies can be found in Supplementary Appendix 1.

### Eligibility Criteria

We included studies with ischemic stroke patients aged ≥18 years. For the first query on sex differences, single-sex studies were excluded. For both queries studies with specific criteria for patient selection (other than age ≥18 years) were excluded. Studies types included were: (1) study cohort, (2) case-control study, (3) cross-sectional study, (4) nested case-control study, and (5) clinical trials. Studies excluded were: (1) case reports, (2) case series, (3) reviews, and (4) meta-analyses. Included studies had to report on plasma and/or serum levels of hemostatic factors. When multiple publications on one cohort were found, they were all included when the studies investigated different factors. Otherwise the most recent one was included. Articles had to be written in English or Dutch.

### Study Selection

Two independent reviewers (NW and MA) first screened publications on title and abstract, followed by a second screening on full text. Disagreements were discussed in a consensus meeting with a third reviewer (HO). Titles and abstracts were screened based on: (1) population criteria, and (2) outcome (plasma levels in concentration and antiphospholipids as positive or negative). Population for query on sex differences screened for inclusion of both men and women, ≥18 years old, with ischemic stroke, query on stroke and migraine screened for inclusion of women, >18 years old, with ischemic stroke and migraine. Selection for full-text screening was based on: (1) results reported for men and women separately, and (2) ischemic stroke diagnosis confirmed by clinical and neuro-imaging (CT or MRI) assessments.

### Data Extraction

We collected the following baseline information from the selected papers: number of patients (per sex), age, risk factors (smoking, diabetes mellitus, hypertension, and BMI), ischemic stroke etiology, and timing of blood draw after stroke. In addition, concentrations of serum and/or plasma levels of hemostatic factors were collected. Factors were divided into primary hemostasis, secondary hemostasis, fibrinolytic pathway, and other factors. We defined the group “other factors” as a group of factors that influence hemostasis directly but are not hemostatic factors. Risk of bias was assessed using Grading of Recommendations Assessment, Development, and Evaluation (GRADE).

## Results

### Study Characteristics Sex Differences in Hemostatic Factors

**FIGURE 1:**
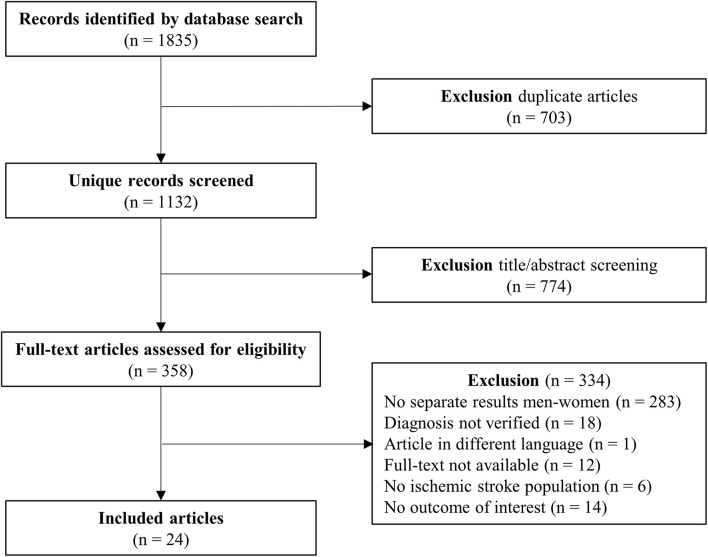
PRISMA flow chart study selection sex differences in ischemic stroke.

The first query on sex differences in ischemic stroke resulted in 1132 studies, of which 24 were included (Figure 1) with data on a total of 7217 patients. The number of patients per study ranged from 30 to 3342, with a median of 152. The proportion of women in all included studies ranged from 27 to 57%. Mean age ranged from 57 to 77 years across included studies. The characteristics of the included articles are summarized in Supplementary Table 1. Most often reported vascular risk factors in the articles were smoking, hypertension, diabetes mellitus, and BMI (Supplementary Table 2). Large differences in these risk factors were seen between the studies, except for BMI (between 24 and 26). Smoking ranging from 18 to 62%, hypertension ranging from 44 to 84%, and diabetes mellitus ranging from 13 to 60%.

A total of 25 different factors were reported: seven primary hemostatic factors, nine secondary hemostatic factors, four fibrinolytic pathway factors, and five other factors related to hemostasis. All primary and secondary hemostatic factors were investigated in only one study. Fibrinolytic pathway factors and other factors involved in hemostasis were investigated in multiple studies. A meta-analysis could not be performed due to the many single studies and large heterogeneity between the studies.

Risk of bias was assessed for each article (Supplementary Table 3). No risk of bias was found for selection of men and women as they were always selected from the same stroke population. For 23 of the 24 articles, study populations were included from a single center or region, therefore only partly representative for the whole ischemic stroke population. In most cases (20 of 24), the confidence in the assessment of outcome is high, with most studies specifically addressing the timing of blood sampling. However, in only 14 of 24 studies timing of blood sampling was similar for both men and women. Another concern was the assessment and adjustment for confounding, with most studies (19 of 24) adjusting for very few or no confounding factors at all.

### Sex Differences in Primary Hemostasis

Seven primary hemostatic related factors were reported in five articles, with a total of 1115 patients (Table 1). No sex differences were found for glutamate (Meng et al., 2014) and phospholipase A2 (PLA2) (Elkind et al., 2006) within 72h after stroke, thromboxane 2 (TXA2), prostaglandin I2 (PGI2), adenosine diphosphate (ADP) within 2 weeks after stroke (Lee et al., 1987), and vWF (Kain et al., 2001) 3 months after stroke. P-selectin plasma levels were lower in women than in men at day 1 (79.1 ± 66.7 vs. 48.9 ± 15.4 in pg/mL) but not on day 4 (113.6 ± 82.6 vs. 83.5 ± 46.4 in pg/mL) after stroke (Blum et al., 2012).

**TABLE 1:** Primary hemostatic factors.

	**Men [mean ± SD median (IQR)]**	**Women [mean ± SD median (IQR)]**	**Sex difference**	**Study**
TXA2 (pg/mL)*	<2w *n* = 31: 105.01 ± 92.56 *n* = 31: 94.90 ± 76.18 *n* = 20: 96.96 ± 57.39 *n* = 20: 92.18 ± 56.97	<2w *n* = 25: 81.43 ± 64.05 *n* = 20: 84.84 ± 82.49 *n* = 11: 94.54 ± 56.57 *n* = 13: 84.05 ± 44.18	NS	Lee et al., 1987
PGI2 (pg/mL)*	<2w *n* = 8: 42.25 ± 20.30 *n* = 7: 36.10 ± 10.77	<2w *n* = 8: 43.38 ± 17.85 *n* = 7: 37.87 ± 18.63	NS	Lee et al., 1987
ADP threshold concentration (μM)*	<2w *n* = 40: 4.20 ± 2.77 *n* = 44: 4.58 ± 3.05 *n* = 20: 4.53 ± 2.83 *n* = 23: 4.86 ± 3.32	<2w *n* = 28: 4.08 ± 2.47 *n* = 28: 4.47 ± 2.81 *n* = 13: 4.48 ± 2.17 *n* = 13: 4.15 ± 2.87	NS	Lee et al., 1987
**vWF (IU/mL)**	3m: 1.8 ± 0.8	3m: 1.9 ± 0.8	NS	Kain et al., 2001
**Glutamate (μM)**	<72h: 187 (128–267)		NS	Meng et al., 2014
**P-selectin (pg/mL)**	1d: 79.1 ± 66.7 4d: 113.6 ± 82.6	1d: 48.9 ± 15.4 4d: 83.5 ± 46.4	*p* = 0.02 *p* = 0.08	Blum et al., 2012
**PLA2 (ng/mL)**	<72h: 326.8 ± 112.9	<72h: 336.1 ± 132.7	NS	Elkind et al., 2006

* Patients were divided in four groups to compare effect of a drug. The numbers shown here are the baseline measurements before any drug was administered.

### Sex Differences in Secondary Hemostasis

Nine secondary hemostatic factors were reported in eight articles, with a total of 1031 patients (Table 2). No sex differences were found for antithrombin III (AT III) (Haapaniemi et al., 2002), thrombin-antithrombin (Haapaniemi et al., 2004), and protein C (Haapaniemi et al., 2002), at four time points, β-antithrombin within 2 days after stroke (de la Morena-Barrio et al., 2015), and FXIIa 3 months after stroke (Kain et al., 2001). Women had higher FVII:ag (114% ± 34% vs. 99% ± 31%) 3 months after stroke (Kain et al., 2001) and FXIc levels (1.13 ± 0.32; with difference of 0.14 UI/mL) 1 month after stroke (Santamaria et al., 2007) compared with men. Protein S antigen percentages were reported to be lower in women than men in the first 2 days (86.2% ± 23.0% vs. 104.7% ± 19.8%), 1 week (72.3% ± 34.2% vs. 87.8% ± 4.3%), 1 month (80.6% ± 32.8% vs. 85.5% ± 28.5%), and 3 months (73.5% ± 28.0% vs. 91.2% ± 31.1%) after stroke (Haapaniemi et al., 2002). Four studies (Carter et al., 1997; Kain et al., 2001; Skoloudik et al., 2010; Kisialiou et al., 2012) did not report sex differences for fibrinogen levels. One study found higher fibrinogen levels in women with cortical infarcts of the anterior circulation [4.90 g/L (95% CI 4.48–5.32)] than in women with lacunar infarcts (4.04 g/L [95% CI 3.73–4.37)] in the first 10 days after stroke, but not 3 months after stroke. This effect was not observed in men (Carter et al., 1997).

**TABLE 2:** Secondary hemostatic factors.

	**Men [mean ± SD median (IQR)]**	**Women [mean ± SD median (IQR)]**	**Sex difference**	**Study**
**FVII:C (%)** **FVII:ag (%)**	3m: 104 ± 30 3m: 99 ± 31	3m: 116 ± 30 3m: 114 ± 34	*p* = 0.04 *p* = 0.01	Kain et al., 2001
**FXIIa (ng/mL)**	3m: 2.2 ± 1.0	3m: 2.4 ± 1.1	NS	Kain et al., 2001
**FXI (UI/mL)**	1m: 1.13 ± 0.32 (0.14 higher in women than in men)		95%CI: 0.09–0.19	Santamaria et al., 2007
**AT III (% activity)**	<2d: 102.5 ± 12.4 1w: 106.1 ± 13.8 1m: 113.5 ± 15.7 3m: 113.3 ± 12.5	<2d: 103.6 ± 16.9 1w: 114.4 ± 16.6 1m: 112.8 ± 12.5 3m: 112.6 ± 13.2	NS	Haapaniemi et al., 2002
**β-antithrombin (% activity)**	<2d: 105.9 ± 17.4		NS	de la Morena-Barrio et al., 2015
**Thrombin-antithrombin (mg/L)**	<2d: 7.9 ± 9.1 1w: 6.6 ± 8.6 1m: 4.5 ± 3.3 3m: 6.0 ± 6.2		NS	Haapaniemi et al., 2004
**protein S (% antigen)**	<2d: 104.7 ± 19.8 1w: 87.8 ± 4.3 1m: 85.5 ± 28.5 3m: 91.2 ± 31.1	<2d: 86.2 ± 23.0 1w: 72.3 ± 34.2 1m: 80.6 ± 32.8 3m: 73.5 ± 28.0	*p* < 0.05 NS NS *p* < 0.01	Haapaniemi et al., 2002
**Protein C (% activity)**	<2d: 118.6 ± 28.4 1w: 108.3 ± 42.4 1m: 109.0 ± 35.7 3m: 109.0 ± 43.5	<2d: 116.0 ± 30.6 1w: 125.8 ± 44.1 1m: 119.8 ± 44.7 3m: 111.7 ± 40.6	NS	Haapaniemi et al., 2002
**Fibrinogen (g/L)**	<10d: 4.57 (4.37–4.78) 3m: 3.86 (3.63–4.12)	<10d: 4.40 (4.22–4.58) 3m: 3.82 (3.64–4.02)	↑ in women with TACI (*p* = 0.021)	Carter et al., 1997
**Fibrinogen (g/L)**	3m: 3.8 ± 1.3	3m: 3.7 ± 1.3	NS	Kain et al., 2001
**Fibrinogen (g/L)**	3h: 3.4 ± 0.8 6h: 3.1 ± 0.8 24h: 3.4 ± 0.8		NS	Skoloudik et al., 2010
**Fibrinogen (g/L)**	<24h: 3.9 ± 1.2	<24h: 4.0 ± 1.8	NS	Kisialiou et al., 2012

### Sex Differences in the Fibrinolytic Pathway

Four factors of the fibrinolytic pathway were reported in nine articles, with a total of 1343 patients (Table 3). No sex differences were found for tissue plasminogen activator (t-PA) plasma levels (Jeppesen et al., 1998; Mansfield et al., 1998; Kain et al., 2001; Haapaniemi et al., 2004; Saidi et al., 2007) (decreases thrombotic tendency) and F1.2 (Furie et al., 2004; Haapaniemi et al., 2004) (increases thrombotic tendency), while varying results were reported for D-dimer and PAI-1 (both increase thrombotic tendency). Two studies found no sex differences for D-dimer levels within 24h after stroke (Skoloudik et al., 2010) or 3 months after stroke (Haapaniemi et al., 2004). One study found higher levels in women than in men (1.25 ± 0.31 vs. 0.95 ± 0.24 in μg/mL), but did not report how long after stroke blood was drawn (Li et al., 2018). Four of five articles reported higher PAI-1 levels 3 months after stroke in women compared with men [15.7 (95% CI 13.7–18.1) vs. 11.4 (10.2–12.7) in U/mL] (Mansfield et al., 1998), (18.2 ± 13.5 vs. 13.3 ± 11.0 in U/mL) (Kain et al., 2001), (16.2 ± 2.1 vs. 11.1 ± 2.4 in U/mL) (Kain et al., 2002), and (125.35 ± 49.37 vs. 96.67 ± 38.90 in U/mL) (Saidi et al., 2007). One study found no sex differences in PAI-1 activity at four different time points (Haapaniemi et al., 2004).

**TABLE 3:** Fibrinolysis factors.

	**Men [mean ± SD median (IQR)]**	**Women [mean ± SD median (IQR)]**	**Sex difference**	**Study**
**D-dimer (μg/mL)**	<2d: 0.66 ± 0.56 1w: 1.61 ± 3.18 1m: 0.79 ± 0.89 3m: 0.49 ± 0.42		NS	Haapaniemi et al., 2004
**D-dimer (μg/mL)**	3h: 0.31 ± 0.40 6h: 0.86 ± 1.04 24h: 0.59 ± 1.00		NS	Skoloudik et al., 2010
**D-dimer (μg/mL)**	0.95 ± 0.24*	1.25 ± 0.31*	*p* < 0.001	Li et al., 2018
**PAI-1 (U/mL)**	3m: 11.4 (10.2–12.7)	3m: 15.7 (13.7–18.1)	*p* = 0.001	Mansfield et al., 1998
**PAI-1 activity U/mL)** **PAI-1:ag (U/mL)**	3m: 16.1 ± 11.7 3m: 13.3 ± 11.0	3m: 19.3 ± 10.8 3m: 18.2 ± 13.5	*p* = 0.04 *p* = 0.04	Kain et al., 2001
**PAI-1 activity (U/mL)** **PAI-1:ag (U/mL)**	3m: 14.1 ± 2.0 3m: 11.1 ± 2.4	3m: 19.2 ± 1.7 3m: 16.2 ± 2.1	*p* < 0.05 *p* < 0.05	Kain et al., 2002
**PAI-1 (AU/mL)**	<2d: 17.2 ± 7.8 1w:11.2 ± 9.2 1m: 14.4 ± 7.9 3m: 15.8 ± 8.4		NS	Haapaniemi et al., 2004
**PAI-1 (ng/mL)**	96.7 ± 38.9*	125.4 ± 49.4*	*p* < 0.05**	Saidi et al., 2007
**t-PA:ag (ng/mL)**	<2w: 9.4 (6.7–12.1) 6m: 10.3 (9.1–14.6)	<2d: 10.1 (7.4–12.6) 6m: 10.9 (7.3–15.3)	NS	Jeppesen et al., 1998
**t-PA:ag (ng/mL)**	3m: 11.5 (10.7–12.2)	3m: 11.7 (10.8–12.6)	NS	Mansfield et al., 1998
**t PA:ag (ng/mL)**	3m: 11.4 ± 1.6	2m: 12.0 ± 1.7	NS	Kain et al., 2001
**t-PA:ag (ng/mL)**	0.8 ± 0.4*	0.7 ± 0.3*	NS	Saidi et al., 2007
**t-PA:ag (ng/mL)**	<2d: 8.8 ± 4.2 1w: 8.4 ± 3.4 1m: 8.3 ± 3.2 3m: 8.7 ± 4.3		NS	Haapaniemi et al., 2004
**F1.2 (ng/mL)**	3–6m: 0.9 (0.3–2.4)	3–6m: 1.0 (0.4–2.8)	*p* = 0.02	Furie et al., 2004
**F1.2 (nmol/L)**	<2d: 1.7 ± 0.9 1w: 1.7 ± 1.1 1m: 1.4 ± 0.8 3m: 1.6 ± 1.4		NS	Haapaniemi et al., 2004

* Unknown in which phase blood was drawn.

**Increased in women with ischemic stroke compared to controls, no difference in men between ischemic stroke patients and controls.

### Sex Differences in Other Hemostasis Associated Factors

Five other factors, that directly influence hemostasis, were reported in six articles, with a total of 4143 patients (Table 4). No sex differences were reported for plasma levels of tissue inhibitor metalloproteinase 1 (TIMP-1) (Zhong et al., 2019) and insulin-like growth factor 1 (IGF-1) (Dong et al., 2014; Zhang et al., 2018) 1 day after stroke, matrix metalloproteinase 2 (MMP2) within 2 days after stroke (Montaner et al., 2001), and matrix metalloproteinase 9 (MMP9) 2 days (Montaner et al., 2001; Zhong et al., 2019) and 1 month after stroke (Abdelnaseer et al., 2017). One study found higher aPS titers (18.7% vs. 12.0% positive) in women compared with men 5 days after ischemic stroke, however, this did not reach statistical significance (Tuhrim et al., 1999).

**TABLE 4:** Other factors involved in hemostasis.

	**Men [mean ± SD median (IQR)]**	**Women [mean ± SD median (IQR)]**	**Sex difference**	**Study**
**MMP2 (ng/mL)**	0h: 752.5 ± 210.9 12h: 668.8 ± 254.6 24h: 599.0 ± 195.4 48h: 580.1 ± 188.0 mean: 644.2 ± 185.8		NS	Montaner et al., 2001
**MMP9 (ng/mL)**	0h: 147.1 ± 118.6 12h: 140.4 ± 120.7 24h: 172.6 ± 139.0 48h: 144.5 ± 127.6 mean: 149.6 ± 99.0		NS	Montaner et al., 2001
**MMP9 (ng/mL)**	<24h: 668.9 (415.0–1022.0)		NS	Zhong et al., 2019
**MMP9 (ng/mL)**	24h: 998.8 ± 154.7 1m: 800.3 ± 156.4		NS	Abdelnaseer et al., 2017
**TIMP-1 (ng/mL)**	<24h: 198.6 (163.7–241.2)		NS	Zhong et al., 2019
**IGF-1 (ng/mL)**	<24h: 129 (109–153)		NS	Dong et al., 2014
**IGF-1 (ng/mL)**	<24h: 112 (92–142)		NS	Zhang et al., 2018
**Antiphosphatidyl serine (% positive)**	<5d: 12.0	<5d: 18.7	NS	Tuhrim et al., 1999

### Studies on Hemostatic Factors in Women With Ischemic Stroke and Migraine

**FIGURE 2:**
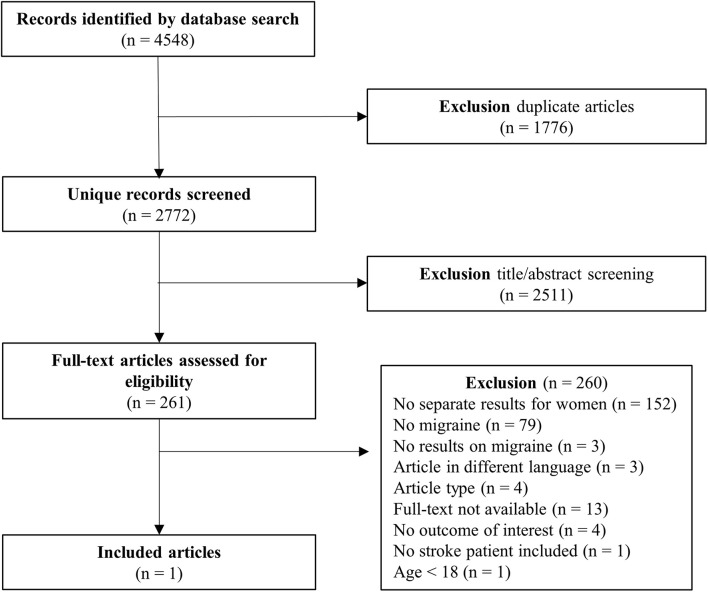
PRISMA flow chart study selection ischemic stroke and migraine in women.

The second query on ischemic stroke and migraine in women specifically resulted in 2772 studies, of which only one study was included (Figure 2). Sixteen women (mean age 37 years) with lacunar stroke were included, of whom 44% had migraine. Migraine patients were more often smokers (57% vs. 33%) and users of oral contraceptives (29% vs. 0%) (Salobir et al., 2002). Four hemostatic factors (fibrinogen, D-dimer, t-PA, and PAI-1) were investigated in the chronic phase after stroke. No clear differences were found between patients with and without migraine (Salobir et al., 2002).

## Discussion

We found sex differences for several hemostatic factors in patients with ischemic stroke. In women, levels of FVII (Kain et al., 2001), FXI (Santamaria et al., 2007), PAI-1 (Mansfield et al., 1998; Kain et al., 2001, 2002; Saidi et al., 2007), and D-dimer (Li et al., 2018) were higher compared with men. In contrast, levels of P-selectin (Blum et al., 2012) and protein S (Haapaniemi et al., 2002) were lower in women. We found no differences for hemostatic factors in women with ischemic stroke with or without migraine (Salobir et al., 2002) but only one study fulfilled our inclusion criteria.

Previous reviews on women with stroke have been performed but mainly addressed hemostatic factors in relation to oral contraceptives use (Lete et al., 2015) and menopause status (Sowers et al., 2005). Our review specifically focused on sex differences in ischemic stroke patients in general, and the influence of migraine on hemostatic factors in women with ischemic stroke. Overall, the sex differences in our review indicate that women with ischemic stroke lean more toward increased levels of procoagulant factors, whereas men lean more toward increased levels of coagulation inhibitors. FVII and FXI are involved in the activation of thrombin *via* the coagulation cascade of secondary hemostasis. Activation of thrombin leads to platelet activation and fibrin formation (Periayah et al., 2017). D-dimer is released when fibrin is crosslinked to form a clot. Increased D-dimer levels thus indicate activation of thrombin (Furie et al., 2004; Skoloudik et al., 2010), conform the higher levels of FVII and FXI. Further, FXI activation helps stabilize fibrin clots and makes the clot more resistant to fibrinolysis (Santamaria et al., 2007). The fibrinolysis pathway is inhibited by PAI-1, further increasing the thrombotic tendency (Saidi et al., 2007). Only two factors were higher in men of which protein S inhibits coagulation factors V and VIII (Haapaniemi et al., 2002) and P-selectin is released by activated platelets and endothelial cells (Blum et al., 2012). Fibrinogen is an acute phase reactant. We found no sex differences in levels of fibrinogen between men and women, but in women fibrinogen levels differed between cortical and lacunar location which could point to differences in underlying disease mechanisms. Interestingly, high levels of some of these factors (FXI, D-dimer, PAI-1, and aPS) have previously been associated with impaired clinical outcome after ischemic stroke (Donkel et al., 2019). Therefore, sex differences in the hemostatic system might contribute to both an increased risk as to a worse outcome after stroke in women compared with men (Girijala et al., 2017).

Another aspect of sex differences in ischemic stroke are the women specific risk factors, such as migraine, especially in combination with the use of oral contraceptives. We found only one study investigating the influence of migraine history on hemostatic factors in female stroke patients. This study included only 16 patients and found no influence on fibrinogen, D-dimer, t-PA, and PAI-1 (Salobir et al., 2002). Previous research has shown some evidence for a genetic predisposition for hypercoagulability in patients with migraine with aura (Guo Y. et al., 2021). Hypercoagulability and microemboli have been suggested to have a potential role in the association of migraine with aura with ischemic stroke. The increased risk of stroke in women with migraine using oral contraceptives further supports the hypothesis, since estrogen is associated with increase of FII, FVII, FX, prothrombin, and fibrinogen (Tietjen and Collins, 2018). Phosphorylated fibrinopeptide A is a marker for coagulation activity, and has been shown to be associated with susceptibility for migraine with aura (Guo Y. et al., 2021). In addition, ischemic stroke patients with a history of migraine with aura more often had prothrombotic genotypes (factor V Leiden and prothrombin G20210A) than ischemic stroke patients without history of migraine or migraine without aura (Pezzini et al., 2011). To further unravel the pathophysiological connection between migraine and stroke more studies investigating the influence of migraine on hemostatic factors are urgently needed.

Ischemic stroke and migraine are associated with the presence of white matter hyperintensities (WMH), which might be part of the underlying pathophysiology (Kruit et al., 2010; Ghaznawi et al., 2021). Several hemostatic factors are associated with WMH, showing possible mechanisms of hemostatic factor involvement in pathophysiology of ischemic stroke and migraine besides hypercoagulability. Increased fibrinogen levels have been associated with more severe WMH (You et al., 2018; Guo X. et al., 2021), as well as PLA2 (Zhu et al., 2019). Furthermore, increased t-PA activity was associated with progression of WMH in lacunar stroke (van Overbeek et al., 2016).

Increased levels of hemostatic factors can be both cause or consequence of acute ischemic stroke. Most factors for which a sex difference was found, were measured 1–3 months after stroke (FVII, FXI, PAI-1, and Protein S) (Mansfield et al., 1998; Kain et al., 2001, 2002; Haapaniemi et al., 2002; Saidi et al., 2007; Santamaria et al., 2007) and are, therefore, not severely influenced by the acute phase of ischemic stroke. Only two of the factors with a sex difference, aPS and P-selectin, were measured in the first week after stroke (Tuhrim et al., 1999; Blum et al., 2012). This indicates that the levels of these factors could be a direct consequence of ischemic stroke and a different response between men and women. Unfortunately, the timing of measurement was unknown for the study showing higher D-dimer levels in women (Li et al., 2018).

The reported sex differences should be interpreted with caution as many were only investigated in a limited number of studies. Most factors were measured in a single study, or in multiple studies with large heterogeneity. First, some studies did not provide information about risk factors (especially diabetes mellitus and smoking affect the hemostatic balance) for ischemic stroke and/or whether reported results were adjusted for those risk factors. Second, most studies did not provide information on the medication that patients received before or after stroke. Since all blood samples were collected after stroke, patients likely already received medication that can influence the levels of hemostatic factors such as anticoagulants. Because of this heterogeneity, a formal meta-analysis was unfortunately not possible.

To interpret the generalizability of this systematic review several factors need to be considered. First, studies included patients with varying etiologies of ischemic stroke. The underlying mechanisms of stroke might be related to (sex) differences in hemostatic factors. Some studies took differences of hemostatic factors for stroke etiologies or stroke location into account. However, the majority of them did not report results separately for men and women and had to be excluded from this review. Second, most patients were older than 50 years whereas (sex differences in) hemostatic factors may be especially important in young ischemic stroke patients, when estrogen and other sex hormones have the most influence on hemostasis related factors (Abou-Ismail et al., 2020). Third, women specific risk factors were often not reported in studies on hemostatic factors, such as pre-eclampsia and migraine. Both pre-eclampsia and migraine are associated with an inflammatory and hypercoagulable state, increasing the risk of ischemic stroke (Sacco et al., 2017; Demel et al., 2018). Only one study investigated the role of migraine on hypercoagulability in women with stroke (Salobir et al., 2002), showing the lack of research on this common women specific risk factor.

## Conclusion

Sex differences appear to exist for several hemostatic factors after ischemic stroke, with women leaning more toward increased procoagulant factors, and men leaning more toward increased coagulant inhibitors. Future research on hemostatic factors in ischemic stroke should also include young ischemic stroke patients, stratify for stroke etiology, report medication use, report results for men and women separately, include migraine as risk factor (including migraine subtype), and take women specific risk factors into account. This could improve insights about differences in mechanisms underlying ischemic stroke between men and women.

## Data Availability Statement

The original contributions presented in the study are included in the article/Supplementary Material, further inquiries can be directed to the corresponding author/s.

## Author Contributions

NW, HO, MW, BS, and JS designed the systematic search strategy. NW, MA, and HO collected the data. All authors contributed to drafting the article or revisions and approved the final version for publication.

## Conflict of Interest

The authors declare that the research was conducted in the absence of any commercial or financial relationships that could be construed as a potential conflict of interest.

## Publisher’s Note

All claims expressed in this article are solely those of the authors and do not necessarily represent those of their affiliated organizations, or those of the publisher, the editors and the reviewers. Any product that may be evaluated in this article, or claim that may be made by its manufacturer, is not guaranteed or endorsed by the publisher.
