# Supplementary material for: Sex Differences in Hemostatic Factors in Patients With Ischemic Stroke and the Relation With Migraine—A Systematic Review
Source: Front Cell Neurosci. 2021 Nov 11;15:711604. doi: 10.3389/fncel.2021.711604 (PMC8632366; doi:10.3389/fncel.2021.711604)
Supplement: Supplementary file 1 [file Data_Sheet_1.DOC]

**Appendix S1 Search strategy**

1. **Sex differences of hemostatic related factors in ischemic stroke**

**PubMed**

((("stroke"[majr] OR "stroke"[ti] OR "cva"[ti] OR "cerebrovascular accident"[ti] OR "cerebrovascular accidents"[ti] OR "cerebrovascular apoplexy"[ti] OR "apoplexy"[ti] OR "brain vascular accidents"[ti] OR "vascular accidents"[ti] OR "cerebrovascular stroke"[ti] OR "cerebrovascular strokes"[ti] OR "cerebrovascular apoplexy"[ti] OR "cerebral stroke"[ti] OR "cerebral strokes"[ti] OR "acute stroke"[ti] OR "acute strokes"[ti] OR "acute cerebrovascular accident"[ti] OR "acute cerebrovascular accidents"[ti] OR "cerebrovascular accidents"[ti] OR "brain infarction"[ti] OR "brain infarctions"[ti] OR "brain infarct"[ti] OR "brain infarcts"[ti] OR "anterior circulation infarction"[ti] OR "venous infarction"[ti] OR "venous infarctions"[ti] OR "posterior circulation infarction"[ti] OR "posterior circulation infarcts"[ti] OR "brain ischemia"[ti] OR "ischemic encephalopathy"[ti] OR "ischemic encephalopathies"[ti] OR "cerebral ischemia"[ti] OR "cerebral ischemias"[ti]) AND ("blood coagulation"[tw] OR "blood coagulation factor"[tw] OR "blood coagulation factors"[tw] OR "coagulation factor"[tw] OR "coagulation factors"[tw] OR "clotting factor"[tw] OR "clotting factors"[tw] OR "blood clotting"[tw] OR "blood clotting factor"[tw] OR "blood clotting factors"[tw] OR "thromboses"[tw] OR "thrombus"[tw] OR "blood clot"[tw] OR "blood clots"[tw] OR "thrombus formation"[tw] OR "hemostases"[tw] OR "platelet activation"[tw] OR "platelet activations"[tw] OR "platelet aggregation"[tw] OR "platelet clotting"[tw] OR "platelet adhesion"[tw] OR "Blood Coagulation Factors"[Mesh] OR "factor i"[tw] OR "blood coagulation factor i"[tw] OR "coagulation factor i"[tw] OR "fibrinogen"[tw] OR "gamma fibrinogen"[tw] OR "gamma fibrinogen"[tw] OR "fi"[tw] OR "factor ii"[tw] OR "blood coagulation factor ii"[tw] OR "differentiation reversal factor"[tw] OR "coagulation factor ii"[tw] OR "prothrombin"[tw] OR "fii"[tw] OR "thromboplastin"[tw] OR "coagulin"[tw] OR "factor iii"[tw] OR "fiii"[tw] OR "coagulation factor iii"[tw] OR "coagulation tissue factor"[tw] OR "tissue thromboplastin"[tw] OR "thromboplastin"[tw] OR "procoagulant"[tw] OR "tissue factor procoagulant"[tw] OR "coagulation factor iv"[tw] OR "factor iv"[tw] OR "calcium 40"[tw] OR "calcium"[tw] OR "fv"[tw] OR "factor five"[tw] OR "blood coagulation factor v"[tw] OR "coagulation factor v"[tw] OR "coagulation factor v"[tw] OR "factor v"[tw] OR "activated factor v"[tw] OR "coagulation factor va"[tw] OR "factor va"[tw] OR "factor v leiden"[tw] OR "factor five leiden"[tw] OR "factor seven"[tw] OR "coagulation factor vii"[tw] OR "factor vii"[tw] OR "blood coagulation factor vii"[tw] OR "proconvertin"[tw] OR "coagulation factor 7"[tw] OR "factor 7"[tw] OR "coagulation factor viia"[tw] OR "factor viia"[tw] OR "activated factor vii"[tw] OR "activated factor vii"[tw] OR "factor 7a"[tw] OR "factor 8"[tw] OR "coagulation factor viii"[tw] OR "thromboplastinogen"[tw] OR "blood coagulation factor viii"[tw] OR "factor eight"[tw] OR "hyatt c"[tw] OR "factor viiic"[tw] OR "f viii c"[tw] OR "factor viii"[tw] OR "blood coagulation factor viii"[tw] OR "coagulation factor viiia"[tw] OR "factor viiia"[tw] OR "coagulation factor viii"[tw] OR "thrombin activated factor viii"[tw] OR "factor 8a"[tw] OR "activated factor viii"[tw] OR "factor nine"[tw] OR "autoprothrombin ii"[tw] OR "christmas factor"[tw] OR "factor ix"[tw] OR "blood coagulation factor ix"[tw] OR "factor ix complex"[tw] OR "factor 9"[tw] OR "coagulation factor ix"[tw] OR "coagulation factor ixa"[tw] OR "factor ixa"[tw] OR "activated factor ix"[tw] OR "factor 9a"[tw] OR "plasma thromboplastin component"[tw] OR "factor ten"[tw] OR "autoprothrombin iii"[tw] OR "coagulation factor x"[tw] OR "factor x"[tw] OR "blood coagulation factor x"[tw] OR "stuart factor"[tw] OR "stuart prower factor"[tw] OR "factor 10"[tw] OR "activated factor x"[tw] OR "activated coagulation factor x"[tw] OR "factor xa"[tw] OR "blood coagulation factor x"[tw] OR "factor 10a"[tw] OR "autoprothrombin c"[tw] OR "factor eleven"[tw] OR "blood coagulation factor xi"[tw] OR "plasma thromboplastin"[tw] OR "plasma thromboplastin antecedent"[tw] OR "antecedent"[tw] OR "plasma thromboplastin"[tw] OR "thromboplastin antecedent"[tw] OR "coagulation factor xi"[tw] OR "factor xi"[tw] OR "coagulation factor 11"[tw] OR "factor 11"[tw] OR "activated factor xi"[tw] OR "coagulation factor xia"[tw] OR "factor xia"[tw] OR "blood coagulation factor xi"[tw] OR "factor 11a"[tw] OR "factor twelve"[tw] OR "hageman factor"[tw] OR "factor 12"[tw] OR "coagulation factor xii"[tw] OR "activated factor xii"[tw] OR "coagulation factor xiia"[tw] OR "factor xiia"[tw] OR "blood coagulation factor xii"[tw] OR "activated prekallikrein"[tw] OR "prekallikrein"[tw] OR "factor xii"[tw] OR "activated hageman factor"[tw] OR "hageman factor fragment"[tw] OR "factor thirteen"[tw] OR "coagulation factor xiii"[tw] OR "factor xiii"[tw] OR "factor xiii"[tw] OR "transamidase"[tw] OR "fibrinase"[tw] OR "laki lorand factor"[tw] OR "blood coagulation factor xiii"[tw] OR "factor 13"[tw] OR "fibrin stabilizing factor"[tw] OR "stabilizing factor"[tw] OR "activated factor xiii"[tw] OR "factor 13a"[tw] OR "plasma transglutaminase"[tw] OR "transglutaminase"[tw] OR "beta thromboglobulin"[tw] OR "beta 2 thromboglobulin"[tw] OR "thromboglobulin"[tw] OR "fibrinopeptides a"[tw] OR "fibrinopeptides b"[tw] OR "kalliginogenase"[tw] OR "kallikrein"[tw] OR "kallikrein kininogenase"[tw] OR "callicrein"[tw] OR "kinin forming enzyme"[tw] OR "kinin forming"[tw] OR "kallikrein light chain"[tw] OR "kallikrein padutin"[tw] OR "kallikrein a"[tw] OR "beta kallikrein"[tw] OR "kallikrein b"[tw] OR "alpha kallikrein"[tw] OR "plasma kallikrein"[tw] OR "kininogenin"[tw] OR "kallikrein i"[tw] OR "plasma prokallikrein"[tw] OR "prokallikrein"[tw] OR "prokinins"[tw] OR "cystatins"[tw] OR "t kininogen"[tw] OR "low molecular weight kininogens"[tw] OR "lmwk"[tw] OR "high molecular weight kininogens"[tw] OR "hmwk"[tw] OR "fitzgerald factor"[tw] OR "plasminogen"[tw] OR "plasminogen activator"[tw] OR "plasminogen activator inhibitors"[tw] OR "endothelial plasminogen activator"[tw] OR "endothelial plasminogen activator inhibitor"[tw] OR "pai 1"[tw] OR "serpin e1"[tw] OR "serpine1"[tw] OR "serpine1 protein"[tw] OR "type 1 plasminogen activator inhibitor"[tw] OR "serpin b2"[tw] OR "type 2 plasminogen activator inhibitor"[tw] OR "pai 2"[tw] OR "pai 3"[tw] OR "plasminogen activator inhibitor"[tw] OR "activated protein c inhibitor"[tw] OR "plasminogen activator inhibitor 3"[tw] OR "phosphorylcholine"[tw] OR "acetyl glyceryl phosphorylcholine"[tw] OR "aggregating factor"[tw] OR "platelet aggregation enhancing factor"[tw] OR "agepc"[tw] OR "thrombocyte aggregating activity"[tw] OR "alkyl 2 acetyl sn glycerophosphocholine"[tw] OR "1 alkyl 2 acetyl sn glyceryl 3 phosphorylcholine"[tw] OR "1 alkyl 2 acetylglycerophosphocholine"[tw] OR "paf acether"[tw] OR "platelet activating substance"[tw] OR "platelet activating substances"[tw] OR "pf 3"[tw] OR "platelet factor 3"[tw] OR "chemokine cxcl4"[tw] OR "cxcl4"[tw] OR "chemokine pf4"[tw] OR "platelet factor 4"[tw] OR "heparin neutralizing protein"[tw] OR "antiheparin factor"[tw] OR "thrombase"[tw] OR "thrombostat"[tw] OR "alpha thrombin"[tw] OR "thrombin jmi"[tw] OR "jmi"[tw] OR "thrombin"[tw] OR "beta thrombin"[tw] OR "gamma thrombin"[tw] OR "plasminogen activator"[tw] OR "tissue activator d 44"[tw] OR "tisokinase"[tw] OR "tissue type plasminogen activator"[tw] OR "ttpa"[tw] OR "t plasminogen activator"[tw] OR "tissue type activator"[tw] OR "rt pa"[tw] OR "factor viiir ag"[tw] OR "factor viiir rco"[tw] OR "ristocetin willebrand factor"[tw] OR "von willebrand protein"[tw] OR "von willebrand factor"[tw] OR "factor viii related antigen"[tw] OR "ristocetin cofactor"[tw] OR "plasma factor viii complex"[tw] OR "vitamin k dependent protein s"[tw] OR "cofactor protein s"[tw] OR "protein s"[tw] OR "protein c"[tw] OR "antiphospholipid"[tw] OR "glutamate"[tw] OR "beta 2 glycoprotein"[tw] OR "anti beta 2 glycoprotein"[tw] OR "adp"[tw] OR "adenosine diphosphate"[tw] OR "adenosine pyrophosphate"[tw] OR "adenosine 5' pyrophosphate"[tw] OR "serotonin"[tw] OR "5 hydroxytryptamine"[tw] OR "hippophaine"[tw] OR "3 2 aminoethyl 2 methyl 1 h indol 5 ol"[tw] OR "enteramine"[tw] OR "5 ht"[tw] OR "hydroxytryptamine"[tw] OR "thromboxane a2"[tw] OR "txa2"[tw] OR "arachidonic acid"[tw] OR "aa"[tw] OR "acetylsalicylic acid"[tw] OR "aspirin"[tw] OR "platelet derived growth factor"[tw] OR "pdgf receptor"[tw] OR "pdgf"[tw] OR "receptor tgf beta"[tw] OR "tgfbeta"[tw] OR "tgf beta"[tw] OR "platelet transforming growth factor"[tw] OR "gmp 140"[tw] OR "p selectin"[tw] OR "padgem"[tw] OR "cd62p antigen"[tw] OR "cd62p antigens"[tw] OR "cd62p"[tw] OR "alpha granule membrane protein"[tw] OR "lecam3"[tw] OR "gpvi"[tw] OR "platelet membrane glycoprotein p62"[tw] OR "glycoprotein gp vi"[tw] OR "platelet glycoprotein vi"[tw] OR "human glycoprotein vi"[tw] OR "human alpha2beta1"[tw] OR "integrin vla 2"[tw] OR "vla 2"[tw] OR "glycoprotein ia iia"[tw] OR "alpha2beta1integrin"[tw] OR "very late antigen 2"[tw] OR "late antigen 2"[tw] OR "cd49b cd29"[tw] OR "platelet membrane glycoprotein ia/iia"[tw] OR "platelet membrane glycoprotein ia iia"[tw] OR "platelet membrane glycoprotein ia/iia complex"[tw] OR "platelet membrane glycoprotein ia iia complex"[tw] OR "platelet glycoprotein gpiib iiia complex"[tw] OR "glycoproteins iib iiia"[tw] OR "glycoproteins iib iiia complex"[tw] OR "integrin alpha iib"[tw] OR "integrin alpha iib beta 3"[tw] OR "platelet glycoprotein gpib ix complex"[tw] OR "platelet glycoprotein gpib ix complex"[tw] OR "glycoprotein ib ix complex"[tw] OR "platelet membrane glycoprotein ib"[tw] OR "glycoprotein ib beta"[tw] OR "cd42c"[tw] OR "calcitonin gene related peptide"[tw] OR "calcitonin gene related peptide ii"[tw] OR "beta cgrp"[tw] OR "beta calcitonin gene related peptide"[tw] OR "alpha cgrp"[tw] OR "alpha calcitonin gene related peptide"[tw] OR "calcitonin gene related peptide i"[tw] OR "cgrp"[tw] OR "cyclo oxygenase i"[tw] OR "cox1"[tw] OR "prostaglandin h synthase 1"[tw] OR "prostaglandin synthase cyclooxygenase"[tw] OR "prostaglandin synthase"[tw] OR "cox 1 synthesis"[tw] OR "cox 1 prostaglandin"[tw] OR "cyclooxygenase 1"[tw] OR "endothelium derived vasoconstrictor factors"[tw] OR "vasoconstrictor factors"[tw] OR "endothelium derived endothelin 1"[tw] OR "nitric nitrogen"[tw] OR "endothelium derived nitric oxide"[tw] OR "vasodilator nitrates"[tw] OR "vasodilator nitric oxide"[tw] OR "von willebrand factor cleaving protease"[tw] OR "adamts13"[tw] OR "vwf cleaving protease"[tw] OR "vwf cleaving protease adamts13"[tw] OR "p2y12 receptors"[tw] OR "p2y12 receptor"[tw] OR "p2y12"[tw] OR "purinergic p2y12 receptors"[tw] OR "purinergic p2y12 receptor"[tw] OR "purinergic receptor p2y"[tw] OR "p2y adp receptor"[tw] OR "p2y adp receptors"[tw] OR "p selectin glycoprotein ligand 1"[tw] OR "p selectin"[tw] OR CD162[tw] OR "platelet p selectin"[tw] OR "psgl 1 protein"[tw] OR "selectin P ligand protein"[tw] OR "endocannabinoid"[tw] OR "endocannabinoids"[tw] OR "cb2 receptor"[tw] OR "cannabinoid receptor cb2"[tw] OR "cannabinoid receptor 2"[tw] OR "cannabinoid receptor"[tw] OR "cannabinoid cb1 receptor"[tw] OR "cannabinoid cb2 receptor"[tw] OR "cannabinoid receptor cb1"[tw] OR "cannabinoid receptor 1"[tw] OR "cb1 cannabinoid receptor"[tw] OR "cb2 cannabinoid receptor"[tw] OR "glyceryl 2 arachidonate"[tw] OR "2 ag"[tw] OR "aea"[tw] OR "2 arachidonoylglycerol"[tw] OR "anandamide"[tw] OR "n arachidonoylethanolamine"[tw] OR "metalloproteinase 9"[tw] OR "matrix metalloproteinase 9"[tw] OR "mmp9"[tw] OR "metalloproteinase"[tw] OR "metallopeptidases"[tw] OR "metalloproteinases"[tw] OR "metallopeptidase"[tw] OR "type iv collagenase"[tw] OR "type iv collagenase mmp 2"[tw] OR "type iv collagenase mmp 9"[tw] OR "metalloproteinase mmp 9"[tw] OR "metalloproteinase 1"[tw] OR "mmp1"[tw] OR "metalloproteinase mmp1"[tw] OR "mmp 1"[tw] OR "mmp 9"[tw] OR "matrix metalloproteinase 1"[tw] OR "metalloproteinase 3"[tw] OR "mmp3"[tw] OR "matrix metalloproteinase 3"[tw] OR "metalloproteinase 2"[tw] OR "mmp2"[tw] OR "matrix metalloproteinase 2"[tw] OR "type iv collagenase mmp 2"[tw] OR "mmp 2 gelatinase a"[tw]) AND ("Sex"[Mesh] OR "Gender"[tw] OR "Sex"[tiab] OR "Sex Characteristics"[Mesh] OR Sex Characteristic*[tw] OR Sex Difference*[tw] OR Gender Difference*[tw] OR "Sex Based"[tw] OR Sex Dimorphism*[tw] OR Sexual Dimorphism*[tw] OR (("Women"[mesh] OR "Women"[tw] OR "Woman"[tw] OR "Female"[tiab]) AND ("Men"[mesh] OR "Men"[tw] OR "Man"[tw] OR "Male"[tiab])))) OR (("stroke"[majr] OR "stroke"[ti] OR "cva"[ti] OR "cerebrovascular accident"[ti] OR "cerebrovascular accidents"[ti] OR "cerebrovascular apoplexy"[ti] OR "apoplexy"[ti] OR "brain vascular accidents"[ti] OR "vascular accidents"[ti] OR "cerebrovascular stroke"[ti] OR "cerebrovascular strokes"[ti] OR "cerebrovascular apoplexy"[ti] OR "cerebral stroke"[ti] OR "cerebral strokes"[ti] OR "acute stroke"[ti] OR "acute strokes"[ti] OR "acute cerebrovascular accident"[ti] OR "acute cerebrovascular accidents"[ti] OR "cerebrovascular accidents"[ti] OR "brain infarction"[ti] OR "brain infarctions"[ti] OR "brain infarct"[ti] OR "brain infarcts"[ti] OR "anterior circulation infarction"[ti] OR "venous infarction"[ti] OR "venous infarctions"[ti] OR "posterior circulation infarction"[ti] OR "posterior circulation infarcts"[ti] OR "brain ischemia"[ti] OR "ischemic encephalopathy"[ti] OR "ischemic encephalopathies"[ti] OR "cerebral ischemia"[ti] OR "cerebral ischemias"[ti]) AND ("blood coagulation"[ti] OR "blood coagulation factor"[ti] OR "blood coagulation factors"[ti] OR "coagulation factor"[ti] OR "coagulation factors"[ti] OR "clotting factor"[ti] OR "clotting factors"[ti] OR "blood clotting"[ti] OR "blood clotting factor"[ti] OR "blood clotting factors"[ti] OR "thromboses"[ti] OR "thrombus"[ti] OR "blood clot"[ti] OR "blood clots"[ti] OR "thrombus formation"[ti] OR "hemostases"[ti] OR "platelet activation"[ti] OR "platelet activations"[ti] OR "platelet aggregation"[ti] OR "platelet clotting"[ti] OR "platelet adhesion"[ti] OR "Blood Coagulation Factors"[majr] OR "factor i"[ti] OR "blood coagulation factor i"[ti] OR "coagulation factor i"[ti] OR "fibrinogen"[ti] OR "gamma fibrinogen"[ti] OR "gamma fibrinogen"[ti] OR "fi"[ti] OR "factor ii"[ti] OR "blood coagulation factor ii"[ti] OR "differentiation reversal factor"[ti] OR "coagulation factor ii"[ti] OR "prothrombin"[ti] OR "fii"[ti] OR "thromboplastin"[ti] OR "coagulin"[ti] OR "factor iii"[ti] OR "fiii"[ti] OR "coagulation factor iii"[ti] OR "coagulation tissue factor"[ti] OR "tissue thromboplastin"[ti] OR "thromboplastin"[ti] OR "procoagulant"[ti] OR "tissue factor procoagulant"[ti] OR "coagulation factor iv"[ti] OR "factor iv"[ti] OR "calcium 40"[ti] OR "calcium"[ti] OR "fv"[ti] OR "factor five"[ti] OR "blood coagulation factor v"[ti] OR "coagulation factor v"[ti] OR "coagulation factor v"[ti] OR "factor v"[ti] OR "activated factor v"[ti] OR "coagulation factor va"[ti] OR "factor va"[ti] OR "factor v leiden"[ti] OR "factor five leiden"[ti] OR "factor seven"[ti] OR "coagulation factor vii"[ti] OR "factor vii"[ti] OR "blood coagulation factor vii"[ti] OR "proconvertin"[ti] OR "coagulation factor 7"[ti] OR "factor 7"[ti] OR "coagulation factor viia"[ti] OR "factor viia"[ti] OR "activated factor vii"[ti] OR "activated factor vii"[ti] OR "factor 7a"[ti] OR "factor 8"[ti] OR "coagulation factor viii"[ti] OR "thromboplastinogen"[ti] OR "blood coagulation factor viii"[ti] OR "factor eight"[ti] OR "hyatt c"[ti] OR "factor viiic"[ti] OR "f viii c"[ti] OR "factor viii"[ti] OR "blood coagulation factor viii"[ti] OR "coagulation factor viiia"[ti] OR "factor viiia"[ti] OR "coagulation factor viii"[ti] OR "thrombin activated factor viii"[ti] OR "factor 8a"[ti] OR "activated factor viii"[ti] OR "factor nine"[ti] OR "autoprothrombin ii"[ti] OR "christmas factor"[ti] OR "factor ix"[ti] OR "blood coagulation factor ix"[ti] OR "factor ix complex"[ti] OR "factor 9"[ti] OR "coagulation factor ix"[ti] OR "coagulation factor ixa"[ti] OR "factor ixa"[ti] OR "activated factor ix"[ti] OR "factor 9a"[ti] OR "plasma thromboplastin component"[ti] OR "factor ten"[ti] OR "autoprothrombin iii"[ti] OR "coagulation factor x"[ti] OR "factor x"[ti] OR "blood coagulation factor x"[ti] OR "stuart factor"[ti] OR "stuart prower factor"[ti] OR "factor 10"[ti] OR "activated factor x"[ti] OR "activated coagulation factor x"[ti] OR "factor xa"[ti] OR "blood coagulation factor x"[ti] OR "factor 10a"[ti] OR "autoprothrombin c"[ti] OR "factor eleven"[ti] OR "blood coagulation factor xi"[ti] OR "plasma thromboplastin"[ti] OR "plasma thromboplastin antecedent"[ti] OR "antecedent"[ti] OR "plasma thromboplastin"[ti] OR "thromboplastin antecedent"[ti] OR "coagulation factor xi"[ti] OR "factor xi"[ti] OR "coagulation factor 11"[ti] OR "factor 11"[ti] OR "activated factor xi"[ti] OR "coagulation factor xia"[ti] OR "factor xia"[ti] OR "blood coagulation factor xi"[ti] OR "factor 11a"[ti] OR "factor twelve"[ti] OR "hageman factor"[ti] OR "factor 12"[ti] OR "coagulation factor xii"[ti] OR "activated factor xii"[ti] OR "coagulation factor xiia"[ti] OR "factor xiia"[ti] OR "blood coagulation factor xii"[ti] OR "activated prekallikrein"[ti] OR "prekallikrein"[ti] OR "factor xii"[ti] OR "activated hageman factor"[ti] OR "hageman factor fragment"[ti] OR "factor thirteen"[ti] OR "coagulation factor xiii"[ti] OR "factor xiii"[ti] OR "factor xiii"[ti] OR "transamidase"[ti] OR "fibrinase"[ti] OR "laki lorand factor"[ti] OR "blood coagulation factor xiii"[ti] OR "factor 13"[ti] OR "fibrin stabilizing factor"[ti] OR "stabilizing factor"[ti] OR "activated factor xiii"[ti] OR "factor 13a"[ti] OR "plasma transglutaminase"[ti] OR "transglutaminase"[ti] OR "beta thromboglobulin"[ti] OR "beta 2 thromboglobulin"[ti] OR "thromboglobulin"[ti] OR "fibrinopeptides a"[tw] OR "fibrinopeptides b"[ti] OR "kalliginogenase"[ti] OR "kallikrein"[ti] OR "kallikrein kininogenase"[ti] OR "callicrein"[ti] OR "kinin forming enzyme"[ti] OR "kinin forming"[ti] OR "kallikrein light chain"[ti] OR "kallikrein padutin"[ti] OR "kallikrein a"[ti] OR "beta kallikrein"[ti] OR "kallikrein b"[ti] OR "alpha kallikrein"[ti] OR "plasma kallikrein"[ti] OR "kininogenin"[ti] OR "kallikrein i"[ti] OR "plasma prokallikrein"[ti] OR "prokallikrein"[ti] OR "prokinins"[ti] OR "cystatins"[ti] OR "t kininogen"[ti] OR "low molecular weight kininogens"[ti] OR "lmwk"[ti] OR "high molecular weight kininogens"[ti] OR "hmwk"[ti] OR "fitzgerald factor"[ti] OR "plasminogen"[ti] OR "plasminogen activator"[ti] OR "plasminogen activator inhibitors"[ti] OR "endothelial plasminogen activator"[ti] OR "endothelial plasminogen activator inhibitor"[ti] OR "pai 1"[ti] OR "serpin e1"[ti] OR "serpine1"[ti] OR "serpine1 protein"[ti] OR "type 1 plasminogen activator inhibitor"[ti] OR "serpin b2"[ti] OR "type 2 plasminogen activator inhibitor"[ti] OR "pai 2"[ti] OR "pai 3"[ti] OR "plasminogen activator inhibitor"[ti] OR "activated protein c inhibitor"[ti] OR "plasminogen activator inhibitor 3"[ti] OR "phosphorylcholine"[ti] OR "acetyl glyceryl phosphorylcholine"[ti] OR "aggregating factor"[ti] OR "platelet aggregation enhancing factor"[ti] OR "agepc"[ti] OR "thrombocyte aggregating activity"[ti] OR "alkyl 2 acetyl sn glycerophosphocholine"[ti] OR "1 alkyl 2 acetyl sn glyceryl 3 phosphorylcholine"[ti] OR "1 alkyl 2 acetylglycerophosphocholine"[ti] OR "paf acether"[ti] OR "platelet activating substance"[ti] OR "platelet activating substances"[ti] OR "pf 3"[ti] OR "platelet factor 3"[ti] OR "chemokine cxcl4"[ti] OR "cxcl4"[ti] OR "chemokine pf4"[ti] OR "platelet factor 4"[ti] OR "heparin neutralizing protein"[ti] OR "antiheparin factor"[ti] OR "thrombase"[ti] OR "thrombostat"[ti] OR "alpha thrombin"[ti] OR "thrombin jmi"[ti] OR "jmi"[ti] OR "thrombin"[ti] OR "beta thrombin"[ti] OR "gamma thrombin"[ti] OR "plasminogen activator"[ti] OR "tissue activator d 44"[ti] OR "tisokinase"[ti] OR "tissue type plasminogen activator"[ti] OR "ttpa"[ti] OR "t plasminogen activator"[ti] OR "tissue type activator"[ti] OR "rt pa"[ti] OR "factor viiir ag"[ti] OR "factor viiir rco"[ti] OR "ristocetin willebrand factor"[ti] OR "von willebrand protein"[ti] OR "von willebrand factor"[ti] OR "factor viii related antigen"[ti] OR "ristocetin cofactor"[ti] OR "plasma factor viii complex"[ti] OR "vitamin k dependent protein s"[ti] OR "cofactor protein s"[ti] OR "protein s"[ti] OR "protein c"[ti] OR "antiphospholipid"[tw] OR "glutamate"[tw] OR "beta 2 glycoprotein"[tw] OR "anti beta 2 glycoprotein"[tw] OR "adp"[ti] OR "adenosine diphosphate"[ti] OR "adenosine pyrophosphate"[ti] OR "adenosine 5' pyrophosphate"[ti] OR "serotonin"[ti] OR "5 hydroxytryptamine"[ti] OR "hippophaine"[ti] OR "3 2 aminoethyl 2 methyl 1 h indol 5 ol"[ti] OR "enteramine"[ti] OR "5 ht"[ti] OR "hydroxytryptamine"[ti] OR "thromboxane a2"[ti] OR "txa2"[ti] OR "arachidonic acid"[ti] OR "aa"[ti] OR "acetylsalicylic acid"[ti] OR "aspirin"[ti] OR "platelet derived growth factor"[ti] OR "pdgf receptor"[ti] OR "pdgf"[ti] OR "receptor tgf beta"[ti] OR "tgfbeta"[ti] OR "tgf beta"[ti] OR "platelet transforming growth factor"[ti] OR "gmp 140"[ti] OR "p selectin"[ti] OR "padgem"[ti] OR "cd62p antigen"[ti] OR "cd62p antigens"[ti] OR "cd62p"[ti] OR "alpha granule membrane protein"[ti] OR "lecam3"[ti] OR "gpvi"[ti] OR "platelet membrane glycoprotein p62"[ti] OR "glycoprotein gp vi"[ti] OR "platelet glycoprotein vi"[ti] OR "human glycoprotein vi"[ti] OR "human alpha2beta1"[ti] OR "integrin vla 2"[ti] OR "vla 2"[ti] OR "glycoprotein ia iia"[ti] OR "alpha2beta1integrin"[ti] OR "very late antigen 2"[ti] OR "late antigen 2"[ti] OR "cd49b cd29"[ti] OR "platelet membrane glycoprotein ia/iia"[ti] OR "platelet membrane glycoprotein ia iia"[ti] OR "platelet membrane glycoprotein ia/iia complex"[ti] OR "platelet membrane glycoprotein ia iia complex"[ti] OR "platelet glycoprotein gpiib iiia complex"[ti] OR "glycoproteins iib iiia"[ti] OR "glycoproteins iib iiia complex"[ti] OR "integrin alpha iib"[ti] OR "integrin alpha iib beta 3"[ti] OR "platelet glycoprotein gpib ix complex"[ti] OR "platelet glycoprotein gpib ix complex"[ti] OR "glycoprotein ib ix complex"[ti] OR "platelet membrane glycoprotein ib"[ti] OR "glycoprotein ib beta"[ti] OR "cd42c"[ti] OR "calcitonin gene related peptide"[ti] OR "calcitonin gene related peptide ii"[ti] OR "beta cgrp"[ti] OR "beta calcitonin gene related peptide"[ti] OR "alpha cgrp"[ti] OR "alpha calcitonin gene related peptide"[ti] OR "calcitonin gene related peptide i"[ti] OR "cgrp"[ti] OR "cyclo oxygenase i"[ti] OR "cox1"[ti] OR "prostaglandin h synthase 1"[ti] OR "prostaglandin synthase cyclooxygenase"[ti] OR "prostaglandin synthase"[ti] OR "cox 1 synthesis"[ti] OR "cox 1 prostaglandin"[ti] OR "cyclooxygenase 1"[ti] OR "endothelium derived vasoconstrictor factors"[ti] OR "vasoconstrictor factors"[ti] OR "endothelium derived endothelin 1"[ti] OR "nitric nitrogen"[ti] OR "endothelium derived nitric oxide"[ti] OR "vasodilator nitrates"[ti] OR "vasodilator nitric oxide"[ti] OR "von willebrand factor cleaving protease"[ti] OR "adamts13"[ti] OR "vwf cleaving protease"[ti] OR "vwf cleaving protease adamts13"[ti] OR "p2y12 receptors"[ti] OR "p2y12 receptor"[ti] OR "p2y12"[ti] OR "purinergic p2y12 receptors"[ti] OR "purinergic p2y12 receptor"[ti] OR "purinergic receptor p2y"[ti] OR "p2y adp receptor"[ti] OR "p2y adp receptors"[ti] OR "p selectin glycoprotein ligand 1"[ti] OR "p selectin"[ti] OR CD162[ti] OR "platelet p selectin"[ti] OR "psgl 1 protein"[ti] OR "selectin P ligand protein"[ti] OR "endocannabinoid"[ti] OR "endocannabinoids"[ti] OR "cb2 receptor"[ti] OR "cannabinoid receptor cb2"[ti] OR "cannabinoid receptor 2"[ti] OR "cannabinoid receptor"[ti] OR "cannabinoid cb1 receptor"[ti] OR "cannabinoid cb2 receptor"[ti] OR "cannabinoid receptor cb1"[ti] OR "cannabinoid receptor 1"[ti] OR "cb1 cannabinoid receptor"[ti] OR "cb2 cannabinoid receptor"[ti] OR "glyceryl 2 arachidonate"[ti] OR "2 ag"[ti] OR "aea"[ti] OR "2 arachidonoylglycerol"[ti] OR "anandamide"[ti] OR "n arachidonoylethanolamine"[ti] OR "metalloproteinase 9"[ti] OR "matrix metalloproteinase 9"[ti] OR "mmp9"[ti] OR "metalloproteinase"[ti] OR "metallopeptidases"[ti] OR "metalloproteinases"[ti] OR "metallopeptidase"[ti] OR "type iv collagenase"[ti] OR "type iv collagenase mmp 2"[ti] OR "type iv collagenase mmp 9"[ti] OR "metalloproteinase mmp 9"[ti] OR "metalloproteinase 1"[ti] OR "mmp1"[ti] OR "metalloproteinase mmp1"[ti] OR "mmp 1"[ti] OR "mmp 9"[ti] OR "matrix metalloproteinase 1"[ti] OR "metalloproteinase 3"[ti] OR "mmp3"[ti] OR "matrix metalloproteinase 3"[ti] OR "metalloproteinase 2"[ti] OR "mmp2"[ti] OR "matrix metalloproteinase 2"[ti] OR "type iv collagenase mmp 2"[ti] OR "mmp 2 gelatinase a"[ti]) AND ("Sex"[Mesh] OR "Gender"[tw] OR "Sex"[tiab] OR "Sex Characteristics"[Mesh] OR Sex Characteristic*[tw] OR Sex Difference*[tw] OR Gender Difference*[tw] OR "Sex Based"[tw] OR Sex Dimorphism*[tw] OR Sexual Dimorphism*[tw] OR (("Women"[mesh] OR "Women"[tw] OR "Woman"[tw] OR "Female"[tiab]) AND ("Men"[mesh] OR "Men"[tw] OR "Man"[tw] OR "Male"[tiab])))) NOT ("Tissue Plasminogen Activator/pharmacology"[Mesh] OR "Aspirin/pharmacology"[Mesh] OR "Stroke/therapy"[Mesh])) NOT (("Case Reports"[ptyp] OR "case report"[ti] OR "Review"[ptyp] OR "review"[ti] OR "systematic"[sb] OR "systematic review"[ti] OR "meta-analysis"[ptyp] OR "meta-analysis"[ti]) NOT ("Clinical Study"[ptyp] OR "trial"[ti] OR "RCT"[ti])) NOT ("Stroke/therapy"[majr] OR therap*[ti] OR treat*[ti]) AND (english[la] OR dutch[la]) NOT (("Child"[mesh] OR "Infant"[mesh]) NOT ("Adult"[mesh]))

**Embase**

(((exp *"cerebrovascular accident"/ OR "stroke".ti OR "cva".ti OR "cerebrovascular accident".ti OR "cerebrovascular accidents".ti OR "cerebrovascular apoplexy".ti OR "apoplexy".ti OR "brain vascular accidents".ti OR "vascular accidents".ti OR "cerebrovascular stroke".ti OR "cerebrovascular strokes".ti OR "cerebrovascular apoplexy".ti OR "cerebral stroke".ti OR "cerebral strokes".ti OR "acute stroke".ti OR "acute strokes".ti OR "acute cerebrovascular accident".ti OR "acute cerebrovascular accidents".ti OR "cerebrovascular accidents".ti OR "brain infarction".ti OR "brain infarctions".ti OR "brain infarct".ti OR "brain infarcts".ti OR "anterior circulation infarction".ti OR "venous infarction".ti OR "venous infarctions".ti OR "posterior circulation infarction".ti OR "posterior circulation infarcts".ti OR "brain ischemia".ti OR "ischemic encephalopathy".ti OR "ischemic encephalopathies".ti OR "cerebral ischemia".ti OR "cerebral ischemias".ti) AND ("blood coagulation".ti,ab OR "blood coagulation factor".ti,ab OR "blood coagulation factors".ti,ab OR "coagulation factor".ti,ab OR "coagulation factors".ti,ab OR "clotting factor".ti,ab OR "clotting factors".ti,ab OR "blood clotting".ti,ab OR "blood clotting factor".ti,ab OR "blood clotting factors".ti,ab OR "thromboses".ti,ab OR "thrombus".ti,ab OR "blood clot".ti,ab OR "blood clots".ti,ab OR "thrombus formation".ti,ab OR "hemostases".ti,ab OR "platelet activation".ti,ab OR "platelet activations".ti,ab OR "platelet aggregation".ti,ab OR "platelet clotting".ti,ab OR "platelet adhesion".ti,ab OR exp *"Blood Clotting Factor"/ OR "factor i".ti,ab OR "blood coagulation factor i".ti,ab OR "coagulation factor i".ti,ab OR "fibrinogen".ti,ab OR "gamma fibrinogen".ti,ab OR "gamma fibrinogen".ti,ab OR "fi".ti,ab OR "factor ii".ti,ab OR "blood coagulation factor ii".ti,ab OR "differentiation reversal factor".ti,ab OR "coagulation factor ii".ti,ab OR "prothrombin".ti,ab OR "fii".ti,ab OR "thromboplastin".ti,ab OR "coagulin".ti,ab OR "factor iii".ti,ab OR "fiii".ti,ab OR "coagulation factor iii".ti,ab OR "coagulation tissue factor".ti,ab OR "tissue thromboplastin".ti,ab OR "thromboplastin".ti,ab OR "procoagulant".ti,ab OR "tissue factor procoagulant".ti,ab OR "coagulation factor iv".ti,ab OR "factor iv".ti,ab OR "calcium 40".ti,ab OR "calcium".ti,ab OR "fv".ti,ab OR "factor five".ti,ab OR "blood coagulation factor v".ti,ab OR "coagulation factor v".ti,ab OR "coagulation factor v".ti,ab OR "factor v".ti,ab OR "activated factor v".ti,ab OR "coagulation factor va".ti,ab OR "factor va".ti,ab OR "factor v leiden".ti,ab OR "factor five leiden".ti,ab OR "factor seven".ti,ab OR "coagulation factor vii".ti,ab OR "factor vii".ti,ab OR "blood coagulation factor vii".ti,ab OR "proconvertin".ti,ab OR "coagulation factor 7".ti,ab OR "factor 7".ti,ab OR "coagulation factor viia".ti,ab OR "factor viia".ti,ab OR "activated factor vii".ti,ab OR "activated factor vii".ti,ab OR "factor 7a".ti,ab OR "factor 8".ti,ab OR "coagulation factor viii".ti,ab OR "thromboplastinogen".ti,ab OR "blood coagulation factor viii".ti,ab OR "factor eight".ti,ab OR "hyatt c".ti,ab OR "factor viiic".ti,ab OR "f viii c".ti,ab OR "factor viii".ti,ab OR "blood coagulation factor viii".ti,ab OR "coagulation factor viiia".ti,ab OR "factor viiia".ti,ab OR "coagulation factor viii".ti,ab OR "thrombin activated factor viii".ti,ab OR "factor 8a".ti,ab OR "activated factor viii".ti,ab OR "factor nine".ti,ab OR "autoprothrombin ii".ti,ab OR "christmas factor".ti,ab OR "factor ix".ti,ab OR "blood coagulation factor ix".ti,ab OR "factor ix complex".ti,ab OR "factor 9".ti,ab OR "coagulation factor ix".ti,ab OR "coagulation factor ixa".ti,ab OR "factor ixa".ti,ab OR "activated factor ix".ti,ab OR "factor 9a".ti,ab OR "plasma thromboplastin component".ti,ab OR "factor ten".ti,ab OR "autoprothrombin iii".ti,ab OR "coagulation factor x".ti,ab OR "factor x".ti,ab OR "blood coagulation factor x".ti,ab OR "stuart factor".ti,ab OR "stuart prower factor".ti,ab OR "factor 10".ti,ab OR "activated factor x".ti,ab OR "activated coagulation factor x".ti,ab OR "factor xa".ti,ab OR "blood coagulation factor x".ti,ab OR "factor 10a".ti,ab OR "autoprothrombin c".ti,ab OR "factor eleven".ti,ab OR "blood coagulation factor xi".ti,ab OR "plasma thromboplastin".ti,ab OR "plasma thromboplastin antecedent".ti,ab OR "antecedent".ti,ab OR "plasma thromboplastin".ti,ab OR "thromboplastin antecedent".ti,ab OR "coagulation factor xi".ti,ab OR "factor xi".ti,ab OR "coagulation factor 11".ti,ab OR "factor 11".ti,ab OR "activated factor xi".ti,ab OR "coagulation factor xia".ti,ab OR "factor xia".ti,ab OR "blood coagulation factor xi".ti,ab OR "factor 11a".ti,ab OR "factor twelve".ti,ab OR "hageman factor".ti,ab OR "factor 12".ti,ab OR "coagulation factor xii".ti,ab OR "activated factor xii".ti,ab OR "coagulation factor xiia".ti,ab OR "factor xiia".ti,ab OR "blood coagulation factor xii".ti,ab OR "activated prekallikrein".ti,ab OR "prekallikrein".ti,ab OR "factor xii".ti,ab OR "activated hageman factor".ti,ab OR "hageman factor fragment".ti,ab OR "factor thirteen".ti,ab OR "coagulation factor xiii".ti,ab OR "factor xiii".ti,ab OR "factor xiii".ti,ab OR "transamidase".ti,ab OR "fibrinase".ti,ab OR "laki lorand factor".ti,ab OR "blood coagulation factor xiii".ti,ab OR "factor 13".ti,ab OR "fibrin stabilizing factor".ti,ab OR "stabilizing factor".ti,ab OR "activated factor xiii".ti,ab OR "factor 13a".ti,ab OR "plasma transglutaminase".ti,ab OR "transglutaminase".ti,ab OR "beta thromboglobulin".ti,ab OR "beta 2 thromboglobulin".ti,ab OR "thromboglobulin".ti,ab OR "fibrinopeptides a".ti,ab OR "fibrinopeptides b".ti,ab OR "kalliginogenase".ti,ab OR "kallikrein".ti,ab OR "kallikrein kininogenase".ti,ab OR "callicrein".ti,ab OR "kinin forming enzyme".ti,ab OR "kinin forming".ti,ab OR "kallikrein light chain".ti,ab OR "kallikrein padutin".ti,ab OR "kallikrein a".ti,ab OR "beta kallikrein".ti,ab OR "kallikrein b".ti,ab OR "alpha kallikrein".ti,ab OR "plasma kallikrein".ti,ab OR "kininogenin".ti,ab OR "kallikrein i".ti,ab OR "plasma prokallikrein".ti,ab OR "prokallikrein".ti,ab OR "prokinins".ti,ab OR "cystatins".ti,ab OR "t kininogen".ti,ab OR "low molecular weight kininogens".ti,ab OR "lmwk".ti,ab OR "high molecular weight kininogens".ti,ab OR "hmwk".ti,ab OR "fitzgerald factor".ti,ab OR "plasminogen".ti,ab OR "plasminogen activator".ti,ab OR "plasminogen activator inhibitors".ti,ab OR "endothelial plasminogen activator".ti,ab OR "endothelial plasminogen activator inhibitor".ti,ab OR "pai 1".ti,ab OR "serpin e1".ti,ab OR "serpine1".ti,ab OR "serpine1 protein".ti,ab OR "type 1 plasminogen activator inhibitor".ti,ab OR "serpin b2".ti,ab OR "type 2 plasminogen activator inhibitor".ti,ab OR "pai 2".ti,ab OR "pai 3".ti,ab OR "plasminogen activator inhibitor".ti,ab OR "activated protein c inhibitor".ti,ab OR "plasminogen activator inhibitor 3".ti,ab OR "phosphorylcholine".ti,ab OR "acetyl glyceryl phosphorylcholine".ti,ab OR "aggregating factor".ti,ab OR "platelet aggregation enhancing factor".ti,ab OR "agepc".ti,ab OR "thrombocyte aggregating activity".ti,ab OR "alkyl 2 acetyl sn glycerophosphocholine".ti,ab OR "1 alkyl 2 acetyl sn glyceryl 3 phosphorylcholine".ti,ab OR "1 alkyl 2 acetylglycerophosphocholine".ti,ab OR "paf acether".ti,ab OR "platelet activating substance".ti,ab OR "platelet activating substances".ti,ab OR "pf 3".ti,ab OR "platelet factor 3".ti,ab OR "chemokine cxcl4".ti,ab OR "cxcl4".ti,ab OR "chemokine pf4".ti,ab OR "platelet factor 4".ti,ab OR "heparin neutralizing protein".ti,ab OR "antiheparin factor".ti,ab OR "thrombase".ti,ab OR "thrombostat".ti,ab OR "alpha thrombin".ti,ab OR "thrombin jmi".ti,ab OR "jmi".ti,ab OR "thrombin".ti,ab OR "beta thrombin".ti,ab OR "gamma thrombin".ti,ab OR "plasminogen activator".ti,ab OR "tissue activator d 44".ti,ab OR "tisokinase".ti,ab OR "tissue type plasminogen activator".ti,ab OR "ttpa".ti,ab OR "t plasminogen activator".ti,ab OR "tissue type activator".ti,ab OR "rt pa".ti,ab OR "factor viiir ag".ti,ab OR "factor viiir rco".ti,ab OR "ristocetin willebrand factor".ti,ab OR "von willebrand protein".ti,ab OR "von willebrand factor".ti,ab OR "factor viii related antigen".ti,ab OR "ristocetin cofactor".ti,ab OR "plasma factor viii complex".ti,ab OR "vitamin k dependent protein s".ti,ab OR "cofactor protein s".ti,ab OR "protein s".ti,ab OR "protein c".ti,ab OR "antiphospholipid".ti,ab OR "glutamate".ti,ab OR "beta 2 glycoprotein".ti,ab OR "anti beta 2 glycoprotein".ti,ab OR "adp".ti,ab OR "adenosine diphosphate".ti,ab OR "adenosine pyrophosphate".ti,ab OR "adenosine 5' pyrophosphate".ti,ab OR "serotonin".ti,ab OR "5 hydroxytryptamine".ti,ab OR "hippophaine".ti,ab OR "3 2 aminoethyl 2 methyl 1 h indol 5 ol".ti,ab OR "enteramine".ti,ab OR "5 ht".ti,ab OR "hydroxytryptamine".ti,ab OR "thromboxane a2".ti,ab OR "txa2".ti,ab OR "arachidonic acid".ti,ab OR "aa".ti,ab OR "acetylsalicylic acid".ti,ab OR "aspirin".ti,ab OR "platelet derived growth factor".ti,ab OR "pdgf receptor".ti,ab OR "pdgf".ti,ab OR "receptor tgf beta".ti,ab OR "tgfbeta".ti,ab OR "tgf beta".ti,ab OR "platelet transforming growth factor".ti,ab OR "gmp 140".ti,ab OR "p selectin".ti,ab OR "padgem".ti,ab OR "cd62p antigen".ti,ab OR "cd62p antigens".ti,ab OR "cd62p".ti,ab OR "alpha granule membrane protein".ti,ab OR "lecam3".ti,ab OR "gpvi".ti,ab OR "platelet membrane glycoprotein p62".ti,ab OR "glycoprotein gp vi".ti,ab OR "platelet glycoprotein vi".ti,ab OR "human glycoprotein vi".ti,ab OR "human alpha2beta1".ti,ab OR "integrin vla 2".ti,ab OR "vla 2".ti,ab OR "glycoprotein ia iia".ti,ab OR "alpha2beta1integrin".ti,ab OR "very late antigen 2".ti,ab OR "late antigen 2".ti,ab OR "cd49b cd29".ti,ab OR "platelet membrane glycoprotein ia/iia".ti,ab OR "platelet membrane glycoprotein ia iia".ti,ab OR "platelet membrane glycoprotein ia/iia complex".ti,ab OR "platelet membrane glycoprotein ia iia complex".ti,ab OR "platelet glycoprotein gpiib iiia complex".ti,ab OR "glycoproteins iib iiia".ti,ab OR "glycoproteins iib iiia complex".ti,ab OR "integrin alpha iib".ti,ab OR "integrin alpha iib beta 3".ti,ab OR "platelet glycoprotein gpib ix complex".ti,ab OR "platelet glycoprotein gpib ix complex".ti,ab OR "glycoprotein ib ix complex".ti,ab OR "platelet membrane glycoprotein ib".ti,ab OR "glycoprotein ib beta".ti,ab OR "cd42c".ti,ab OR "calcitonin gene related peptide".ti,ab OR "calcitonin gene related peptide ii".ti,ab OR "beta cgrp".ti,ab OR "beta calcitonin gene related peptide".ti,ab OR "alpha cgrp".ti,ab OR "alpha calcitonin gene related peptide".ti,ab OR "calcitonin gene related peptide i".ti,ab OR "cgrp".ti,ab OR "cyclo oxygenase i".ti,ab OR "cox1".ti,ab OR "prostaglandin h synthase 1".ti,ab OR "prostaglandin synthase cyclooxygenase".ti,ab OR "prostaglandin synthase".ti,ab OR "cox 1 synthesis".ti,ab OR "cox 1 prostaglandin".ti,ab OR "cyclooxygenase 1".ti,ab OR "endothelium derived vasoconstrictor factors".ti,ab OR "vasoconstrictor factors".ti,ab OR "endothelium derived endothelin 1".ti,ab OR "nitric nitrogen".ti,ab OR "endothelium derived nitric oxide".ti,ab OR "vasodilator nitrates".ti,ab OR "vasodilator nitric oxide".ti,ab OR "von willebrand factor cleaving protease".ti,ab OR "adamts13".ti,ab OR "vwf cleaving protease".ti,ab OR "vwf cleaving protease adamts13".ti,ab OR "p2y12 receptors".ti,ab OR "p2y12 receptor".ti,ab OR "p2y12".ti,ab OR "purinergic p2y12 receptors".ti,ab OR "purinergic p2y12 receptor".ti,ab OR "purinergic receptor p2y".ti,ab OR "p2y adp receptor".ti,ab OR "p2y adp receptors".ti,ab OR "p selectin glycoprotein ligand 1".ti,ab OR "p selectin".ti,ab OR CD162.ti,ab OR "platelet p selectin".ti,ab OR "psgl 1 protein".ti,ab OR "selectin P ligand protein".ti,ab OR "endocannabinoid".ti,ab OR "endocannabinoids".ti,ab OR "cb2 receptor".ti,ab OR "cannabinoid receptor cb2".ti,ab OR "cannabinoid receptor 2".ti,ab OR "cannabinoid receptor".ti,ab OR "cannabinoid cb1 receptor".ti,ab OR "cannabinoid cb2 receptor".ti,ab OR "cannabinoid receptor cb1".ti,ab OR "cannabinoid receptor 1".ti,ab OR "cb1 cannabinoid receptor".ti,ab OR "cb2 cannabinoid receptor".ti,ab OR "glyceryl 2 arachidonate".ti,ab OR "2 ag".ti,ab OR "aea".ti,ab OR "2 arachidonoylglycerol".ti,ab OR "anandamide".ti,ab OR "n arachidonoylethanolamine".ti,ab OR "metalloproteinase 9".ti,ab OR "matrix metalloproteinase 9".ti,ab OR "mmp9".ti,ab OR "metalloproteinase".ti,ab OR "metallopeptidases".ti,ab OR "metalloproteinases".ti,ab OR "metallopeptidase".ti,ab OR "type iv collagenase".ti,ab OR "type iv collagenase mmp 2".ti,ab OR "type iv collagenase mmp 9".ti,ab OR "metalloproteinase mmp 9".ti,ab OR "metalloproteinase 1".ti,ab OR "mmp1".ti,ab OR "metalloproteinase mmp1".ti,ab OR "mmp 1".ti,ab OR "mmp 9".ti,ab OR "matrix metalloproteinase 1".ti,ab OR "metalloproteinase 3".ti,ab OR "mmp3".ti,ab OR "matrix metalloproteinase 3".ti,ab OR "metalloproteinase 2".ti,ab OR "mmp2".ti,ab OR "matrix metalloproteinase 2".ti,ab OR "type iv collagenase mmp 2".ti,ab OR "mmp 2 gelatinase a".ti,ab) AND (*"Sex"/ OR exp *"Gender"/ OR *"gender and sex"/ OR "Gender".ti,ab OR "Sex".ti OR exp *"Sexual Characteristics"/ OR "Sex Characteristic*".ti,ab OR "Sex Difference*".ti,ab OR "Gender Difference*".ti,ab OR "Sex Based".ti,ab OR Sex Dimorphism*.ti,ab OR Sexual Dimorphism*.ti,ab OR (("Women".ti OR "Woman".ti OR "Female".ti) AND ("Men".ti OR "Man".ti OR "Male".ti)))) OR ((exp *"cerebrovascular accident"/ OR "stroke".ti OR "cva".ti OR "cerebrovascular accident".ti OR "cerebrovascular accidents".ti OR "cerebrovascular apoplexy".ti OR "apoplexy".ti OR "brain vascular accidents".ti OR "vascular accidents".ti OR "cerebrovascular stroke".ti OR "cerebrovascular strokes".ti OR "cerebrovascular apoplexy".ti OR "cerebral stroke".ti OR "cerebral strokes".ti OR "acute stroke".ti OR "acute strokes".ti OR "acute cerebrovascular accident".ti OR "acute cerebrovascular accidents".ti OR "cerebrovascular accidents".ti OR "brain infarction".ti OR "brain infarctions".ti OR "brain infarct".ti OR "brain infarcts".ti OR "anterior circulation infarction".ti OR "venous infarction".ti OR "venous infarctions".ti OR "posterior circulation infarction".ti OR "posterior circulation infarcts".ti OR "brain ischemia".ti OR "ischemic encephalopathy".ti OR "ischemic encephalopathies".ti OR "cerebral ischemia".ti OR "cerebral ischemias".ti) AND ("blood coagulation".ti OR "blood coagulation factor".ti OR "blood coagulation factors".ti OR "coagulation factor".ti OR "coagulation factors".ti OR "clotting factor".ti OR "clotting factors".ti OR "blood clotting".ti OR "blood clotting factor".ti OR "blood clotting factors".ti OR "thromboses".ti OR "thrombus".ti OR "blood clot".ti OR "blood clots".ti OR "thrombus formation".ti OR "hemostases".ti OR "platelet activation".ti OR "platelet activations".ti OR "platelet aggregation".ti OR "platelet clotting".ti OR "platelet adhesion".ti OR exp *"Blood Clotting Factor"/ OR "factor i".ti OR "blood coagulation factor i".ti OR "coagulation factor i".ti OR "fibrinogen".ti OR "gamma fibrinogen".ti OR "gamma fibrinogen".ti OR "fi".ti OR "factor ii".ti OR "blood coagulation factor ii".ti OR "differentiation reversal factor".ti OR "coagulation factor ii".ti OR "prothrombin".ti OR "fii".ti OR "thromboplastin".ti OR "coagulin".ti OR "factor iii".ti OR "fiii".ti OR "coagulation factor iii".ti OR "coagulation tissue factor".ti OR "tissue thromboplastin".ti OR "thromboplastin".ti OR "procoagulant".ti OR "tissue factor procoagulant".ti OR "coagulation factor iv".ti OR "factor iv".ti OR "calcium 40".ti OR "calcium".ti OR "fv".ti OR "factor five".ti OR "blood coagulation factor v".ti OR "coagulation factor v".ti OR "coagulation factor v".ti OR "factor v".ti OR "activated factor v".ti OR "coagulation factor va".ti OR "factor va".ti OR "factor v leiden".ti OR "factor five leiden".ti OR "factor seven".ti OR "coagulation factor vii".ti OR "factor vii".ti OR "blood coagulation factor vii".ti OR "proconvertin".ti OR "coagulation factor 7".ti OR "factor 7".ti OR "coagulation factor viia".ti OR "factor viia".ti OR "activated factor vii".ti OR "activated factor vii".ti OR "factor 7a".ti OR "factor 8".ti OR "coagulation factor viii".ti OR "thromboplastinogen".ti OR "blood coagulation factor viii".ti OR "factor eight".ti OR "hyatt c".ti OR "factor viiic".ti OR "f viii c".ti OR "factor viii".ti OR "blood coagulation factor viii".ti OR "coagulation factor viiia".ti OR "factor viiia".ti OR "coagulation factor viii".ti OR "thrombin activated factor viii".ti OR "factor 8a".ti OR "activated factor viii".ti OR "factor nine".ti OR "autoprothrombin ii".ti OR "christmas factor".ti OR "factor ix".ti OR "blood coagulation factor ix".ti OR "factor ix complex".ti OR "factor 9".ti OR "coagulation factor ix".ti OR "coagulation factor ixa".ti OR "factor ixa".ti OR "activated factor ix".ti OR "factor 9a".ti OR "plasma thromboplastin component".ti OR "factor ten".ti OR "autoprothrombin iii".ti OR "coagulation factor x".ti OR "factor x".ti OR "blood coagulation factor x".ti OR "stuart factor".ti OR "stuart prower factor".ti OR "factor 10".ti OR "activated factor x".ti OR "activated coagulation factor x".ti OR "factor xa".ti OR "blood coagulation factor x".ti OR "factor 10a".ti OR "autoprothrombin c".ti OR "factor eleven".ti OR "blood coagulation factor xi".ti OR "plasma thromboplastin".ti OR "plasma thromboplastin antecedent".ti OR "antecedent".ti OR "plasma thromboplastin".ti OR "thromboplastin antecedent".ti OR "coagulation factor xi".ti OR "factor xi".ti OR "coagulation factor 11".ti OR "factor 11".ti OR "activated factor xi".ti OR "coagulation factor xia".ti OR "factor xia".ti OR "blood coagulation factor xi".ti OR "factor 11a".ti OR "factor twelve".ti OR "hageman factor".ti OR "factor 12".ti OR "coagulation factor xii".ti OR "activated factor xii".ti OR "coagulation factor xiia".ti OR "factor xiia".ti OR "blood coagulation factor xii".ti OR "activated prekallikrein".ti OR "prekallikrein".ti OR "factor xii".ti OR "activated hageman factor".ti OR "hageman factor fragment".ti OR "factor thirteen".ti OR "coagulation factor xiii".ti OR "factor xiii".ti OR "factor xiii".ti OR "transamidase".ti OR "fibrinase".ti OR "laki lorand factor".ti OR "blood coagulation factor xiii".ti OR "factor 13".ti OR "fibrin stabilizing factor".ti OR "stabilizing factor".ti OR "activated factor xiii".ti OR "factor 13a".ti OR "plasma transglutaminase".ti OR "transglutaminase".ti OR "beta thromboglobulin".ti OR "beta 2 thromboglobulin".ti OR "thromboglobulin".ti OR "fibrinopeptides a".ti,ab OR "fibrinopeptides b".ti OR "kalliginogenase".ti OR "kallikrein".ti OR "kallikrein kininogenase".ti OR "callicrein".ti OR "kinin forming enzyme".ti OR "kinin forming".ti OR "kallikrein light chain".ti OR "kallikrein padutin".ti OR "kallikrein a".ti OR "beta kallikrein".ti OR "kallikrein b".ti OR "alpha kallikrein".ti OR "plasma kallikrein".ti OR "kininogenin".ti OR "kallikrein i".ti OR "plasma prokallikrein".ti OR "prokallikrein".ti OR "prokinins".ti OR "cystatins".ti OR "t kininogen".ti OR "low molecular weight kininogens".ti OR "lmwk".ti OR "high molecular weight kininogens".ti OR "hmwk".ti OR "fitzgerald factor".ti OR "plasminogen".ti OR "plasminogen activator".ti OR "plasminogen activator inhibitors".ti OR "endothelial plasminogen activator".ti OR "endothelial plasminogen activator inhibitor".ti OR "pai 1".ti OR "serpin e1".ti OR "serpine1".ti OR "serpine1 protein".ti OR "type 1 plasminogen activator inhibitor".ti OR "serpin b2".ti OR "type 2 plasminogen activator inhibitor".ti OR "pai 2".ti OR "pai 3".ti OR "plasminogen activator inhibitor".ti OR "activated protein c inhibitor".ti OR "plasminogen activator inhibitor 3".ti OR "phosphorylcholine".ti OR "acetyl glyceryl phosphorylcholine".ti OR "aggregating factor".ti OR "platelet aggregation enhancing factor".ti OR "agepc".ti OR "thrombocyte aggregating activity".ti OR "alkyl 2 acetyl sn glycerophosphocholine".ti OR "1 alkyl 2 acetyl sn glyceryl 3 phosphorylcholine".ti OR "1 alkyl 2 acetylglycerophosphocholine".ti OR "paf acether".ti OR "platelet activating substance".ti OR "platelet activating substances".ti OR "pf 3".ti OR "platelet factor 3".ti OR "chemokine cxcl4".ti OR "cxcl4".ti OR "chemokine pf4".ti OR "platelet factor 4".ti OR "heparin neutralizing protein".ti OR "antiheparin factor".ti OR "thrombase".ti OR "thrombostat".ti OR "alpha thrombin".ti OR "thrombin jmi".ti OR "jmi".ti OR "thrombin".ti OR "beta thrombin".ti OR "gamma thrombin".ti OR "plasminogen activator".ti OR "tissue activator d 44".ti OR "tisokinase".ti OR "tissue type plasminogen activator".ti OR "ttpa".ti OR "t plasminogen activator".ti OR "tissue type activator".ti OR "rt pa".ti OR "factor viiir ag".ti OR "factor viiir rco".ti OR "ristocetin willebrand factor".ti OR "von willebrand protein".ti OR "von willebrand factor".ti OR "factor viii related antigen".ti OR "ristocetin cofactor".ti OR "plasma factor viii complex".ti OR "vitamin k dependent protein s".ti OR "cofactor protein s".ti OR "protein s".ti OR "protein c".ti OR "antiphospholipid".ti,ab OR "glutamate".ti,ab OR "beta 2 glycoprotein".ti,ab OR "anti beta 2 glycoprotein".ti,ab OR "adp".ti OR "adenosine diphosphate".ti OR "adenosine pyrophosphate".ti OR "adenosine 5' pyrophosphate".ti OR "serotonin".ti OR "5 hydroxytryptamine".ti OR "hippophaine".ti OR "3 2 aminoethyl 2 methyl 1 h indol 5 ol".ti OR "enteramine".ti OR "5 ht".ti OR "hydroxytryptamine".ti OR "thromboxane a2".ti OR "txa2".ti OR "arachidonic acid".ti OR "aa".ti OR "acetylsalicylic acid".ti OR "aspirin".ti OR "platelet derived growth factor".ti OR "pdgf receptor".ti OR "pdgf".ti OR "receptor tgf beta".ti OR "tgfbeta".ti OR "tgf beta".ti OR "platelet transforming growth factor".ti OR "gmp 140".ti OR "p selectin".ti OR "padgem".ti OR "cd62p antigen".ti OR "cd62p antigens".ti OR "cd62p".ti OR "alpha granule membrane protein".ti OR "lecam3".ti OR "gpvi".ti OR "platelet membrane glycoprotein p62".ti OR "glycoprotein gp vi".ti OR "platelet glycoprotein vi".ti OR "human glycoprotein vi".ti OR "human alpha2beta1".ti OR "integrin vla 2".ti OR "vla 2".ti OR "glycoprotein ia iia".ti OR "alpha2beta1integrin".ti OR "very late antigen 2".ti OR "late antigen 2".ti OR "cd49b cd29".ti OR "platelet membrane glycoprotein ia/iia".ti OR "platelet membrane glycoprotein ia iia".ti OR "platelet membrane glycoprotein ia/iia complex".ti OR "platelet membrane glycoprotein ia iia complex".ti OR "platelet glycoprotein gpiib iiia complex".ti OR "glycoproteins iib iiia".ti OR "glycoproteins iib iiia complex".ti OR "integrin alpha iib".ti OR "integrin alpha iib beta 3".ti OR "platelet glycoprotein gpib ix complex".ti OR "platelet glycoprotein gpib ix complex".ti OR "glycoprotein ib ix complex".ti OR "platelet membrane glycoprotein ib".ti OR "glycoprotein ib beta".ti OR "cd42c".ti OR "calcitonin gene related peptide".ti OR "calcitonin gene related peptide ii".ti OR "beta cgrp".ti OR "beta calcitonin gene related peptide".ti OR "alpha cgrp".ti OR "alpha calcitonin gene related peptide".ti OR "calcitonin gene related peptide i".ti OR "cgrp".ti OR "cyclo oxygenase i".ti OR "cox1".ti OR "prostaglandin h synthase 1".ti OR "prostaglandin synthase cyclooxygenase".ti OR "prostaglandin synthase".ti OR "cox 1 synthesis".ti OR "cox 1 prostaglandin".ti OR "cyclooxygenase 1".ti OR "endothelium derived vasoconstrictor factors".ti OR "vasoconstrictor factors".ti OR "endothelium derived endothelin 1".ti OR "nitric nitrogen".ti OR "endothelium derived nitric oxide".ti OR "vasodilator nitrates".ti OR "vasodilator nitric oxide".ti OR "von willebrand factor cleaving protease".ti OR "adamts13".ti OR "vwf cleaving protease".ti OR "vwf cleaving protease adamts13".ti OR "p2y12 receptors".ti OR "p2y12 receptor".ti OR "p2y12".ti OR "purinergic p2y12 receptors".ti OR "purinergic p2y12 receptor".ti OR "purinergic receptor p2y".ti OR "p2y adp receptor".ti OR "p2y adp receptors".ti OR "p selectin glycoprotein ligand 1".ti OR "p selectin".ti OR CD162.ti OR "platelet p selectin".ti OR "psgl 1 protein".ti OR "selectin P ligand protein".ti OR "endocannabinoid".ti OR "endocannabinoids".ti OR "cb2 receptor".ti OR "cannabinoid receptor cb2".ti OR "cannabinoid receptor 2".ti OR "cannabinoid receptor".ti OR "cannabinoid cb1 receptor".ti OR "cannabinoid cb2 receptor".ti OR "cannabinoid receptor cb1".ti OR "cannabinoid receptor 1".ti OR "cb1 cannabinoid receptor".ti OR "cb2 cannabinoid receptor".ti OR "glyceryl 2 arachidonate".ti OR "2 ag".ti OR "aea".ti OR "2 arachidonoylglycerol".ti OR "anandamide".ti OR "n arachidonoylethanolamine".ti OR "metalloproteinase 9".ti OR "matrix metalloproteinase 9".ti OR "mmp9".ti OR "metalloproteinase".ti OR "metallopeptidases".ti OR "metalloproteinases".ti OR "metallopeptidase".ti OR "type iv collagenase".ti OR "type iv collagenase mmp 2".ti OR "type iv collagenase mmp 9".ti OR "metalloproteinase mmp 9".ti OR "metalloproteinase 1".ti OR "mmp1".ti OR "metalloproteinase mmp1".ti OR "mmp 1".ti OR "mmp 9".ti OR "matrix metalloproteinase 1".ti OR "metalloproteinase 3".ti OR "mmp3".ti OR "matrix metalloproteinase 3".ti OR "metalloproteinase 2".ti OR "mmp2".ti OR "matrix metalloproteinase 2".ti OR "type iv collagenase mmp 2".ti OR "mmp 2 gelatinase a".ti) AND (*"Sex"/ OR *"Gender"/ OR *"gender and sex"/ OR "Gender".ti,ab OR "Sex".ti,ab OR exp *"Sexual Characteristics"/ OR "Sex Characteristic*".ti,ab OR "Sex Difference*".ti,ab OR "Gender Difference*".ti,ab OR "Sex Based".ti,ab OR "Sex Dimorphism*".ti,ab OR "Sexual Dimorphism*".ti,ab OR ((*"Female"/ OR *"Girl"/ OR "Women".ti,ab OR "Woman".ti,ab OR "Female".ti,ab) AND (*"Male"/ OR *"Boy"/ OR "Men".ti,ab OR "Man".ti,ab OR "Male".ti,ab))))) NOT ("Tissue Plasminogen Activator"/pd OR "Aspirin"/pd OR exp "cerebrovascular accident"/th) NOT (("Case Report"/ OR "case report".ti OR exp "Review"/ OR "review".ti OR "systematic review"/ OR "systematic review".ti OR exp "meta analysis"/ OR "meta-analysis".ti) NOT ("Clinical Study"/ OR exp "Clinical Trial"/ OR "trial".ti OR "RCT".ti)) NOT (exp *"cerebrovascular accident"/th OR therap*.ti OR treat*.ti) AND (english.la OR dutch.la) NOT ((exp "Child"/ OR exp "Infant"/) NOT (exp "Adult"/)) NOT (conference abstract OR conference review).pt

**Web of Science**

((ti=("cerebrovascular accident" OR "stroke" OR "cva" OR "cerebrovascular accident" OR "cerebrovascular accidents" OR "cerebrovascular apoplexy" OR "apoplexy" OR "brain vascular accidents" OR "vascular accidents" OR "cerebrovascular stroke" OR "cerebrovascular strokes" OR "cerebrovascular apoplexy" OR "cerebral stroke" OR "cerebral strokes" OR "acute stroke" OR "acute strokes" OR "acute cerebrovascular accident" OR "acute cerebrovascular accidents" OR "cerebrovascular accidents" OR "brain infarction" OR "brain infarctions" OR "brain infarct" OR "brain infarcts" OR "anterior circulation infarction" OR "venous infarction" OR "venous infarctions" OR "posterior circulation infarction" OR "posterior circulation infarcts" OR "brain ischemia" OR "ischemic encephalopathy" OR "ischemic encephalopathies" OR "cerebral ischemia" OR "cerebral ischemias") AND ts=("blood coagulation" OR "blood coagulation factor" OR "blood coagulation factors" OR "coagulation factor" OR "coagulation factors" OR "clotting factor" OR "clotting factors" OR "blood clotting" OR "blood clotting factor" OR "blood clotting factors" OR "thromboses" OR "thrombus" OR "blood clot" OR "blood clots" OR "thrombus formation" OR "hemostases" OR "platelet activation" OR "platelet activations" OR "platelet aggregation" OR "platelet clotting" OR "platelet adhesion" OR "Blood Clotting Factor" OR "factor i" OR "blood coagulation factor i" OR "coagulation factor i" OR "fibrinogen" OR "gamma fibrinogen" OR "gamma fibrinogen" OR "fi" OR "factor ii" OR "blood coagulation factor ii" OR "differentiation reversal factor" OR "coagulation factor ii" OR "prothrombin" OR "fii" OR "thromboplastin" OR "coagulin" OR "factor iii" OR "fiii" OR "coagulation factor iii" OR "coagulation tissue factor" OR "tissue thromboplastin" OR "thromboplastin" OR "procoagulant" OR "tissue factor procoagulant" OR "coagulation factor iv" OR "factor iv" OR "calcium 40" OR "calcium" OR "fv" OR "factor five" OR "blood coagulation factor v" OR "coagulation factor v" OR "coagulation factor v" OR "factor v" OR "activated factor v" OR "coagulation factor va" OR "factor va" OR "factor v leiden" OR "factor five leiden" OR "factor seven" OR "coagulation factor vii" OR "factor vii" OR "blood coagulation factor vii" OR "proconvertin" OR "coagulation factor 7" OR "factor 7" OR "coagulation factor viia" OR "factor viia" OR "activated factor vii" OR "activated factor vii" OR "factor 7a" OR "factor 8" OR "coagulation factor viii" OR "thromboplastinogen" OR "blood coagulation factor viii" OR "factor eight" OR "hyatt c" OR "factor viiic" OR "f viii c" OR "factor viii" OR "blood coagulation factor viii" OR "coagulation factor viiia" OR "factor viiia" OR "coagulation factor viii" OR "thrombin activated factor viii" OR "factor 8a" OR "activated factor viii" OR "factor nine" OR "autoprothrombin ii" OR "christmas factor" OR "factor ix" OR "blood coagulation factor ix" OR "factor ix complex" OR "factor 9" OR "coagulation factor ix" OR "coagulation factor ixa" OR "factor ixa" OR "activated factor ix" OR "factor 9a" OR "plasma thromboplastin component" OR "factor ten" OR "autoprothrombin iii" OR "coagulation factor x" OR "factor x" OR "blood coagulation factor x" OR "stuart factor" OR "stuart prower factor" OR "factor 10" OR "activated factor x" OR "activated coagulation factor x" OR "factor xa" OR "blood coagulation factor x" OR "factor 10a" OR "autoprothrombin c" OR "factor eleven" OR "blood coagulation factor xi" OR "plasma thromboplastin" OR "plasma thromboplastin antecedent" OR "antecedent" OR "plasma thromboplastin" OR "thromboplastin antecedent" OR "coagulation factor xi" OR "factor xi" OR "coagulation factor 11" OR "factor 11" OR "activated factor xi" OR "coagulation factor xia" OR "factor xia" OR "blood coagulation factor xi" OR "factor 11a" OR "factor twelve" OR "hageman factor" OR "factor 12" OR "coagulation factor xii" OR "activated factor xii" OR "coagulation factor xiia" OR "factor xiia" OR "blood coagulation factor xii" OR "activated prekallikrein" OR "prekallikrein" OR "factor xii" OR "activated hageman factor" OR "hageman factor fragment" OR "factor thirteen" OR "coagulation factor xiii" OR "factor xiii" OR "factor xiii" OR "transamidase" OR "fibrinase" OR "laki lorand factor" OR "blood coagulation factor xiii" OR "factor 13" OR "fibrin stabilizing factor" OR "stabilizing factor" OR "activated factor xiii" OR "factor 13a" OR "plasma transglutaminase" OR "transglutaminase" OR "beta thromboglobulin" OR "beta 2 thromboglobulin" OR "thromboglobulin" OR "fibrinopeptides a" OR "fibrinopeptides b" OR "kalliginogenase" OR "kallikrein" OR "kallikrein kininogenase" OR "callicrein" OR "kinin forming enzyme" OR "kinin forming" OR "kallikrein light chain" OR "kallikrein padutin" OR "kallikrein a" OR "beta kallikrein" OR "kallikrein b" OR "alpha kallikrein" OR "plasma kallikrein" OR "kininogenin" OR "kallikrein i" OR "plasma prokallikrein" OR "prokallikrein" OR "prokinins" OR "cystatins" OR "t kininogen" OR "low molecular weight kininogens" OR "lmwk" OR "high molecular weight kininogens" OR "hmwk" OR "fitzgerald factor" OR "plasminogen" OR "plasminogen activator" OR "plasminogen activator inhibitors" OR "endothelial plasminogen activator" OR "endothelial plasminogen activator inhibitor" OR "pai 1" OR "serpin e1" OR "serpine1" OR "serpine1 protein" OR "type 1 plasminogen activator inhibitor" OR "serpin b2" OR "type 2 plasminogen activator inhibitor" OR "pai 2" OR "pai 3" OR "plasminogen activator inhibitor" OR "activated protein c inhibitor" OR "plasminogen activator inhibitor 3" OR "phosphorylcholine" OR "acetyl glyceryl phosphorylcholine" OR "aggregating factor" OR "platelet aggregation enhancing factor" OR "agepc" OR "thrombocyte aggregating activity" OR "alkyl 2 acetyl sn glycerophosphocholine" OR "1 alkyl 2 acetyl sn glyceryl 3 phosphorylcholine" OR "1 alkyl 2 acetylglycerophosphocholine" OR "paf acether" OR "platelet activating substance" OR "platelet activating substances" OR "pf 3" OR "platelet factor 3" OR "chemokine cxcl4" OR "cxcl4" OR "chemokine pf4" OR "platelet factor 4" OR "heparin neutralizing protein" OR "antiheparin factor" OR "thrombase" OR "thrombostat" OR "alpha thrombin" OR "thrombin jmi" OR "jmi" OR "thrombin" OR "beta thrombin" OR "gamma thrombin" OR "plasminogen activator" OR "tissue activator d 44" OR "tisokinase" OR "tissue type plasminogen activator" OR "ttpa" OR "t plasminogen activator" OR "tissue type activator" OR "rt pa" OR "factor viiir ag" OR "factor viiir rco" OR "ristocetin willebrand factor" OR "von willebrand protein" OR "von willebrand factor" OR "factor viii related antigen" OR "ristocetin cofactor" OR "plasma factor viii complex" OR "vitamin k dependent protein s" OR "cofactor protein s" OR "protein s" OR "protein c" OR "antiphospholipid" OR "glutamate" OR "beta 2 glycoprotein" OR "anti beta 2 glycoprotein" OR "adp" OR "adenosine diphosphate" OR "adenosine pyrophosphate" OR "adenosine 5' pyrophosphate" OR "serotonin" OR "5 hydroxytryptamine" OR "hippophaine" OR "3 2 aminoethyl 2 methyl 1 h indol 5 ol" OR "enteramine" OR "5 ht" OR "hydroxytryptamine" OR "thromboxane a2" OR "txa2" OR "arachidonic acid" OR "aa" OR "acetylsalicylic acid" OR "aspirin" OR "platelet derived growth factor" OR "pdgf receptor" OR "pdgf" OR "receptor tgf beta" OR "tgfbeta" OR "tgf beta" OR "platelet transforming growth factor" OR "gmp 140" OR "p selectin" OR "padgem" OR "cd62p antigen" OR "cd62p antigens" OR "cd62p" OR "alpha granule membrane protein" OR "lecam3" OR "gpvi" OR "platelet membrane glycoprotein p62" OR "glycoprotein gp vi" OR "platelet glycoprotein vi" OR "human glycoprotein vi" OR "human alpha2beta1" OR "integrin vla 2" OR "vla 2" OR "glycoprotein ia iia" OR "alpha2beta1integrin" OR "very late antigen 2" OR "late antigen 2" OR "cd49b cd29" OR "platelet membrane glycoprotein iaiia" OR "platelet membrane glycoprotein ia iia" OR "platelet membrane glycoprotein iaiia complex" OR "platelet membrane glycoprotein ia iia complex" OR "platelet glycoprotein gpiib iiia complex" OR "glycoproteins iib iiia" OR "glycoproteins iib iiia complex" OR "integrin alpha iib" OR "integrin alpha iib beta 3" OR "platelet glycoprotein gpib ix complex" OR "platelet glycoprotein gpib ix complex" OR "glycoprotein ib ix complex" OR "platelet membrane glycoprotein ib" OR "glycoprotein ib beta" OR "cd42c" OR "calcitonin gene related peptide" OR "calcitonin gene related peptide ii" OR "beta cgrp" OR "beta calcitonin gene related peptide" OR "alpha cgrp" OR "alpha calcitonin gene related peptide" OR "calcitonin gene related peptide i" OR "cgrp" OR "cyclo oxygenase i" OR "cox1" OR "prostaglandin h synthase 1" OR "prostaglandin synthase cyclooxygenase" OR "prostaglandin synthase" OR "cox 1 synthesis" OR "cox 1 prostaglandin" OR "cyclooxygenase 1" OR "endothelium derived vasoconstrictor factors" OR "vasoconstrictor factors" OR "endothelium derived endothelin 1" OR "nitric nitrogen" OR "endothelium derived nitric oxide" OR "vasodilator nitrates" OR "vasodilator nitric oxide" OR "von willebrand factor cleaving protease" OR "adamts13" OR "vwf cleaving protease" OR "vwf cleaving protease adamts13" OR "p2y12 receptors" OR "p2y12 receptor" OR "p2y12" OR "purinergic p2y12 receptors" OR "purinergic p2y12 receptor" OR "purinergic receptor p2y" OR "p2y adp receptor" OR "p2y adp receptors" OR "p selectin glycoprotein ligand 1" OR "p selectin" OR CD162 OR "platelet p selectin" OR "psgl 1 protein" OR "selectin P ligand protein" OR "endocannabinoid" OR "endocannabinoids" OR "cb2 receptor" OR "cannabinoid receptor cb2" OR "cannabinoid receptor 2" OR "cannabinoid receptor" OR "cannabinoid cb1 receptor" OR "cannabinoid cb2 receptor" OR "cannabinoid receptor cb1" OR "cannabinoid receptor 1" OR "cb1 cannabinoid receptor" OR "cb2 cannabinoid receptor" OR "glyceryl 2 arachidonate" OR "2 ag" OR "aea" OR "2 arachidonoylglycerol" OR "anandamide" OR "n arachidonoylethanolamine" OR "metalloproteinase 9" OR "matrix metalloproteinase 9" OR "mmp9" OR "metalloproteinase" OR "metallopeptidases" OR "metalloproteinases" OR "metallopeptidase" OR "type iv collagenase" OR "type iv collagenase mmp 2" OR "type iv collagenase mmp 9" OR "metalloproteinase mmp 9" OR "metalloproteinase 1" OR "mmp1" OR "metalloproteinase mmp1" OR "mmp 1" OR "mmp 9" OR "matrix metalloproteinase 1" OR "metalloproteinase 3" OR "mmp3" OR "matrix metalloproteinase 3" OR "metalloproteinase 2" OR "mmp2" OR "matrix metalloproteinase 2" OR "type iv collagenase mmp 2" OR "mmp 2 gelatinase a") AND ti=("Sex" OR "Gender" OR "gender and sex" OR "Gender" OR "Sex" OR "Sexual Characteristics" OR "Sex Characteristic*" OR "Sex Difference*" OR "Gender Difference*" OR "Sex Based" OR Sex Dimorphism* OR Sexual Dimorphism* OR (("Female" OR "Girl" OR "Women" OR "Woman" OR "Female") AND ("Male" OR "Boy" OR "Men" OR "Man" OR "Male")))) OR (ti=("cerebrovascular accident" OR "stroke" OR "cva" OR "cerebrovascular accident" OR "cerebrovascular accidents" OR "cerebrovascular apoplexy" OR "apoplexy" OR "brain vascular accidents" OR "vascular accidents" OR "cerebrovascular stroke" OR "cerebrovascular strokes" OR "cerebrovascular apoplexy" OR "cerebral stroke" OR "cerebral strokes" OR "acute stroke" OR "acute strokes" OR "acute cerebrovascular accident" OR "acute cerebrovascular accidents" OR "cerebrovascular accidents" OR "brain infarction" OR "brain infarctions" OR "brain infarct" OR "brain infarcts" OR "anterior circulation infarction" OR "venous infarction" OR "venous infarctions" OR "posterior circulation infarction" OR "posterior circulation infarcts" OR "brain ischemia" OR "ischemic encephalopathy" OR "ischemic encephalopathies" OR "cerebral ischemia" OR "cerebral ischemias") AND ti=("blood coagulation" OR "blood coagulation factor" OR "blood coagulation factors" OR "coagulation factor" OR "coagulation factors" OR "clotting factor" OR "clotting factors" OR "blood clotting" OR "blood clotting factor" OR "blood clotting factors" OR "thromboses" OR "thrombus" OR "blood clot" OR "blood clots" OR "thrombus formation" OR "hemostases" OR "platelet activation" OR "platelet activations" OR "platelet aggregation" OR "platelet clotting" OR "platelet adhesion" OR "Blood Clotting Factor" OR "factor i" OR "blood coagulation factor i" OR "coagulation factor i" OR "fibrinogen" OR "gamma fibrinogen" OR "gamma fibrinogen" OR "fi" OR "factor ii" OR "blood coagulation factor ii" OR "differentiation reversal factor" OR "coagulation factor ii" OR "prothrombin" OR "fii" OR "thromboplastin" OR "coagulin" OR "factor iii" OR "fiii" OR "coagulation factor iii" OR "coagulation tissue factor" OR "tissue thromboplastin" OR "thromboplastin" OR "procoagulant" OR "tissue factor procoagulant" OR "coagulation factor iv" OR "factor iv" OR "calcium 40" OR "calcium" OR "fv" OR "factor five" OR "blood coagulation factor v" OR "coagulation factor v" OR "coagulation factor v" OR "factor v" OR "activated factor v" OR "coagulation factor va" OR "factor va" OR "factor v leiden" OR "factor five leiden" OR "factor seven" OR "coagulation factor vii" OR "factor vii" OR "blood coagulation factor vii" OR "proconvertin" OR "coagulation factor 7" OR "factor 7" OR "coagulation factor viia" OR "factor viia" OR "activated factor vii" OR "activated factor vii" OR "factor 7a" OR "factor 8" OR "coagulation factor viii" OR "thromboplastinogen" OR "blood coagulation factor viii" OR "factor eight" OR "hyatt c" OR "factor viiic" OR "f viii c" OR "factor viii" OR "blood coagulation factor viii" OR "coagulation factor viiia" OR "factor viiia" OR "coagulation factor viii" OR "thrombin activated factor viii" OR "factor 8a" OR "activated factor viii" OR "factor nine" OR "autoprothrombin ii" OR "christmas factor" OR "factor ix" OR "blood coagulation factor ix" OR "factor ix complex" OR "factor 9" OR "coagulation factor ix" OR "coagulation factor ixa" OR "factor ixa" OR "activated factor ix" OR "factor 9a" OR "plasma thromboplastin component" OR "factor ten" OR "autoprothrombin iii" OR "coagulation factor x" OR "factor x" OR "blood coagulation factor x" OR "stuart factor" OR "stuart prower factor" OR "factor 10" OR "activated factor x" OR "activated coagulation factor x" OR "factor xa" OR "blood coagulation factor x" OR "factor 10a" OR "autoprothrombin c" OR "factor eleven" OR "blood coagulation factor xi" OR "plasma thromboplastin" OR "plasma thromboplastin antecedent" OR "antecedent" OR "plasma thromboplastin" OR "thromboplastin antecedent" OR "coagulation factor xi" OR "factor xi" OR "coagulation factor 11" OR "factor 11" OR "activated factor xi" OR "coagulation factor xia" OR "factor xia" OR "blood coagulation factor xi" OR "factor 11a" OR "factor twelve" OR "hageman factor" OR "factor 12" OR "coagulation factor xii" OR "activated factor xii" OR "coagulation factor xiia" OR "factor xiia" OR "blood coagulation factor xii" OR "activated prekallikrein" OR "prekallikrein" OR "factor xii" OR "activated hageman factor" OR "hageman factor fragment" OR "factor thirteen" OR "coagulation factor xiii" OR "factor xiii" OR "factor xiii" OR "transamidase" OR "fibrinase" OR "laki lorand factor" OR "blood coagulation factor xiii" OR "factor 13" OR "fibrin stabilizing factor" OR "stabilizing factor" OR "activated factor xiii" OR "factor 13a" OR "plasma transglutaminase" OR "transglutaminase" OR "beta thromboglobulin" OR "beta 2 thromboglobulin" OR "thromboglobulin" OR "fibrinopeptides a" OR "fibrinopeptides b" OR "kalliginogenase" OR "kallikrein" OR "kallikrein kininogenase" OR "callicrein" OR "kinin forming enzyme" OR "kinin forming" OR "kallikrein light chain" OR "kallikrein padutin" OR "kallikrein a" OR "beta kallikrein" OR "kallikrein b" OR "alpha kallikrein" OR "plasma kallikrein" OR "kininogenin" OR "kallikrein i" OR "plasma prokallikrein" OR "prokallikrein" OR "prokinins" OR "cystatins" OR "t kininogen" OR "low molecular weight kininogens" OR "lmwk" OR "high molecular weight kininogens" OR "hmwk" OR "fitzgerald factor" OR "plasminogen" OR "plasminogen activator" OR "plasminogen activator inhibitors" OR "endothelial plasminogen activator" OR "endothelial plasminogen activator inhibitor" OR "pai 1" OR "serpin e1" OR "serpine1" OR "serpine1 protein" OR "type 1 plasminogen activator inhibitor" OR "serpin b2" OR "type 2 plasminogen activator inhibitor" OR "pai 2" OR "pai 3" OR "plasminogen activator inhibitor" OR "activated protein c inhibitor" OR "plasminogen activator inhibitor 3" OR "phosphorylcholine" OR "acetyl glyceryl phosphorylcholine" OR "aggregating factor" OR "platelet aggregation enhancing factor" OR "agepc" OR "thrombocyte aggregating activity" OR "alkyl 2 acetyl sn glycerophosphocholine" OR "1 alkyl 2 acetyl sn glyceryl 3 phosphorylcholine" OR "1 alkyl 2 acetylglycerophosphocholine" OR "paf acether" OR "platelet activating substance" OR "platelet activating substances" OR "pf 3" OR "platelet factor 3" OR "chemokine cxcl4" OR "cxcl4" OR "chemokine pf4" OR "platelet factor 4" OR "heparin neutralizing protein" OR "antiheparin factor" OR "thrombase" OR "thrombostat" OR "alpha thrombin" OR "thrombin jmi" OR "jmi" OR "thrombin" OR "beta thrombin" OR "gamma thrombin" OR "plasminogen activator" OR "tissue activator d 44" OR "tisokinase" OR "tissue type plasminogen activator" OR "ttpa" OR "t plasminogen activator" OR "tissue type activator" OR "rt pa" OR "factor viiir ag" OR "factor viiir rco" OR "ristocetin willebrand factor" OR "von willebrand protein" OR "von willebrand factor" OR "factor viii related antigen" OR "ristocetin cofactor" OR "plasma factor viii complex" OR "vitamin k dependent protein s" OR "cofactor protein s" OR "protein s" OR "protein c" OR "antiphospholipid" OR "glutamate" OR "beta 2 glycoprotein" OR "anti beta 2 glycoprotein" OR "adp" OR "adenosine diphosphate" OR "adenosine pyrophosphate" OR "adenosine 5' pyrophosphate" OR "serotonin" OR "5 hydroxytryptamine" OR "hippophaine" OR "3 2 aminoethyl 2 methyl 1 h indol 5 ol" OR "enteramine" OR "5 ht" OR "hydroxytryptamine" OR "thromboxane a2" OR "txa2" OR "arachidonic acid" OR "aa" OR "acetylsalicylic acid" OR "aspirin" OR "platelet derived growth factor" OR "pdgf receptor" OR "pdgf" OR "receptor tgf beta" OR "tgfbeta" OR "tgf beta" OR "platelet transforming growth factor" OR "gmp 140" OR "p selectin" OR "padgem" OR "cd62p antigen" OR "cd62p antigens" OR "cd62p" OR "alpha granule membrane protein" OR "lecam3" OR "gpvi" OR "platelet membrane glycoprotein p62" OR "glycoprotein gp vi" OR "platelet glycoprotein vi" OR "human glycoprotein vi" OR "human alpha2beta1" OR "integrin vla 2" OR "vla 2" OR "glycoprotein ia iia" OR "alpha2beta1integrin" OR "very late antigen 2" OR "late antigen 2" OR "cd49b cd29" OR "platelet membrane glycoprotein iaiia" OR "platelet membrane glycoprotein ia iia" OR "platelet membrane glycoprotein iaiia complex" OR "platelet membrane glycoprotein ia iia complex" OR "platelet glycoprotein gpiib iiia complex" OR "glycoproteins iib iiia" OR "glycoproteins iib iiia complex" OR "integrin alpha iib" OR "integrin alpha iib beta 3" OR "platelet glycoprotein gpib ix complex" OR "platelet glycoprotein gpib ix complex" OR "glycoprotein ib ix complex" OR "platelet membrane glycoprotein ib" OR "glycoprotein ib beta" OR "cd42c" OR "calcitonin gene related peptide" OR "calcitonin gene related peptide ii" OR "beta cgrp" OR "beta calcitonin gene related peptide" OR "alpha cgrp" OR "alpha calcitonin gene related peptide" OR "calcitonin gene related peptide i" OR "cgrp" OR "cyclo oxygenase i" OR "cox1" OR "prostaglandin h synthase 1" OR "prostaglandin synthase cyclooxygenase" OR "prostaglandin synthase" OR "cox 1 synthesis" OR "cox 1 prostaglandin" OR "cyclooxygenase 1" OR "endothelium derived vasoconstrictor factors" OR "vasoconstrictor factors" OR "endothelium derived endothelin 1" OR "nitric nitrogen" OR "endothelium derived nitric oxide" OR "vasodilator nitrates" OR "vasodilator nitric oxide" OR "von willebrand factor cleaving protease" OR "adamts13" OR "vwf cleaving protease" OR "vwf cleaving protease adamts13" OR "p2y12 receptors" OR "p2y12 receptor" OR "p2y12" OR "purinergic p2y12 receptors" OR "purinergic p2y12 receptor" OR "purinergic receptor p2y" OR "p2y adp receptor" OR "p2y adp receptors" OR "p selectin glycoprotein ligand 1" OR "p selectin" OR CD162 OR "platelet p selectin" OR "psgl 1 protein" OR "selectin P ligand protein" OR "endocannabinoid" OR "endocannabinoids" OR "cb2 receptor" OR "cannabinoid receptor cb2" OR "cannabinoid receptor 2" OR "cannabinoid receptor" OR "cannabinoid cb1 receptor" OR "cannabinoid cb2 receptor" OR "cannabinoid receptor cb1" OR "cannabinoid receptor 1" OR "cb1 cannabinoid receptor" OR "cb2 cannabinoid receptor" OR "glyceryl 2 arachidonate" OR "2 ag" OR "aea" OR "2 arachidonoylglycerol" OR "anandamide" OR "n arachidonoylethanolamine" OR "metalloproteinase 9" OR "matrix metalloproteinase 9" OR "mmp9" OR "metalloproteinase" OR "metallopeptidases" OR "metalloproteinases" OR "metallopeptidase" OR "type iv collagenase" OR "type iv collagenase mmp 2" OR "type iv collagenase mmp 9" OR "metalloproteinase mmp 9" OR "metalloproteinase 1" OR "mmp1" OR "metalloproteinase mmp1" OR "mmp 1" OR "mmp 9" OR "matrix metalloproteinase 1" OR "metalloproteinase 3" OR "mmp3" OR "matrix metalloproteinase 3" OR "metalloproteinase 2" OR "mmp2" OR "matrix metalloproteinase 2" OR "type iv collagenase mmp 2" OR "mmp 2 gelatinase a") AND ts=("Sex" OR "Gender" OR "gender and sex" OR "Gender" OR "Sex" OR "Sexual Characteristics" OR "Sex Characteristic*" OR "Sex Difference*" OR "Gender Difference*" OR "Sex Based" OR "Sex Dimorphism*" OR "Sexual Dimorphism*" OR (("Female" OR "Girl" OR "Women" OR "Woman" OR "Female") AND ("Male" OR "Boy" OR "Men" OR "Man" OR "Male"))))) NOT ti=("Tissue Plasminogen Activator*" OR "Aspirin*" OR therap* OR treat*) NOT ti=(("Case Report" OR "case report" OR "Review" OR "review" OR "systematic review" OR "systematic review" OR "meta analysis" OR "meta-analysis") NOT ("Clinical Study" OR "Clinical Trial" OR "trial" OR "RCT")) AND la=(english OR dutch) NOT TI=(("Child*" OR "Infant*" OR pediat* OR paediat*) NOT ("Adult*")) NOT dt=(meeting abstract)

**Cochrane Library**

((("cerebrovascular accident" OR "stroke" OR "cva" OR "cerebrovascular accident" OR "cerebrovascular accidents" OR "cerebrovascular apoplexy" OR "apoplexy" OR "brain vascular accidents" OR "vascular accidents" OR "cerebrovascular stroke" OR "cerebrovascular strokes" OR "cerebrovascular apoplexy" OR "cerebral stroke" OR "cerebral strokes" OR "acute stroke" OR "acute strokes" OR "acute cerebrovascular accident" OR "acute cerebrovascular accidents" OR "cerebrovascular accidents" OR "brain infarction" OR "brain infarctions" OR "brain infarct" OR "brain infarcts" OR "anterior circulation infarction" OR "venous infarction" OR "venous infarctions" OR "posterior circulation infarction" OR "posterior circulation infarcts" OR "brain ischemia" OR "ischemic encephalopathy" OR "ischemic encephalopathies" OR "cerebral ischemia" OR "cerebral ischemias"):ti AND ("blood coagulation" OR "blood coagulation factor" OR "blood coagulation factors" OR "coagulation factor" OR "coagulation factors" OR "clotting factor" OR "clotting factors" OR "blood clotting" OR "blood clotting factor" OR "blood clotting factors" OR "thromboses" OR "thrombus" OR "blood clot" OR "blood clots" OR "thrombus formation" OR "hemostases" OR "platelet activation" OR "platelet activations" OR "platelet aggregation" OR "platelet clotting" OR "platelet adhesion" OR "Blood Clotting Factor" OR "factor i" OR "blood coagulation factor i" OR "coagulation factor i" OR "fibrinogen" OR "gamma fibrinogen" OR "gamma fibrinogen" OR "fi" OR "factor ii" OR "blood coagulation factor ii" OR "differentiation reversal factor" OR "coagulation factor ii" OR "prothrombin" OR "fii" OR "thromboplastin" OR "coagulin" OR "factor iii" OR "fiii" OR "coagulation factor iii" OR "coagulation tissue factor" OR "tissue thromboplastin" OR "thromboplastin" OR "procoagulant" OR "tissue factor procoagulant" OR "coagulation factor iv" OR "factor iv" OR "calcium 40" OR "calcium" OR "fv" OR "factor five" OR "blood coagulation factor v" OR "coagulation factor v" OR "coagulation factor v" OR "factor v" OR "activated factor v" OR "coagulation factor va" OR "factor va" OR "factor v leiden" OR "factor five leiden" OR "factor seven" OR "coagulation factor vii" OR "factor vii" OR "blood coagulation factor vii" OR "proconvertin" OR "coagulation factor 7" OR "factor 7" OR "coagulation factor viia" OR "factor viia" OR "activated factor vii" OR "activated factor vii" OR "factor 7a" OR "factor 8" OR "coagulation factor viii" OR "thromboplastinogen" OR "blood coagulation factor viii" OR "factor eight" OR "hyatt c" OR "factor viiic" OR "f viii c" OR "factor viii" OR "blood coagulation factor viii" OR "coagulation factor viiia" OR "factor viiia" OR "coagulation factor viii" OR "thrombin activated factor viii" OR "factor 8a" OR "activated factor viii" OR "factor nine" OR "autoprothrombin ii" OR "christmas factor" OR "factor ix" OR "blood coagulation factor ix" OR "factor ix complex" OR "factor 9" OR "coagulation factor ix" OR "coagulation factor ixa" OR "factor ixa" OR "activated factor ix" OR "factor 9a" OR "plasma thromboplastin component" OR "factor ten" OR "autoprothrombin iii" OR "coagulation factor x" OR "factor x" OR "blood coagulation factor x" OR "stuart factor" OR "stuart prower factor" OR "factor 10" OR "activated factor x" OR "activated coagulation factor x" OR "factor xa" OR "blood coagulation factor x" OR "factor 10a" OR "autoprothrombin c" OR "factor eleven" OR "blood coagulation factor xi" OR "plasma thromboplastin" OR "plasma thromboplastin antecedent" OR "antecedent" OR "plasma thromboplastin" OR "thromboplastin antecedent" OR "coagulation factor xi" OR "factor xi" OR "coagulation factor 11" OR "factor 11" OR "activated factor xi" OR "coagulation factor xia" OR "factor xia" OR "blood coagulation factor xi" OR "factor 11a" OR "factor twelve" OR "hageman factor" OR "factor 12" OR "coagulation factor xii" OR "activated factor xii" OR "coagulation factor xiia" OR "factor xiia" OR "blood coagulation factor xii" OR "activated prekallikrein" OR "prekallikrein" OR "factor xii" OR "activated hageman factor" OR "hageman factor fragment" OR "factor thirteen" OR "coagulation factor xiii" OR "factor xiii" OR "factor xiii" OR "transamidase" OR "fibrinase" OR "laki lorand factor" OR "blood coagulation factor xiii" OR "factor 13" OR "fibrin stabilizing factor" OR "stabilizing factor" OR "activated factor xiii" OR "factor 13a" OR "plasma transglutaminase" OR "transglutaminase" OR "beta thromboglobulin" OR "beta 2 thromboglobulin" OR "thromboglobulin" OR "fibrinopeptides a" OR "fibrinopeptides b" OR "kalliginogenase" OR "kallikrein" OR "kallikrein kininogenase" OR "callicrein" OR "kinin forming enzyme" OR "kinin forming" OR "kallikrein light chain" OR "kallikrein padutin" OR "kallikrein a" OR "beta kallikrein" OR "kallikrein b" OR "alpha kallikrein" OR "plasma kallikrein" OR "kininogenin" OR "kallikrein i" OR "plasma prokallikrein" OR "prokallikrein" OR "prokinins" OR "cystatins" OR "t kininogen" OR "low molecular weight kininogens" OR "lmwk" OR "high molecular weight kininogens" OR "hmwk" OR "fitzgerald factor" OR "plasminogen" OR "plasminogen activator" OR "plasminogen activator inhibitors" OR "endothelial plasminogen activator" OR "endothelial plasminogen activator inhibitor" OR "pai 1" OR "serpin e1" OR "serpine1" OR "serpine1 protein" OR "type 1 plasminogen activator inhibitor" OR "serpin b2" OR "type 2 plasminogen activator inhibitor" OR "pai 2" OR "pai 3" OR "plasminogen activator inhibitor" OR "activated protein c inhibitor" OR "plasminogen activator inhibitor 3" OR "phosphorylcholine" OR "acetyl glyceryl phosphorylcholine" OR "aggregating factor" OR "platelet aggregation enhancing factor" OR "agepc" OR "thrombocyte aggregating activity" OR "alkyl 2 acetyl sn glycerophosphocholine" OR "1 alkyl 2 acetyl sn glyceryl 3 phosphorylcholine" OR "1 alkyl 2 acetylglycerophosphocholine" OR "paf acether" OR "platelet activating substance" OR "platelet activating substances" OR "pf 3" OR "platelet factor 3" OR "chemokine cxcl4" OR "cxcl4" OR "chemokine pf4" OR "platelet factor 4" OR "heparin neutralizing protein" OR "antiheparin factor" OR "thrombase" OR "thrombostat" OR "alpha thrombin" OR "thrombin jmi" OR "jmi" OR "thrombin" OR "beta thrombin" OR "gamma thrombin" OR "plasminogen activator" OR "tissue activator d 44" OR "tisokinase" OR "tissue type plasminogen activator" OR "ttpa" OR "t plasminogen activator" OR "tissue type activator" OR "rt pa" OR "factor viiir ag" OR "factor viiir rco" OR "ristocetin willebrand factor" OR "von willebrand protein" OR "von willebrand factor" OR "factor viii related antigen" OR "ristocetin cofactor" OR "plasma factor viii complex" OR "vitamin k dependent protein s" OR "cofactor protein s" OR "protein s" OR "protein c" OR "antiphospholipid" OR "glutamate" OR "beta 2 glycoprotein" OR "anti beta 2 glycoprotein" OR "adp" OR "adenosine diphosphate" OR "adenosine pyrophosphate" OR "adenosine 5' pyrophosphate" OR "serotonin" OR "5 hydroxytryptamine" OR "hippophaine" OR "3 2 aminoethyl 2 methyl 1 h indol 5 ol" OR "enteramine" OR "5 ht" OR "hydroxytryptamine" OR "thromboxane a2" OR "txa2" OR "arachidonic acid" OR "aa" OR "acetylsalicylic acid" OR "aspirin" OR "platelet derived growth factor" OR "pdgf receptor" OR "pdgf" OR "receptor tgf beta" OR "tgfbeta" OR "tgf beta" OR "platelet transforming growth factor" OR "gmp 140" OR "p selectin" OR "padgem" OR "cd62p antigen" OR "cd62p antigens" OR "cd62p" OR "alpha granule membrane protein" OR "lecam3" OR "gpvi" OR "platelet membrane glycoprotein p62" OR "glycoprotein gp vi" OR "platelet glycoprotein vi" OR "human glycoprotein vi" OR "human alpha2beta1" OR "integrin vla 2" OR "vla 2" OR "glycoprotein ia iia" OR "alpha2beta1integrin" OR "very late antigen 2" OR "late antigen 2" OR "cd49b cd29" OR "platelet membrane glycoprotein iaiia" OR "platelet membrane glycoprotein ia iia" OR "platelet membrane glycoprotein iaiia complex" OR "platelet membrane glycoprotein ia iia complex" OR "platelet glycoprotein gpiib iiia complex" OR "glycoproteins iib iiia" OR "glycoproteins iib iiia complex" OR "integrin alpha iib" OR "integrin alpha iib beta 3" OR "platelet glycoprotein gpib ix complex" OR "platelet glycoprotein gpib ix complex" OR "glycoprotein ib ix complex" OR "platelet membrane glycoprotein ib" OR "glycoprotein ib beta" OR "cd42c" OR "calcitonin gene related peptide" OR "calcitonin gene related peptide ii" OR "beta cgrp" OR "beta calcitonin gene related peptide" OR "alpha cgrp" OR "alpha calcitonin gene related peptide" OR "calcitonin gene related peptide i" OR "cgrp" OR "cyclo oxygenase i" OR "cox1" OR "prostaglandin h synthase 1" OR "prostaglandin synthase cyclooxygenase" OR "prostaglandin synthase" OR "cox 1 synthesis" OR "cox 1 prostaglandin" OR "cyclooxygenase 1" OR "endothelium derived vasoconstrictor factors" OR "vasoconstrictor factors" OR "endothelium derived endothelin 1" OR "nitric nitrogen" OR "endothelium derived nitric oxide" OR "vasodilator nitrates" OR "vasodilator nitric oxide" OR "von willebrand factor cleaving protease" OR "adamts13" OR "vwf cleaving protease" OR "vwf cleaving protease adamts13" OR "p2y12 receptors" OR "p2y12 receptor" OR "p2y12" OR "purinergic p2y12 receptors" OR "purinergic p2y12 receptor" OR "purinergic receptor p2y" OR "p2y adp receptor" OR "p2y adp receptors" OR "p selectin glycoprotein ligand 1" OR "p selectin" OR CD162 OR "platelet p selectin" OR "psgl 1 protein" OR "selectin P ligand protein" OR "endocannabinoid" OR "endocannabinoids" OR "cb2 receptor" OR "cannabinoid receptor cb2" OR "cannabinoid receptor 2" OR "cannabinoid receptor" OR "cannabinoid cb1 receptor" OR "cannabinoid cb2 receptor" OR "cannabinoid receptor cb1" OR "cannabinoid receptor 1" OR "cb1 cannabinoid receptor" OR "cb2 cannabinoid receptor" OR "glyceryl 2 arachidonate" OR "2 ag" OR "aea" OR "2 arachidonoylglycerol" OR "anandamide" OR "n arachidonoylethanolamine" OR "metalloproteinase 9" OR "matrix metalloproteinase 9" OR "mmp9" OR "metalloproteinase" OR "metallopeptidases" OR "metalloproteinases" OR "metallopeptidase" OR "type iv collagenase" OR "type iv collagenase mmp 2" OR "type iv collagenase mmp 9" OR "metalloproteinase mmp 9" OR "metalloproteinase 1" OR "mmp1" OR "metalloproteinase mmp1" OR "mmp 1" OR "mmp 9" OR "matrix metalloproteinase 1" OR "metalloproteinase 3" OR "mmp3" OR "matrix metalloproteinase 3" OR "metalloproteinase 2" OR "mmp2" OR "matrix metalloproteinase 2" OR "type iv collagenase mmp 2" OR "mmp 2 gelatinase a"):ti,ab,kw AND ("Sex" OR "Gender" OR "gender and sex" OR "Gender" OR "Sex" OR "Sexual Characteristics" OR "Sex Characteristic*" OR "Sex Difference*" OR "Gender Difference*" OR "Sex Based" OR Sex Dimorphism* OR Sexual Dimorphism* OR (("Female" OR "Girl" OR "Women" OR "Woman" OR "Female") AND ("Male" OR "Boy" OR "Men" OR "Man" OR "Male"))):ti) OR (("cerebrovascular accident" OR "stroke" OR "cva" OR "cerebrovascular accident" OR "cerebrovascular accidents" OR "cerebrovascular apoplexy" OR "apoplexy" OR "brain vascular accidents" OR "vascular accidents" OR "cerebrovascular stroke" OR "cerebrovascular strokes" OR "cerebrovascular apoplexy" OR "cerebral stroke" OR "cerebral strokes" OR "acute stroke" OR "acute strokes" OR "acute cerebrovascular accident" OR "acute cerebrovascular accidents" OR "cerebrovascular accidents" OR "brain infarction" OR "brain infarctions" OR "brain infarct" OR "brain infarcts" OR "anterior circulation infarction" OR "venous infarction" OR "venous infarctions" OR "posterior circulation infarction" OR "posterior circulation infarcts" OR "brain ischemia" OR "ischemic encephalopathy" OR "ischemic encephalopathies" OR "cerebral ischemia" OR "cerebral ischemias"):ti AND ("blood coagulation" OR "blood coagulation factor" OR "blood coagulation factors" OR "coagulation factor" OR "coagulation factors" OR "clotting factor" OR "clotting factors" OR "blood clotting" OR "blood clotting factor" OR "blood clotting factors" OR "thromboses" OR "thrombus" OR "blood clot" OR "blood clots" OR "thrombus formation" OR "hemostases" OR "platelet activation" OR "platelet activations" OR "platelet aggregation" OR "platelet clotting" OR "platelet adhesion" OR "Blood Clotting Factor" OR "factor i" OR "blood coagulation factor i" OR "coagulation factor i" OR "fibrinogen" OR "gamma fibrinogen" OR "gamma fibrinogen" OR "fi" OR "factor ii" OR "blood coagulation factor ii" OR "differentiation reversal factor" OR "coagulation factor ii" OR "prothrombin" OR "fii" OR "thromboplastin" OR "coagulin" OR "factor iii" OR "fiii" OR "coagulation factor iii" OR "coagulation tissue factor" OR "tissue thromboplastin" OR "thromboplastin" OR "procoagulant" OR "tissue factor procoagulant" OR "coagulation factor iv" OR "factor iv" OR "calcium 40" OR "calcium" OR "fv" OR "factor five" OR "blood coagulation factor v" OR "coagulation factor v" OR "coagulation factor v" OR "factor v" OR "activated factor v" OR "coagulation factor va" OR "factor va" OR "factor v leiden" OR "factor five leiden" OR "factor seven" OR "coagulation factor vii" OR "factor vii" OR "blood coagulation factor vii" OR "proconvertin" OR "coagulation factor 7" OR "factor 7" OR "coagulation factor viia" OR "factor viia" OR "activated factor vii" OR "activated factor vii" OR "factor 7a" OR "factor 8" OR "coagulation factor viii" OR "thromboplastinogen" OR "blood coagulation factor viii" OR "factor eight" OR "hyatt c" OR "factor viiic" OR "f viii c" OR "factor viii" OR "blood coagulation factor viii" OR "coagulation factor viiia" OR "factor viiia" OR "coagulation factor viii" OR "thrombin activated factor viii" OR "factor 8a" OR "activated factor viii" OR "factor nine" OR "autoprothrombin ii" OR "christmas factor" OR "factor ix" OR "blood coagulation factor ix" OR "factor ix complex" OR "factor 9" OR "coagulation factor ix" OR "coagulation factor ixa" OR "factor ixa" OR "activated factor ix" OR "factor 9a" OR "plasma thromboplastin component" OR "factor ten" OR "autoprothrombin iii" OR "coagulation factor x" OR "factor x" OR "blood coagulation factor x" OR "stuart factor" OR "stuart prower factor" OR "factor 10" OR "activated factor x" OR "activated coagulation factor x" OR "factor xa" OR "blood coagulation factor x" OR "factor 10a" OR "autoprothrombin c" OR "factor eleven" OR "blood coagulation factor xi" OR "plasma thromboplastin" OR "plasma thromboplastin antecedent" OR "antecedent" OR "plasma thromboplastin" OR "thromboplastin antecedent" OR "coagulation factor xi" OR "factor xi" OR "coagulation factor 11" OR "factor 11" OR "activated factor xi" OR "coagulation factor xia" OR "factor xia" OR "blood coagulation factor xi" OR "factor 11a" OR "factor twelve" OR "hageman factor" OR "factor 12" OR "coagulation factor xii" OR "activated factor xii" OR "coagulation factor xiia" OR "factor xiia" OR "blood coagulation factor xii" OR "activated prekallikrein" OR "prekallikrein" OR "factor xii" OR "activated hageman factor" OR "hageman factor fragment" OR "factor thirteen" OR "coagulation factor xiii" OR "factor xiii" OR "factor xiii" OR "transamidase" OR "fibrinase" OR "laki lorand factor" OR "blood coagulation factor xiii" OR "factor 13" OR "fibrin stabilizing factor" OR "stabilizing factor" OR "activated factor xiii" OR "factor 13a" OR "plasma transglutaminase" OR "transglutaminase" OR "beta thromboglobulin" OR "beta 2 thromboglobulin" OR "thromboglobulin" OR "fibrinopeptides a" OR "fibrinopeptides b" OR "kalliginogenase" OR "kallikrein" OR "kallikrein kininogenase" OR "callicrein" OR "kinin forming enzyme" OR "kinin forming" OR "kallikrein light chain" OR "kallikrein padutin" OR "kallikrein a" OR "beta kallikrein" OR "kallikrein b" OR "alpha kallikrein" OR "plasma kallikrein" OR "kininogenin" OR "kallikrein i" OR "plasma prokallikrein" OR "prokallikrein" OR "prokinins" OR "cystatins" OR "t kininogen" OR "low molecular weight kininogens" OR "lmwk" OR "high molecular weight kininogens" OR "hmwk" OR "fitzgerald factor" OR "plasminogen" OR "plasminogen activator" OR "plasminogen activator inhibitors" OR "endothelial plasminogen activator" OR "endothelial plasminogen activator inhibitor" OR "pai 1" OR "serpin e1" OR "serpine1" OR "serpine1 protein" OR "type 1 plasminogen activator inhibitor" OR "serpin b2" OR "type 2 plasminogen activator inhibitor" OR "pai 2" OR "pai 3" OR "plasminogen activator inhibitor" OR "activated protein c inhibitor" OR "plasminogen activator inhibitor 3" OR "phosphorylcholine" OR "acetyl glyceryl phosphorylcholine" OR "aggregating factor" OR "platelet aggregation enhancing factor" OR "agepc" OR "thrombocyte aggregating activity" OR "alkyl 2 acetyl sn glycerophosphocholine" OR "1 alkyl 2 acetyl sn glyceryl 3 phosphorylcholine" OR "1 alkyl 2 acetylglycerophosphocholine" OR "paf acether" OR "platelet activating substance" OR "platelet activating substances" OR "pf 3" OR "platelet factor 3" OR "chemokine cxcl4" OR "cxcl4" OR "chemokine pf4" OR "platelet factor 4" OR "heparin neutralizing protein" OR "antiheparin factor" OR "thrombase" OR "thrombostat" OR "alpha thrombin" OR "thrombin jmi" OR "jmi" OR "thrombin" OR "beta thrombin" OR "gamma thrombin" OR "plasminogen activator" OR "tissue activator d 44" OR "tisokinase" OR "tissue type plasminogen activator" OR "ttpa" OR "t plasminogen activator" OR "tissue type activator" OR "rt pa" OR "factor viiir ag" OR "factor viiir rco" OR "ristocetin willebrand factor" OR "von willebrand protein" OR "von willebrand factor" OR "factor viii related antigen" OR "ristocetin cofactor" OR "plasma factor viii complex" OR "vitamin k dependent protein s" OR "cofactor protein s" OR "protein s" OR "protein c" OR "antiphospholipid" OR "glutamate" OR "beta 2 glycoprotein" OR "anti beta 2 glycoprotein" OR "adp" OR "adenosine diphosphate" OR "adenosine pyrophosphate" OR "adenosine 5' pyrophosphate" OR "serotonin" OR "5 hydroxytryptamine" OR "hippophaine" OR "3 2 aminoethyl 2 methyl 1 h indol 5 ol" OR "enteramine" OR "5 ht" OR "hydroxytryptamine" OR "thromboxane a2" OR "txa2" OR "arachidonic acid" OR "aa" OR "acetylsalicylic acid" OR "aspirin" OR "platelet derived growth factor" OR "pdgf receptor" OR "pdgf" OR "receptor tgf beta" OR "tgfbeta" OR "tgf beta" OR "platelet transforming growth factor" OR "gmp 140" OR "p selectin" OR "padgem" OR "cd62p antigen" OR "cd62p antigens" OR "cd62p" OR "alpha granule membrane protein" OR "lecam3" OR "gpvi" OR "platelet membrane glycoprotein p62" OR "glycoprotein gp vi" OR "platelet glycoprotein vi" OR "human glycoprotein vi" OR "human alpha2beta1" OR "integrin vla 2" OR "vla 2" OR "glycoprotein ia iia" OR "alpha2beta1integrin" OR "very late antigen 2" OR "late antigen 2" OR "cd49b cd29" OR "platelet membrane glycoprotein iaiia" OR "platelet membrane glycoprotein ia iia" OR "platelet membrane glycoprotein iaiia complex" OR "platelet membrane glycoprotein ia iia complex" OR "platelet glycoprotein gpiib iiia complex" OR "glycoproteins iib iiia" OR "glycoproteins iib iiia complex" OR "integrin alpha iib" OR "integrin alpha iib beta 3" OR "platelet glycoprotein gpib ix complex" OR "platelet glycoprotein gpib ix complex" OR "glycoprotein ib ix complex" OR "platelet membrane glycoprotein ib" OR "glycoprotein ib beta" OR "cd42c" OR "calcitonin gene related peptide" OR "calcitonin gene related peptide ii" OR "beta cgrp" OR "beta calcitonin gene related peptide" OR "alpha cgrp" OR "alpha calcitonin gene related peptide" OR "calcitonin gene related peptide i" OR "cgrp" OR "cyclo oxygenase i" OR "cox1" OR "prostaglandin h synthase 1" OR "prostaglandin synthase cyclooxygenase" OR "prostaglandin synthase" OR "cox 1 synthesis" OR "cox 1 prostaglandin" OR "cyclooxygenase 1" OR "endothelium derived vasoconstrictor factors" OR "vasoconstrictor factors" OR "endothelium derived endothelin 1" OR "nitric nitrogen" OR "endothelium derived nitric oxide" OR "vasodilator nitrates" OR "vasodilator nitric oxide" OR "von willebrand factor cleaving protease" OR "adamts13" OR "vwf cleaving protease" OR "vwf cleaving protease adamts13" OR "p2y12 receptors" OR "p2y12 receptor" OR "p2y12" OR "purinergic p2y12 receptors" OR "purinergic p2y12 receptor" OR "purinergic receptor p2y" OR "p2y adp receptor" OR "p2y adp receptors" OR "p selectin glycoprotein ligand 1" OR "p selectin" OR CD162 OR "platelet p selectin" OR "psgl 1 protein" OR "selectin P ligand protein" OR "endocannabinoid" OR "endocannabinoids" OR "cb2 receptor" OR "cannabinoid receptor cb2" OR "cannabinoid receptor 2" OR "cannabinoid receptor" OR "cannabinoid cb1 receptor" OR "cannabinoid cb2 receptor" OR "cannabinoid receptor cb1" OR "cannabinoid receptor 1" OR "cb1 cannabinoid receptor" OR "cb2 cannabinoid receptor" OR "glyceryl 2 arachidonate" OR "2 ag" OR "aea" OR "2 arachidonoylglycerol" OR "anandamide" OR "n arachidonoylethanolamine" OR "metalloproteinase 9" OR "matrix metalloproteinase 9" OR "mmp9" OR "metalloproteinase" OR "metallopeptidases" OR "metalloproteinases" OR "metallopeptidase" OR "type iv collagenase" OR "type iv collagenase mmp 2" OR "type iv collagenase mmp 9" OR "metalloproteinase mmp 9" OR "metalloproteinase 1" OR "mmp1" OR "metalloproteinase mmp1" OR "mmp 1" OR "mmp 9" OR "matrix metalloproteinase 1" OR "metalloproteinase 3" OR "mmp3" OR "matrix metalloproteinase 3" OR "metalloproteinase 2" OR "mmp2" OR "matrix metalloproteinase 2" OR "type iv collagenase mmp 2" OR "mmp 2 gelatinase a"):ti AND ("Sex" OR "Gender" OR "gender and sex" OR "Gender" OR "Sex" OR "Sexual Characteristics" OR "Sex Characteristic*" OR "Sex Difference*" OR "Gender Difference*" OR "Sex Based" OR "Sex Dimorphism*" OR "Sexual Dimorphism*" OR (("Female" OR "Girl" OR "Women" OR "Woman" OR "Female") AND ("Male" OR "Boy" OR "Men" OR "Man" OR "Male"))):ti,ab,kw)) NOT ("Tissue Plasminogen Activator*" OR "Aspirin*" OR therap* OR treat*):ti NOT (("Child*" OR "Infant*" OR pediat* OR paediat*) NOT "Adult*"):ti NOT (conference abstract):pt

**Emcare**

(((exp *"cerebrovascular accident"/ OR "stroke".ti OR "cva".ti OR "cerebrovascular accident".ti OR "cerebrovascular accidents".ti OR "cerebrovascular apoplexy".ti OR "apoplexy".ti OR "brain vascular accidents".ti OR "vascular accidents".ti OR "cerebrovascular stroke".ti OR "cerebrovascular strokes".ti OR "cerebrovascular apoplexy".ti OR "cerebral stroke".ti OR "cerebral strokes".ti OR "acute stroke".ti OR "acute strokes".ti OR "acute cerebrovascular accident".ti OR "acute cerebrovascular accidents".ti OR "cerebrovascular accidents".ti OR "brain infarction".ti OR "brain infarctions".ti OR "brain infarct".ti OR "brain infarcts".ti OR "anterior circulation infarction".ti OR "venous infarction".ti OR "venous infarctions".ti OR "posterior circulation infarction".ti OR "posterior circulation infarcts".ti OR "brain ischemia".ti OR "ischemic encephalopathy".ti OR "ischemic encephalopathies".ti OR "cerebral ischemia".ti OR "cerebral ischemias".ti) AND ("blood coagulation".ti,ab OR "blood coagulation factor".ti,ab OR "blood coagulation factors".ti,ab OR "coagulation factor".ti,ab OR "coagulation factors".ti,ab OR "clotting factor".ti,ab OR "clotting factors".ti,ab OR "blood clotting".ti,ab OR "blood clotting factor".ti,ab OR "blood clotting factors".ti,ab OR "thromboses".ti,ab OR "thrombus".ti,ab OR "blood clot".ti,ab OR "blood clots".ti,ab OR "thrombus formation".ti,ab OR "hemostases".ti,ab OR "platelet activation".ti,ab OR "platelet activations".ti,ab OR "platelet aggregation".ti,ab OR "platelet clotting".ti,ab OR "platelet adhesion".ti,ab OR exp *"Blood Clotting Factor"/ OR "factor i".ti,ab OR "blood coagulation factor i".ti,ab OR "coagulation factor i".ti,ab OR "fibrinogen".ti,ab OR "gamma fibrinogen".ti,ab OR "gamma fibrinogen".ti,ab OR "fi".ti,ab OR "factor ii".ti,ab OR "blood coagulation factor ii".ti,ab OR "differentiation reversal factor".ti,ab OR "coagulation factor ii".ti,ab OR "prothrombin".ti,ab OR "fii".ti,ab OR "thromboplastin".ti,ab OR "coagulin".ti,ab OR "factor iii".ti,ab OR "fiii".ti,ab OR "coagulation factor iii".ti,ab OR "coagulation tissue factor".ti,ab OR "tissue thromboplastin".ti,ab OR "thromboplastin".ti,ab OR "procoagulant".ti,ab OR "tissue factor procoagulant".ti,ab OR "coagulation factor iv".ti,ab OR "factor iv".ti,ab OR "calcium 40".ti,ab OR "calcium".ti,ab OR "fv".ti,ab OR "factor five".ti,ab OR "blood coagulation factor v".ti,ab OR "coagulation factor v".ti,ab OR "coagulation factor v".ti,ab OR "factor v".ti,ab OR "activated factor v".ti,ab OR "coagulation factor va".ti,ab OR "factor va".ti,ab OR "factor v leiden".ti,ab OR "factor five leiden".ti,ab OR "factor seven".ti,ab OR "coagulation factor vii".ti,ab OR "factor vii".ti,ab OR "blood coagulation factor vii".ti,ab OR "proconvertin".ti,ab OR "coagulation factor 7".ti,ab OR "factor 7".ti,ab OR "coagulation factor viia".ti,ab OR "factor viia".ti,ab OR "activated factor vii".ti,ab OR "activated factor vii".ti,ab OR "factor 7a".ti,ab OR "factor 8".ti,ab OR "coagulation factor viii".ti,ab OR "thromboplastinogen".ti,ab OR "blood coagulation factor viii".ti,ab OR "factor eight".ti,ab OR "hyatt c".ti,ab OR "factor viiic".ti,ab OR "f viii c".ti,ab OR "factor viii".ti,ab OR "blood coagulation factor viii".ti,ab OR "coagulation factor viiia".ti,ab OR "factor viiia".ti,ab OR "coagulation factor viii".ti,ab OR "thrombin activated factor viii".ti,ab OR "factor 8a".ti,ab OR "activated factor viii".ti,ab OR "factor nine".ti,ab OR "autoprothrombin ii".ti,ab OR "christmas factor".ti,ab OR "factor ix".ti,ab OR "blood coagulation factor ix".ti,ab OR "factor ix complex".ti,ab OR "factor 9".ti,ab OR "coagulation factor ix".ti,ab OR "coagulation factor ixa".ti,ab OR "factor ixa".ti,ab OR "activated factor ix".ti,ab OR "factor 9a".ti,ab OR "plasma thromboplastin component".ti,ab OR "factor ten".ti,ab OR "autoprothrombin iii".ti,ab OR "coagulation factor x".ti,ab OR "factor x".ti,ab OR "blood coagulation factor x".ti,ab OR "stuart factor".ti,ab OR "stuart prower factor".ti,ab OR "factor 10".ti,ab OR "activated factor x".ti,ab OR "activated coagulation factor x".ti,ab OR "factor xa".ti,ab OR "blood coagulation factor x".ti,ab OR "factor 10a".ti,ab OR "autoprothrombin c".ti,ab OR "factor eleven".ti,ab OR "blood coagulation factor xi".ti,ab OR "plasma thromboplastin".ti,ab OR "plasma thromboplastin antecedent".ti,ab OR "antecedent".ti,ab OR "plasma thromboplastin".ti,ab OR "thromboplastin antecedent".ti,ab OR "coagulation factor xi".ti,ab OR "factor xi".ti,ab OR "coagulation factor 11".ti,ab OR "factor 11".ti,ab OR "activated factor xi".ti,ab OR "coagulation factor xia".ti,ab OR "factor xia".ti,ab OR "blood coagulation factor xi".ti,ab OR "factor 11a".ti,ab OR "factor twelve".ti,ab OR "hageman factor".ti,ab OR "factor 12".ti,ab OR "coagulation factor xii".ti,ab OR "activated factor xii".ti,ab OR "coagulation factor xiia".ti,ab OR "factor xiia".ti,ab OR "blood coagulation factor xii".ti,ab OR "activated prekallikrein".ti,ab OR "prekallikrein".ti,ab OR "factor xii".ti,ab OR "activated hageman factor".ti,ab OR "hageman factor fragment".ti,ab OR "factor thirteen".ti,ab OR "coagulation factor xiii".ti,ab OR "factor xiii".ti,ab OR "factor xiii".ti,ab OR "transamidase".ti,ab OR "fibrinase".ti,ab OR "laki lorand factor".ti,ab OR "blood coagulation factor xiii".ti,ab OR "factor 13".ti,ab OR "fibrin stabilizing factor".ti,ab OR "stabilizing factor".ti,ab OR "activated factor xiii".ti,ab OR "factor 13a".ti,ab OR "plasma transglutaminase".ti,ab OR "transglutaminase".ti,ab OR "beta thromboglobulin".ti,ab OR "beta 2 thromboglobulin".ti,ab OR "thromboglobulin".ti,ab OR "fibrinopeptides a".ti,ab OR "fibrinopeptides b".ti,ab OR "kalliginogenase".ti,ab OR "kallikrein".ti,ab OR "kallikrein kininogenase".ti,ab OR "callicrein".ti,ab OR "kinin forming enzyme".ti,ab OR "kinin forming".ti,ab OR "kallikrein light chain".ti,ab OR "kallikrein padutin".ti,ab OR "kallikrein a".ti,ab OR "beta kallikrein".ti,ab OR "kallikrein b".ti,ab OR "alpha kallikrein".ti,ab OR "plasma kallikrein".ti,ab OR "kininogenin".ti,ab OR "kallikrein i".ti,ab OR "plasma prokallikrein".ti,ab OR "prokallikrein".ti,ab OR "prokinins".ti,ab OR "cystatins".ti,ab OR "t kininogen".ti,ab OR "low molecular weight kininogens".ti,ab OR "lmwk".ti,ab OR "high molecular weight kininogens".ti,ab OR "hmwk".ti,ab OR "fitzgerald factor".ti,ab OR "plasminogen".ti,ab OR "plasminogen activator".ti,ab OR "plasminogen activator inhibitors".ti,ab OR "endothelial plasminogen activator".ti,ab OR "endothelial plasminogen activator inhibitor".ti,ab OR "pai 1".ti,ab OR "serpin e1".ti,ab OR "serpine1".ti,ab OR "serpine1 protein".ti,ab OR "type 1 plasminogen activator inhibitor".ti,ab OR "serpin b2".ti,ab OR "type 2 plasminogen activator inhibitor".ti,ab OR "pai 2".ti,ab OR "pai 3".ti,ab OR "plasminogen activator inhibitor".ti,ab OR "activated protein c inhibitor".ti,ab OR "plasminogen activator inhibitor 3".ti,ab OR "phosphorylcholine".ti,ab OR "acetyl glyceryl phosphorylcholine".ti,ab OR "aggregating factor".ti,ab OR "platelet aggregation enhancing factor".ti,ab OR "agepc".ti,ab OR "thrombocyte aggregating activity".ti,ab OR "alkyl 2 acetyl sn glycerophosphocholine".ti,ab OR "1 alkyl 2 acetyl sn glyceryl 3 phosphorylcholine".ti,ab OR "1 alkyl 2 acetylglycerophosphocholine".ti,ab OR "paf acether".ti,ab OR "platelet activating substance".ti,ab OR "platelet activating substances".ti,ab OR "pf 3".ti,ab OR "platelet factor 3".ti,ab OR "chemokine cxcl4".ti,ab OR "cxcl4".ti,ab OR "chemokine pf4".ti,ab OR "platelet factor 4".ti,ab OR "heparin neutralizing protein".ti,ab OR "antiheparin factor".ti,ab OR "thrombase".ti,ab OR "thrombostat".ti,ab OR "alpha thrombin".ti,ab OR "thrombin jmi".ti,ab OR "jmi".ti,ab OR "thrombin".ti,ab OR "beta thrombin".ti,ab OR "gamma thrombin".ti,ab OR "plasminogen activator".ti,ab OR "tissue activator d 44".ti,ab OR "tisokinase".ti,ab OR "tissue type plasminogen activator".ti,ab OR "ttpa".ti,ab OR "t plasminogen activator".ti,ab OR "tissue type activator".ti,ab OR "rt pa".ti,ab OR "factor viiir ag".ti,ab OR "factor viiir rco".ti,ab OR "ristocetin willebrand factor".ti,ab OR "von willebrand protein".ti,ab OR "von willebrand factor".ti,ab OR "factor viii related antigen".ti,ab OR "ristocetin cofactor".ti,ab OR "plasma factor viii complex".ti,ab OR "vitamin k dependent protein s".ti,ab OR "cofactor protein s".ti,ab OR "protein s".ti,ab OR "protein c".ti,ab OR "antiphospholipid".ti,ab OR "glutamate".ti,ab OR "beta 2 glycoprotein".ti,ab OR "anti beta 2 glycoprotein".ti,ab OR "adp".ti,ab OR "adenosine diphosphate".ti,ab OR "adenosine pyrophosphate".ti,ab OR "adenosine 5' pyrophosphate".ti,ab OR "serotonin".ti,ab OR "5 hydroxytryptamine".ti,ab OR "hippophaine".ti,ab OR "3 2 aminoethyl 2 methyl 1 h indol 5 ol".ti,ab OR "enteramine".ti,ab OR "5 ht".ti,ab OR "hydroxytryptamine".ti,ab OR "thromboxane a2".ti,ab OR "txa2".ti,ab OR "arachidonic acid".ti,ab OR "aa".ti,ab OR "acetylsalicylic acid".ti,ab OR "aspirin".ti,ab OR "platelet derived growth factor".ti,ab OR "pdgf receptor".ti,ab OR "pdgf".ti,ab OR "receptor tgf beta".ti,ab OR "tgfbeta".ti,ab OR "tgf beta".ti,ab OR "platelet transforming growth factor".ti,ab OR "gmp 140".ti,ab OR "p selectin".ti,ab OR "padgem".ti,ab OR "cd62p antigen".ti,ab OR "cd62p antigens".ti,ab OR "cd62p".ti,ab OR "alpha granule membrane protein".ti,ab OR "lecam3".ti,ab OR "gpvi".ti,ab OR "platelet membrane glycoprotein p62".ti,ab OR "glycoprotein gp vi".ti,ab OR "platelet glycoprotein vi".ti,ab OR "human glycoprotein vi".ti,ab OR "human alpha2beta1".ti,ab OR "integrin vla 2".ti,ab OR "vla 2".ti,ab OR "glycoprotein ia iia".ti,ab OR "alpha2beta1integrin".ti,ab OR "very late antigen 2".ti,ab OR "late antigen 2".ti,ab OR "cd49b cd29".ti,ab OR "platelet membrane glycoprotein ia/iia".ti,ab OR "platelet membrane glycoprotein ia iia".ti,ab OR "platelet membrane glycoprotein ia/iia complex".ti,ab OR "platelet membrane glycoprotein ia iia complex".ti,ab OR "platelet glycoprotein gpiib iiia complex".ti,ab OR "glycoproteins iib iiia".ti,ab OR "glycoproteins iib iiia complex".ti,ab OR "integrin alpha iib".ti,ab OR "integrin alpha iib beta 3".ti,ab OR "platelet glycoprotein gpib ix complex".ti,ab OR "platelet glycoprotein gpib ix complex".ti,ab OR "glycoprotein ib ix complex".ti,ab OR "platelet membrane glycoprotein ib".ti,ab OR "glycoprotein ib beta".ti,ab OR "cd42c".ti,ab OR "calcitonin gene related peptide".ti,ab OR "calcitonin gene related peptide ii".ti,ab OR "beta cgrp".ti,ab OR "beta calcitonin gene related peptide".ti,ab OR "alpha cgrp".ti,ab OR "alpha calcitonin gene related peptide".ti,ab OR "calcitonin gene related peptide i".ti,ab OR "cgrp".ti,ab OR "cyclo oxygenase i".ti,ab OR "cox1".ti,ab OR "prostaglandin h synthase 1".ti,ab OR "prostaglandin synthase cyclooxygenase".ti,ab OR "prostaglandin synthase".ti,ab OR "cox 1 synthesis".ti,ab OR "cox 1 prostaglandin".ti,ab OR "cyclooxygenase 1".ti,ab OR "endothelium derived vasoconstrictor factors".ti,ab OR "vasoconstrictor factors".ti,ab OR "endothelium derived endothelin 1".ti,ab OR "nitric nitrogen".ti,ab OR "endothelium derived nitric oxide".ti,ab OR "vasodilator nitrates".ti,ab OR "vasodilator nitric oxide".ti,ab OR "von willebrand factor cleaving protease".ti,ab OR "adamts13".ti,ab OR "vwf cleaving protease".ti,ab OR "vwf cleaving protease adamts13".ti,ab OR "p2y12 receptors".ti,ab OR "p2y12 receptor".ti,ab OR "p2y12".ti,ab OR "purinergic p2y12 receptors".ti,ab OR "purinergic p2y12 receptor".ti,ab OR "purinergic receptor p2y".ti,ab OR "p2y adp receptor".ti,ab OR "p2y adp receptors".ti,ab OR "p selectin glycoprotein ligand 1".ti,ab OR "p selectin".ti,ab OR CD162.ti,ab OR "platelet p selectin".ti,ab OR "psgl 1 protein".ti,ab OR "selectin P ligand protein".ti,ab OR "endocannabinoid".ti,ab OR "endocannabinoids".ti,ab OR "cb2 receptor".ti,ab OR "cannabinoid receptor cb2".ti,ab OR "cannabinoid receptor 2".ti,ab OR "cannabinoid receptor".ti,ab OR "cannabinoid cb1 receptor".ti,ab OR "cannabinoid cb2 receptor".ti,ab OR "cannabinoid receptor cb1".ti,ab OR "cannabinoid receptor 1".ti,ab OR "cb1 cannabinoid receptor".ti,ab OR "cb2 cannabinoid receptor".ti,ab OR "glyceryl 2 arachidonate".ti,ab OR "2 ag".ti,ab OR "aea".ti,ab OR "2 arachidonoylglycerol".ti,ab OR "anandamide".ti,ab OR "n arachidonoylethanolamine".ti,ab OR "metalloproteinase 9".ti,ab OR "matrix metalloproteinase 9".ti,ab OR "mmp9".ti,ab OR "metalloproteinase".ti,ab OR "metallopeptidases".ti,ab OR "metalloproteinases".ti,ab OR "metallopeptidase".ti,ab OR "type iv collagenase".ti,ab OR "type iv collagenase mmp 2".ti,ab OR "type iv collagenase mmp 9".ti,ab OR "metalloproteinase mmp 9".ti,ab OR "metalloproteinase 1".ti,ab OR "mmp1".ti,ab OR "metalloproteinase mmp1".ti,ab OR "mmp 1".ti,ab OR "mmp 9".ti,ab OR "matrix metalloproteinase 1".ti,ab OR "metalloproteinase 3".ti,ab OR "mmp3".ti,ab OR "matrix metalloproteinase 3".ti,ab OR "metalloproteinase 2".ti,ab OR "mmp2".ti,ab OR "matrix metalloproteinase 2".ti,ab OR "type iv collagenase mmp 2".ti,ab OR "mmp 2 gelatinase a".ti,ab) AND (*"Sex"/ OR exp *"Gender"/ OR *"gender and sex"/ OR "Gender".ti,ab OR "Sex".ti OR exp *"Sexual Characteristics"/ OR "Sex Characteristic*".ti,ab OR "Sex Difference*".ti,ab OR "Gender Difference*".ti,ab OR "Sex Based".ti,ab OR Sex Dimorphism*.ti,ab OR Sexual Dimorphism*.ti,ab OR (("Women".ti OR "Woman".ti OR "Female".ti) AND ("Men".ti OR "Man".ti OR "Male".ti)))) OR ((exp *"cerebrovascular accident"/ OR "stroke".ti OR "cva".ti OR "cerebrovascular accident".ti OR "cerebrovascular accidents".ti OR "cerebrovascular apoplexy".ti OR "apoplexy".ti OR "brain vascular accidents".ti OR "vascular accidents".ti OR "cerebrovascular stroke".ti OR "cerebrovascular strokes".ti OR "cerebrovascular apoplexy".ti OR "cerebral stroke".ti OR "cerebral strokes".ti OR "acute stroke".ti OR "acute strokes".ti OR "acute cerebrovascular accident".ti OR "acute cerebrovascular accidents".ti OR "cerebrovascular accidents".ti OR "brain infarction".ti OR "brain infarctions".ti OR "brain infarct".ti OR "brain infarcts".ti OR "anterior circulation infarction".ti OR "venous infarction".ti OR "venous infarctions".ti OR "posterior circulation infarction".ti OR "posterior circulation infarcts".ti OR "brain ischemia".ti OR "ischemic encephalopathy".ti OR "ischemic encephalopathies".ti OR "cerebral ischemia".ti OR "cerebral ischemias".ti) AND ("blood coagulation".ti OR "blood coagulation factor".ti OR "blood coagulation factors".ti OR "coagulation factor".ti OR "coagulation factors".ti OR "clotting factor".ti OR "clotting factors".ti OR "blood clotting".ti OR "blood clotting factor".ti OR "blood clotting factors".ti OR "thromboses".ti OR "thrombus".ti OR "blood clot".ti OR "blood clots".ti OR "thrombus formation".ti OR "hemostases".ti OR "platelet activation".ti OR "platelet activations".ti OR "platelet aggregation".ti OR "platelet clotting".ti OR "platelet adhesion".ti OR exp *"Blood Clotting Factor"/ OR "factor i".ti OR "blood coagulation factor i".ti OR "coagulation factor i".ti OR "fibrinogen".ti OR "gamma fibrinogen".ti OR "gamma fibrinogen".ti OR "fi".ti OR "factor ii".ti OR "blood coagulation factor ii".ti OR "differentiation reversal factor".ti OR "coagulation factor ii".ti OR "prothrombin".ti OR "fii".ti OR "thromboplastin".ti OR "coagulin".ti OR "factor iii".ti OR "fiii".ti OR "coagulation factor iii".ti OR "coagulation tissue factor".ti OR "tissue thromboplastin".ti OR "thromboplastin".ti OR "procoagulant".ti OR "tissue factor procoagulant".ti OR "coagulation factor iv".ti OR "factor iv".ti OR "calcium 40".ti OR "calcium".ti OR "fv".ti OR "factor five".ti OR "blood coagulation factor v".ti OR "coagulation factor v".ti OR "coagulation factor v".ti OR "factor v".ti OR "activated factor v".ti OR "coagulation factor va".ti OR "factor va".ti OR "factor v leiden".ti OR "factor five leiden".ti OR "factor seven".ti OR "coagulation factor vii".ti OR "factor vii".ti OR "blood coagulation factor vii".ti OR "proconvertin".ti OR "coagulation factor 7".ti OR "factor 7".ti OR "coagulation factor viia".ti OR "factor viia".ti OR "activated factor vii".ti OR "activated factor vii".ti OR "factor 7a".ti OR "factor 8".ti OR "coagulation factor viii".ti OR "thromboplastinogen".ti OR "blood coagulation factor viii".ti OR "factor eight".ti OR "hyatt c".ti OR "factor viiic".ti OR "f viii c".ti OR "factor viii".ti OR "blood coagulation factor viii".ti OR "coagulation factor viiia".ti OR "factor viiia".ti OR "coagulation factor viii".ti OR "thrombin activated factor viii".ti OR "factor 8a".ti OR "activated factor viii".ti OR "factor nine".ti OR "autoprothrombin ii".ti OR "christmas factor".ti OR "factor ix".ti OR "blood coagulation factor ix".ti OR "factor ix complex".ti OR "factor 9".ti OR "coagulation factor ix".ti OR "coagulation factor ixa".ti OR "factor ixa".ti OR "activated factor ix".ti OR "factor 9a".ti OR "plasma thromboplastin component".ti OR "factor ten".ti OR "autoprothrombin iii".ti OR "coagulation factor x".ti OR "factor x".ti OR "blood coagulation factor x".ti OR "stuart factor".ti OR "stuart prower factor".ti OR "factor 10".ti OR "activated factor x".ti OR "activated coagulation factor x".ti OR "factor xa".ti OR "blood coagulation factor x".ti OR "factor 10a".ti OR "autoprothrombin c".ti OR "factor eleven".ti OR "blood coagulation factor xi".ti OR "plasma thromboplastin".ti OR "plasma thromboplastin antecedent".ti OR "antecedent".ti OR "plasma thromboplastin".ti OR "thromboplastin antecedent".ti OR "coagulation factor xi".ti OR "factor xi".ti OR "coagulation factor 11".ti OR "factor 11".ti OR "activated factor xi".ti OR "coagulation factor xia".ti OR "factor xia".ti OR "blood coagulation factor xi".ti OR "factor 11a".ti OR "factor twelve".ti OR "hageman factor".ti OR "factor 12".ti OR "coagulation factor xii".ti OR "activated factor xii".ti OR "coagulation factor xiia".ti OR "factor xiia".ti OR "blood coagulation factor xii".ti OR "activated prekallikrein".ti OR "prekallikrein".ti OR "factor xii".ti OR "activated hageman factor".ti OR "hageman factor fragment".ti OR "factor thirteen".ti OR "coagulation factor xiii".ti OR "factor xiii".ti OR "factor xiii".ti OR "transamidase".ti OR "fibrinase".ti OR "laki lorand factor".ti OR "blood coagulation factor xiii".ti OR "factor 13".ti OR "fibrin stabilizing factor".ti OR "stabilizing factor".ti OR "activated factor xiii".ti OR "factor 13a".ti OR "plasma transglutaminase".ti OR "transglutaminase".ti OR "beta thromboglobulin".ti OR "beta 2 thromboglobulin".ti OR "thromboglobulin".ti OR "fibrinopeptides a".ti,ab OR "fibrinopeptides b".ti OR "kalliginogenase".ti OR "kallikrein".ti OR "kallikrein kininogenase".ti OR "callicrein".ti OR "kinin forming enzyme".ti OR "kinin forming".ti OR "kallikrein light chain".ti OR "kallikrein padutin".ti OR "kallikrein a".ti OR "beta kallikrein".ti OR "kallikrein b".ti OR "alpha kallikrein".ti OR "plasma kallikrein".ti OR "kininogenin".ti OR "kallikrein i".ti OR "plasma prokallikrein".ti OR "prokallikrein".ti OR "prokinins".ti OR "cystatins".ti OR "t kininogen".ti OR "low molecular weight kininogens".ti OR "lmwk".ti OR "high molecular weight kininogens".ti OR "hmwk".ti OR "fitzgerald factor".ti OR "plasminogen".ti OR "plasminogen activator".ti OR "plasminogen activator inhibitors".ti OR "endothelial plasminogen activator".ti OR "endothelial plasminogen activator inhibitor".ti OR "pai 1".ti OR "serpin e1".ti OR "serpine1".ti OR "serpine1 protein".ti OR "type 1 plasminogen activator inhibitor".ti OR "serpin b2".ti OR "type 2 plasminogen activator inhibitor".ti OR "pai 2".ti OR "pai 3".ti OR "plasminogen activator inhibitor".ti OR "activated protein c inhibitor".ti OR "plasminogen activator inhibitor 3".ti OR "phosphorylcholine".ti OR "acetyl glyceryl phosphorylcholine".ti OR "aggregating factor".ti OR "platelet aggregation enhancing factor".ti OR "agepc".ti OR "thrombocyte aggregating activity".ti OR "alkyl 2 acetyl sn glycerophosphocholine".ti OR "1 alkyl 2 acetyl sn glyceryl 3 phosphorylcholine".ti OR "1 alkyl 2 acetylglycerophosphocholine".ti OR "paf acether".ti OR "platelet activating substance".ti OR "platelet activating substances".ti OR "pf 3".ti OR "platelet factor 3".ti OR "chemokine cxcl4".ti OR "cxcl4".ti OR "chemokine pf4".ti OR "platelet factor 4".ti OR "heparin neutralizing protein".ti OR "antiheparin factor".ti OR "thrombase".ti OR "thrombostat".ti OR "alpha thrombin".ti OR "thrombin jmi".ti OR "jmi".ti OR "thrombin".ti OR "beta thrombin".ti OR "gamma thrombin".ti OR "plasminogen activator".ti OR "tissue activator d 44".ti OR "tisokinase".ti OR "tissue type plasminogen activator".ti OR "ttpa".ti OR "t plasminogen activator".ti OR "tissue type activator".ti OR "rt pa".ti OR "factor viiir ag".ti OR "factor viiir rco".ti OR "ristocetin willebrand factor".ti OR "von willebrand protein".ti OR "von willebrand factor".ti OR "factor viii related antigen".ti OR "ristocetin cofactor".ti OR "plasma factor viii complex".ti OR "vitamin k dependent protein s".ti OR "cofactor protein s".ti OR "protein s".ti OR "protein c".ti OR "antiphospholipid".ti,ab OR "glutamate".ti,ab OR "beta 2 glycoprotein".ti,ab OR "anti beta 2 glycoprotein".ti,ab OR "adp".ti OR "adenosine diphosphate".ti OR "adenosine pyrophosphate".ti OR "adenosine 5' pyrophosphate".ti OR "serotonin".ti OR "5 hydroxytryptamine".ti OR "hippophaine".ti OR "3 2 aminoethyl 2 methyl 1 h indol 5 ol".ti OR "enteramine".ti OR "5 ht".ti OR "hydroxytryptamine".ti OR "thromboxane a2".ti OR "txa2".ti OR "arachidonic acid".ti OR "aa".ti OR "acetylsalicylic acid".ti OR "aspirin".ti OR "platelet derived growth factor".ti OR "pdgf receptor".ti OR "pdgf".ti OR "receptor tgf beta".ti OR "tgfbeta".ti OR "tgf beta".ti OR "platelet transforming growth factor".ti OR "gmp 140".ti OR "p selectin".ti OR "padgem".ti OR "cd62p antigen".ti OR "cd62p antigens".ti OR "cd62p".ti OR "alpha granule membrane protein".ti OR "lecam3".ti OR "gpvi".ti OR "platelet membrane glycoprotein p62".ti OR "glycoprotein gp vi".ti OR "platelet glycoprotein vi".ti OR "human glycoprotein vi".ti OR "human alpha2beta1".ti OR "integrin vla 2".ti OR "vla 2".ti OR "glycoprotein ia iia".ti OR "alpha2beta1integrin".ti OR "very late antigen 2".ti OR "late antigen 2".ti OR "cd49b cd29".ti OR "platelet membrane glycoprotein ia/iia".ti OR "platelet membrane glycoprotein ia iia".ti OR "platelet membrane glycoprotein ia/iia complex".ti OR "platelet membrane glycoprotein ia iia complex".ti OR "platelet glycoprotein gpiib iiia complex".ti OR "glycoproteins iib iiia".ti OR "glycoproteins iib iiia complex".ti OR "integrin alpha iib".ti OR "integrin alpha iib beta 3".ti OR "platelet glycoprotein gpib ix complex".ti OR "platelet glycoprotein gpib ix complex".ti OR "glycoprotein ib ix complex".ti OR "platelet membrane glycoprotein ib".ti OR "glycoprotein ib beta".ti OR "cd42c".ti OR "calcitonin gene related peptide".ti OR "calcitonin gene related peptide ii".ti OR "beta cgrp".ti OR "beta calcitonin gene related peptide".ti OR "alpha cgrp".ti OR "alpha calcitonin gene related peptide".ti OR "calcitonin gene related peptide i".ti OR "cgrp".ti OR "cyclo oxygenase i".ti OR "cox1".ti OR "prostaglandin h synthase 1".ti OR "prostaglandin synthase cyclooxygenase".ti OR "prostaglandin synthase".ti OR "cox 1 synthesis".ti OR "cox 1 prostaglandin".ti OR "cyclooxygenase 1".ti OR "endothelium derived vasoconstrictor factors".ti OR "vasoconstrictor factors".ti OR "endothelium derived endothelin 1".ti OR "nitric nitrogen".ti OR "endothelium derived nitric oxide".ti OR "vasodilator nitrates".ti OR "vasodilator nitric oxide".ti OR "von willebrand factor cleaving protease".ti OR "adamts13".ti OR "vwf cleaving protease".ti OR "vwf cleaving protease adamts13".ti OR "p2y12 receptors".ti OR "p2y12 receptor".ti OR "p2y12".ti OR "purinergic p2y12 receptors".ti OR "purinergic p2y12 receptor".ti OR "purinergic receptor p2y".ti OR "p2y adp receptor".ti OR "p2y adp receptors".ti OR "p selectin glycoprotein ligand 1".ti OR "p selectin".ti OR CD162.ti OR "platelet p selectin".ti OR "psgl 1 protein".ti OR "selectin P ligand protein".ti OR "endocannabinoid".ti OR "endocannabinoids".ti OR "cb2 receptor".ti OR "cannabinoid receptor cb2".ti OR "cannabinoid receptor 2".ti OR "cannabinoid receptor".ti OR "cannabinoid cb1 receptor".ti OR "cannabinoid cb2 receptor".ti OR "cannabinoid receptor cb1".ti OR "cannabinoid receptor 1".ti OR "cb1 cannabinoid receptor".ti OR "cb2 cannabinoid receptor".ti OR "glyceryl 2 arachidonate".ti OR "2 ag".ti OR "aea".ti OR "2 arachidonoylglycerol".ti OR "anandamide".ti OR "n arachidonoylethanolamine".ti OR "metalloproteinase 9".ti OR "matrix metalloproteinase 9".ti OR "mmp9".ti OR "metalloproteinase".ti OR "metallopeptidases".ti OR "metalloproteinases".ti OR "metallopeptidase".ti OR "type iv collagenase".ti OR "type iv collagenase mmp 2".ti OR "type iv collagenase mmp 9".ti OR "metalloproteinase mmp 9".ti OR "metalloproteinase 1".ti OR "mmp1".ti OR "metalloproteinase mmp1".ti OR "mmp 1".ti OR "mmp 9".ti OR "matrix metalloproteinase 1".ti OR "metalloproteinase 3".ti OR "mmp3".ti OR "matrix metalloproteinase 3".ti OR "metalloproteinase 2".ti OR "mmp2".ti OR "matrix metalloproteinase 2".ti OR "type iv collagenase mmp 2".ti OR "mmp 2 gelatinase a".ti) AND (*"Sex"/ OR *"Gender"/ OR *"gender and sex"/ OR "Gender".ti,ab OR "Sex".ti,ab OR exp *"Sexual Characteristics"/ OR "Sex Characteristic*".ti,ab OR "Sex Difference*".ti,ab OR "Gender Difference*".ti,ab OR "Sex Based".ti,ab OR "Sex Dimorphism*".ti,ab OR "Sexual Dimorphism*".ti,ab OR ((*"Female"/ OR *"Girl"/ OR "Women".ti,ab OR "Woman".ti,ab OR "Female".ti,ab) AND (*"Male"/ OR *"Boy"/ OR "Men".ti,ab OR "Man".ti,ab OR "Male".ti,ab))))) NOT ("Tissue Plasminogen Activator"/ OR "Aspirin") NOT (("Case Report"/ OR "case report".ti OR exp "Review"/ OR "review".ti OR "systematic review"/ OR "systematic review".ti OR exp "meta analysis"/ OR "meta-analysis".ti) NOT ("Clinical Study"/ OR exp "Clinical Trial"/ OR "trial".ti OR "RCT".ti)) NOT (therap*.ti OR treat*.ti) AND (english.la OR dutch.la) NOT ((exp "Child"/ OR exp "Infant"/) NOT (exp "Adult"/))

1. **Hemostatic related factors with ischemic stroke and migraine in women**

**PubMed**

((("stroke"[mesh] OR "stroke"[tw] OR "cva"[tw] OR "cerebrovascular accident"[tw] OR "cerebrovascular accidents"[tw] OR "cerebrovascular apoplexy"[tw] OR "apoplexy"[tw] OR "brain vascular accidents"[tw] OR "vascular accidents"[tw] OR "cerebrovascular stroke"[tw] OR "cerebrovascular strokes"[tw] OR "cerebrovascular apoplexy"[tw] OR "cerebral stroke"[tw] OR "cerebral strokes"[tw] OR "acute stroke"[tw] OR "acute strokes"[tw] OR "acute cerebrovascular accident"[tw] OR "acute cerebrovascular accidents"[tw] OR "cerebrovascular accidents"[tw] OR "brain infarction"[tw] OR "brain infarctions"[tw] OR "brain infarct"[tw] OR "brain infarcts"[tw] OR "anterior circulation infarction"[tw] OR "venous infarction"[tw] OR "venous infarctions"[tw] OR "posterior circulation infarction"[tw] OR "posterior circulation infarcts"[tw] OR "brain ischemia"[tw] OR "ischemic encephalopathy"[tw] OR "ischemic encephalopathies"[tw] OR "cerebral ischemia"[tw] OR "cerebral ischemias"[tw]) AND ("Migraine Disorders"[Mesh] OR "migraine"[tw] OR "migraine disorder"[tw] OR "migraine disorders"[tw] OR "migraines"[tw] OR "migraine headache"[tw] OR "migraine headaches"[tw] OR "headache"[tw] OR "headaches"[tw] OR "acute confusional migraine"[tw] OR "migraine hemicrania"[tw] OR "migraine variants"[tw] OR "migraine variant"[tw] OR "common migraine"[tw] OR "common migraines"[tw] OR "familial hemiplegic migraine"[tw] OR "familial hemiplegic migraines"[tw] OR "hemiplegic migraine"[tw] OR "classical migraine"[tw] OR "classical migraine attacks"[tw] OR "migraine with aura"[tw] OR "migraine with auras"[tw] OR "prolonged aura migraine"[tw] OR "migraineur"[tw] OR "migraineurs"[tw] OR "migraineurs without aura"[tw] OR "migrainous attack"[tw] OR "migrainous attacks"[tw] OR "migrainous aura"[tw] OR "migrainous auras"[tw]) AND ("blood coagulation"[tw] OR "blood coagulation factor"[tw] OR "blood coagulation factors"[tw] OR "coagulation factor"[tw] OR "coagulation factors"[tw] OR "clotting factor"[tw] OR "clotting factors"[tw] OR "blood clotting"[tw] OR "blood clotting factor"[tw] OR "blood clotting factors"[tw] OR "thromboses"[tw] OR "thrombus"[tw] OR "blood clot"[tw] OR "blood clots"[tw] OR "thrombus formation"[tw] OR "hemostases"[tw] OR "platelet activation"[tw] OR "platelet activations"[tw] OR "platelet aggregation"[tw] OR "platelet clotting"[tw] OR "platelet adhesion"[tw] OR "Blood Coagulation Factors"[majr] OR "factor i"[tw] OR "blood coagulation factor i"[tw] OR "coagulation factor i"[tw] OR "fibrinogen"[tw] OR "gamma fibrinogen"[tw] OR "gamma fibrinogen"[tw] OR "fi"[tw] OR "factor ii"[tw] OR "blood coagulation factor ii"[tw] OR "differentiation reversal factor"[tw] OR "coagulation factor ii"[tw] OR "prothrombin"[tw] OR "fii"[tw] OR "thromboplastin"[tw] OR "coagulin"[tw] OR "factor iii"[tw] OR "fiii"[tw] OR "coagulation factor iii"[tw] OR "coagulation tissue factor"[tw] OR "tissue thromboplastin"[tw] OR "thromboplastin"[tw] OR "procoagulant"[tw] OR "tissue factor procoagulant"[tw] OR "coagulation factor iv"[tw] OR "factor iv"[tw] OR "calcium 40"[tw] OR "calcium"[tw] OR "fv"[tw] OR "factor five"[tw] OR "blood coagulation factor v"[tw] OR "coagulation factor v"[tw] OR "coagulation factor v"[tw] OR "factor v"[tw] OR "activated factor v"[tw] OR "coagulation factor va"[tw] OR "factor va"[tw] OR "factor v leiden"[tw] OR "factor five leiden"[tw] OR "factor seven"[tw] OR "coagulation factor vii"[tw] OR "factor vii"[tw] OR "blood coagulation factor vii"[tw] OR "proconvertin"[tw] OR "coagulation factor 7"[tw] OR "factor 7"[tw] OR "coagulation factor viia"[tw] OR "factor viia"[tw] OR "activated factor vii"[tw] OR "activated factor vii"[tw] OR "factor 7a"[tw] OR "factor 8"[tw] OR "coagulation factor viii"[tw] OR "thromboplastinogen"[tw] OR "blood coagulation factor viii"[tw] OR "factor eight"[tw] OR "hyatt c"[tw] OR "factor viiic"[tw] OR "f viii c"[tw] OR "factor viii"[tw] OR "blood coagulation factor viii"[tw] OR "coagulation factor viiia"[tw] OR "factor viiia"[tw] OR "coagulation factor viii"[tw] OR "thrombin activated factor viii"[tw] OR "factor 8a"[tw] OR "activated factor viii"[tw] OR "factor nine"[tw] OR "autoprothrombin ii"[tw] OR "christmas factor"[tw] OR "factor ix"[tw] OR "blood coagulation factor ix"[tw] OR "factor ix complex"[tw] OR "factor 9"[tw] OR "coagulation factor ix"[tw] OR "coagulation factor ixa"[tw] OR "factor ixa"[tw] OR "activated factor ix"[tw] OR "factor 9a"[tw] OR "plasma thromboplastin component"[tw] OR "factor ten"[tw] OR "autoprothrombin iii"[tw] OR "coagulation factor x"[tw] OR "factor x"[tw] OR "blood coagulation factor x"[tw] OR "stuart factor"[tw] OR "stuart prower factor"[tw] OR "factor 10"[tw] OR "activated factor x"[tw] OR "activated coagulation factor x"[tw] OR "factor xa"[tw] OR "blood coagulation factor x"[tw] OR "factor 10a"[tw] OR "autoprothrombin c"[tw] OR "factor eleven"[tw] OR "blood coagulation factor xi"[tw] OR "plasma thromboplastin"[tw] OR "plasma thromboplastin antecedent"[tw] OR "antecedent"[tw] OR "plasma thromboplastin"[tw] OR "thromboplastin antecedent"[tw] OR "coagulation factor xi"[tw] OR "factor xi"[tw] OR "coagulation factor 11"[tw] OR "factor 11"[tw] OR "activated factor xi"[tw] OR "coagulation factor xia"[tw] OR "factor xia"[tw] OR "blood coagulation factor xi"[tw] OR "factor 11a"[tw] OR "factor twelve"[tw] OR "hageman factor"[tw] OR "factor 12"[tw] OR "coagulation factor xii"[tw] OR "activated factor xii"[tw] OR "coagulation factor xiia"[tw] OR "factor xiia"[tw] OR "blood coagulation factor xii"[tw] OR "activated prekallikrein"[tw] OR "prekallikrein"[tw] OR "factor xii"[tw] OR "activated hageman factor"[tw] OR "hageman factor fragment"[tw] OR "factor thirteen"[tw] OR "coagulation factor xiii"[tw] OR "factor xiii"[tw] OR "factor xiii"[tw] OR "transamidase"[tw] OR "fibrinase"[tw] OR "laki lorand factor"[tw] OR "blood coagulation factor xiii"[tw] OR "factor 13"[tw] OR "fibrin stabilizing factor"[tw] OR "stabilizing factor"[tw] OR "activated factor xiii"[tw] OR "factor 13a"[tw] OR "plasma transglutaminase"[tw] OR "transglutaminase"[tw] OR "beta thromboglobulin"[tw] OR "beta 2 thromboglobulin"[tw] OR "thromboglobulin"[tw] OR "fibrinopeptides a"[tw] OR "fibrinopeptides b"[tw] OR "kalliginogenase"[tw] OR "kallikrein"[tw] OR "kallikrein kininogenase"[tw] OR "callicrein"[tw] OR "kinin forming enzyme"[tw] OR "kinin forming"[tw] OR "kallikrein light chain"[tw] OR "kallikrein padutin"[tw] OR "kallikrein a"[tw] OR "beta kallikrein"[tw] OR "kallikrein b"[tw] OR "alpha kallikrein"[tw] OR "plasma kallikrein"[tw] OR "kininogenin"[tw] OR "kallikrein i"[tw] OR "plasma prokallikrein"[tw] OR "prokallikrein"[tw] OR "prokinins"[tw] OR "cystatins"[tw] OR "t kininogen"[tw] OR "low molecular weight kininogens"[tw] OR "lmwk"[tw] OR "high molecular weight kininogens"[tw] OR "hmwk"[tw] OR "fitzgerald factor"[tw] OR "plasminogen"[tw] OR "plasminogen activator"[tw] OR "plasminogen activator inhibitors"[tw] OR "endothelial plasminogen activator"[tw] OR "endothelial plasminogen activator inhibitor"[tw] OR "pai 1"[tw] OR "serpin e1"[tw] OR "serpine1"[tw] OR "serpine1 protein"[tw] OR "type 1 plasminogen activator inhibitor"[tw] OR "serpin b2"[tw] OR "type 2 plasminogen activator inhibitor"[tw] OR "pai 2"[tw] OR "pai 3"[tw] OR "plasminogen activator inhibitor"[tw] OR "activated protein c inhibitor"[tw] OR "plasminogen activator inhibitor 3"[tw] OR "phosphorylcholine"[tw] OR "acetyl glyceryl phosphorylcholine"[tw] OR "aggregating factor"[tw] OR "platelet aggregation enhancing factor"[tw] OR "agepc"[tw] OR "thrombocyte aggregating activity"[tw] OR "alkyl 2 acetyl sn glycerophosphocholine"[tw] OR "1 alkyl 2 acetyl sn glyceryl 3 phosphorylcholine"[tw] OR "1 alkyl 2 acetylglycerophosphocholine"[tw] OR "paf acether"[tw] OR "platelet activating substance"[tw] OR "platelet activating substances"[tw] OR "pf 3"[tw] OR "platelet factor 3"[tw] OR "chemokine cxcl4"[tw] OR "cxcl4"[tw] OR "chemokine pf4"[tw] OR "platelet factor 4"[tw] OR "heparin neutralizing protein"[tw] OR "antiheparin factor"[tw] OR "thrombase"[tw] OR "thrombostat"[tw] OR "alpha thrombin"[tw] OR "thrombin jmi"[tw] OR "jmi"[tw] OR "thrombin"[tw] OR "beta thrombin"[tw] OR "gamma thrombin"[tw] OR "plasminogen activator"[tw] OR "tissue activator d 44"[tw] OR "tisokinase"[tw] OR "tissue type plasminogen activator"[tw] OR "ttpa"[tw] OR "t plasminogen activator"[tw] OR "tissue type activator"[tw] OR "rt pa"[tw] OR "factor viiir ag"[tw] OR "factor viiir rco"[tw] OR "ristocetin willebrand factor"[tw] OR "von willebrand protein"[tw] OR "von willebrand factor"[tw] OR "factor viii related antigen"[tw] OR "ristocetin cofactor"[tw] OR "plasma factor viii complex"[tw] OR "vitamin k dependent protein s"[tw] OR "cofactor protein s"[tw] OR "protein s"[tw] OR "protein c"[tw] OR "antiphospholipid"[tw] OR "glutamate"[tw] OR "beta 2 glycoprotein"[tw] OR "anti beta 2 glycoprotein"[tw] OR "adp"[tw] OR "adenosine diphosphate"[tw] OR "adenosine pyrophosphate"[tw] OR "adenosine 5' pyrophosphate"[tw] OR "serotonin"[tw] OR "5 hydroxytryptamine"[tw] OR "hippophaine"[tw] OR "3 2 aminoethyl 2 methyl 1 h indol 5 ol"[tw] OR "enteramine"[tw] OR "5 ht"[tw] OR "hydroxytryptamine"[tw] OR "thromboxane a2"[tw] OR "txa2"[tw] OR "arachidonic acid"[tw] OR "aa"[tw] OR "acetylsalicylic acid"[tw] OR "aspirin"[tw] OR "platelet derived growth factor"[tw] OR "pdgf receptor"[tw] OR "pdgf"[tw] OR "receptor tgf beta"[tw] OR "tgfbeta"[tw] OR "tgf beta"[tw] OR "platelet transforming growth factor"[tw] OR "gmp 140"[tw] OR "p selectin"[tw] OR "padgem"[tw] OR "cd62p antigen"[tw] OR "cd62p antigens"[tw] OR "cd62p"[tw] OR "alpha granule membrane protein"[tw] OR "lecam3"[tw] OR "gpvi"[tw] OR "platelet membrane glycoprotein p62"[tw] OR "glycoprotein gp vi"[tw] OR "platelet glycoprotein vi"[tw] OR "human glycoprotein vi"[tw] OR "human alpha2beta1"[tw] OR "integrin vla 2"[tw] OR "vla 2"[tw] OR "glycoprotein ia iia"[tw] OR "alpha2beta1integrin"[tw] OR "very late antigen 2"[tw] OR "late antigen 2"[tw] OR "cd49b cd29"[tw] OR "platelet membrane glycoprotein ia/iia"[tw] OR "platelet membrane glycoprotein ia iia"[tw] OR "platelet membrane glycoprotein ia/iia complex"[tw] OR "platelet membrane glycoprotein ia iia complex"[tw] OR "platelet glycoprotein gpiib iiia complex"[tw] OR "glycoproteins iib iiia"[tw] OR "glycoproteins iib iiia complex"[tw] OR "integrin alpha iib"[tw] OR "integrin alpha iib beta 3"[tw] OR "platelet glycoprotein gpib ix complex"[tw] OR "platelet glycoprotein gpib ix complex"[tw] OR "glycoprotein ib ix complex"[tw] OR "platelet membrane glycoprotein ib"[tw] OR "glycoprotein ib beta"[tw] OR "cd42c"[tw] OR "calcitonin gene related peptide"[tw] OR "calcitonin gene related peptide ii"[tw] OR "beta cgrp"[tw] OR "beta calcitonin gene related peptide"[tw] OR "alpha cgrp"[tw] OR "alpha calcitonin gene related peptide"[tw] OR "calcitonin gene related peptide i"[tw] OR "cgrp"[tw] OR "cyclo oxygenase i"[tw] OR "cox1"[tw] OR "prostaglandin h synthase 1"[tw] OR "prostaglandin synthase cyclooxygenase"[tw] OR "prostaglandin synthase"[tw] OR "cox 1 synthesis"[tw] OR "cox 1 prostaglandin"[tw] OR "cyclooxygenase 1"[tw] OR "endothelium derived vasoconstrictor factors"[tw] OR "vasoconstrictor factors"[tw] OR "endothelium derived endothelin 1"[tw] OR "nitric nitrogen"[tw] OR "endothelium derived nitric oxide"[tw] OR "vasodilator nitrates"[tw] OR "vasodilator nitric oxide"[tw] OR "von willebrand factor cleaving protease"[tw] OR "adamts13"[tw] OR "vwf cleaving protease"[tw] OR "vwf cleaving protease adamts13"[tw] OR "p2y12 receptors"[tw] OR "p2y12 receptor"[tw] OR "p2y12"[tw] OR "purinergic p2y12 receptors"[tw] OR "purinergic p2y12 receptor"[tw] OR "purinergic receptor p2y"[tw] OR "p2y adp receptor"[tw] OR "p2y adp receptors"[tw] OR "p selectin glycoprotein ligand 1"[tw] OR "p selectin"[tw] OR CD162[tw] OR "platelet p selectin"[tw] OR "psgl 1 protein"[tw] OR "selectin P ligand protein"[tw] OR "endocannabinoid"[tw] OR "endocannabinoids"[tw] OR "cb2 receptor"[tw] OR "cannabinoid receptor cb2"[tw] OR "cannabinoid receptor 2"[tw] OR "cannabinoid receptor"[tw] OR "cannabinoid cb1 receptor"[tw] OR "cannabinoid cb2 receptor"[tw] OR "cannabinoid receptor cb1"[tw] OR "cannabinoid receptor 1"[tw] OR "cb1 cannabinoid receptor"[tw] OR "cb2 cannabinoid receptor"[tw] OR "glyceryl 2 arachidonate"[tw] OR "2 ag"[tw] OR "aea"[tw] OR "2 arachidonoylglycerol"[tw] OR "anandamide"[tw] OR "n arachidonoylethanolamine"[tw] OR "metalloproteinase 9"[tw] OR "matrix metalloproteinase 9"[tw] OR "mmp9"[tw] OR "metalloproteinase"[tw] OR "metallopeptidases"[tw] OR "metalloproteinases"[tw] OR "metallopeptidase"[tw] OR "type iv collagenase"[tw] OR "type iv collagenase mmp 2"[tw] OR "type iv collagenase mmp 9"[tw] OR "metalloproteinase mmp 9"[tw] OR "metalloproteinase 1"[tw] OR "mmp1"[tw] OR "metalloproteinase mmp1"[tw] OR "mmp 1"[tw] OR "mmp 9"[tw] OR "matrix metalloproteinase 1"[tw] OR "metalloproteinase 3"[tw] OR "mmp3"[tw] OR "matrix metalloproteinase 3"[tw] OR "metalloproteinase 2"[tw] OR "mmp2"[tw] OR "matrix metalloproteinase 2"[tw] OR "type iv collagenase mmp 2"[tw] OR "mmp 2 gelatinase a"[tw]) AND ("female"[tiab] OR "females"[tiab] OR "women"[tiab] OR "woman"[tiab] OR "girl"[tiab] OR "girls"[tiab])) OR (("stroke"[mesh] OR "stroke"[tw] OR "cva"[tw] OR "cerebrovascular accident"[tw] OR "cerebrovascular accidents"[tw] OR "cerebrovascular apoplexy"[tw] OR "apoplexy"[tw] OR "brain vascular accidents"[tw] OR "vascular accidents"[tw] OR "cerebrovascular stroke"[tw] OR "cerebrovascular strokes"[tw] OR "cerebrovascular apoplexy"[tw] OR "cerebral stroke"[tw] OR "cerebral strokes"[tw] OR "acute stroke"[tw] OR "acute strokes"[tw] OR "acute cerebrovascular accident"[tw] OR "acute cerebrovascular accidents"[tw] OR "cerebrovascular accidents"[tw] OR "brain infarction"[tw] OR "brain infarctions"[tw] OR "brain infarct"[tw] OR "brain infarcts"[tw] OR "anterior circulation infarction"[tw] OR "venous infarction"[tw] OR "venous infarctions"[tw] OR "posterior circulation infarction"[tw] OR "posterior circulation infarcts"[tw] OR "brain ischemia"[tw] OR "ischemic encephalopathy"[tw] OR "ischemic encephalopathies"[tw] OR "cerebral ischemia"[tw] OR "cerebral ischemias"[tw]) AND ("blood coagulation"[tw] OR "blood coagulation factor"[tw] OR "blood coagulation factors"[tw] OR "coagulation factor"[tw] OR "coagulation factors"[tw] OR "clotting factor"[tw] OR "clotting factors"[tw] OR "blood clotting"[tw] OR "blood clotting factor"[tw] OR "blood clotting factors"[tw] OR "thromboses"[tw] OR "thrombus"[tw] OR "blood clot"[tw] OR "blood clots"[tw] OR "thrombus formation"[tw] OR "hemostases"[tw] OR "platelet activation"[tw] OR "platelet activations"[tw] OR "platelet aggregation"[tw] OR "platelet clotting"[tw] OR "platelet adhesion"[tw] OR "Blood Coagulation Factors"[majr] OR "factor i"[tw] OR "blood coagulation factor i"[tw] OR "coagulation factor i"[tw] OR "fibrinogen"[tw] OR "gamma fibrinogen"[tw] OR "gamma fibrinogen"[tw] OR "fi"[tw] OR "factor ii"[tw] OR "blood coagulation factor ii"[tw] OR "differentiation reversal factor"[tw] OR "coagulation factor ii"[tw] OR "prothrombin"[tw] OR "fii"[tw] OR "thromboplastin"[tw] OR "coagulin"[tw] OR "factor iii"[tw] OR "fiii"[tw] OR "coagulation factor iii"[tw] OR "coagulation tissue factor"[tw] OR "tissue thromboplastin"[tw] OR "thromboplastin"[tw] OR "procoagulant"[tw] OR "tissue factor procoagulant"[tw] OR "coagulation factor iv"[tw] OR "factor iv"[tw] OR "calcium 40"[tw] OR "calcium"[tw] OR "fv"[tw] OR "factor five"[tw] OR "blood coagulation factor v"[tw] OR "coagulation factor v"[tw] OR "coagulation factor v"[tw] OR "factor v"[tw] OR "activated factor v"[tw] OR "coagulation factor va"[tw] OR "factor va"[tw] OR "factor v leiden"[tw] OR "factor five leiden"[tw] OR "factor seven"[tw] OR "coagulation factor vii"[tw] OR "factor vii"[tw] OR "blood coagulation factor vii"[tw] OR "proconvertin"[tw] OR "coagulation factor 7"[tw] OR "factor 7"[tw] OR "coagulation factor viia"[tw] OR "factor viia"[tw] OR "activated factor vii"[tw] OR "activated factor vii"[tw] OR "factor 7a"[tw] OR "factor 8"[tw] OR "coagulation factor viii"[tw] OR "thromboplastinogen"[tw] OR "blood coagulation factor viii"[tw] OR "factor eight"[tw] OR "hyatt c"[tw] OR "factor viiic"[tw] OR "f viii c"[tw] OR "factor viii"[tw] OR "blood coagulation factor viii"[tw] OR "coagulation factor viiia"[tw] OR "factor viiia"[tw] OR "coagulation factor viii"[tw] OR "thrombin activated factor viii"[tw] OR "factor 8a"[tw] OR "activated factor viii"[tw] OR "factor nine"[tw] OR "autoprothrombin ii"[tw] OR "christmas factor"[tw] OR "factor ix"[tw] OR "blood coagulation factor ix"[tw] OR "factor ix complex"[tw] OR "factor 9"[tw] OR "coagulation factor ix"[tw] OR "coagulation factor ixa"[tw] OR "factor ixa"[tw] OR "activated factor ix"[tw] OR "factor 9a"[tw] OR "plasma thromboplastin component"[tw] OR "factor ten"[tw] OR "autoprothrombin iii"[tw] OR "coagulation factor x"[tw] OR "factor x"[tw] OR "blood coagulation factor x"[tw] OR "stuart factor"[tw] OR "stuart prower factor"[tw] OR "factor 10"[tw] OR "activated factor x"[tw] OR "activated coagulation factor x"[tw] OR "factor xa"[tw] OR "blood coagulation factor x"[tw] OR "factor 10a"[tw] OR "autoprothrombin c"[tw] OR "factor eleven"[tw] OR "blood coagulation factor xi"[tw] OR "plasma thromboplastin"[tw] OR "plasma thromboplastin antecedent"[tw] OR "antecedent"[tw] OR "plasma thromboplastin"[tw] OR "thromboplastin antecedent"[tw] OR "coagulation factor xi"[tw] OR "factor xi"[tw] OR "coagulation factor 11"[tw] OR "factor 11"[tw] OR "activated factor xi"[tw] OR "coagulation factor xia"[tw] OR "factor xia"[tw] OR "blood coagulation factor xi"[tw] OR "factor 11a"[tw] OR "factor twelve"[tw] OR "hageman factor"[tw] OR "factor 12"[tw] OR "coagulation factor xii"[tw] OR "activated factor xii"[tw] OR "coagulation factor xiia"[tw] OR "factor xiia"[tw] OR "blood coagulation factor xii"[tw] OR "activated prekallikrein"[tw] OR "prekallikrein"[tw] OR "factor xii"[tw] OR "activated hageman factor"[tw] OR "hageman factor fragment"[tw] OR "factor thirteen"[tw] OR "coagulation factor xiii"[tw] OR "factor xiii"[tw] OR "factor xiii"[tw] OR "transamidase"[tw] OR "fibrinase"[tw] OR "laki lorand factor"[tw] OR "blood coagulation factor xiii"[tw] OR "factor 13"[tw] OR "fibrin stabilizing factor"[tw] OR "stabilizing factor"[tw] OR "activated factor xiii"[tw] OR "factor 13a"[tw] OR "plasma transglutaminase"[tw] OR "transglutaminase"[tw] OR "beta thromboglobulin"[tw] OR "beta 2 thromboglobulin"[tw] OR "thromboglobulin"[tw] OR "fibrinopeptides a"[tw] OR "fibrinopeptides b"[tw] OR "kalliginogenase"[tw] OR "kallikrein"[tw] OR "kallikrein kininogenase"[tw] OR "callicrein"[tw] OR "kinin forming enzyme"[tw] OR "kinin forming"[tw] OR "kallikrein light chain"[tw] OR "kallikrein padutin"[tw] OR "kallikrein a"[tw] OR "beta kallikrein"[tw] OR "kallikrein b"[tw] OR "alpha kallikrein"[tw] OR "plasma kallikrein"[tw] OR "kininogenin"[tw] OR "kallikrein i"[tw] OR "plasma prokallikrein"[tw] OR "prokallikrein"[tw] OR "prokinins"[tw] OR "cystatins"[tw] OR "t kininogen"[tw] OR "low molecular weight kininogens"[tw] OR "lmwk"[tw] OR "high molecular weight kininogens"[tw] OR "hmwk"[tw] OR "fitzgerald factor"[tw] OR "plasminogen"[tw] OR "plasminogen activator"[tw] OR "plasminogen activator inhibitors"[tw] OR "endothelial plasminogen activator"[tw] OR "endothelial plasminogen activator inhibitor"[tw] OR "pai 1"[tw] OR "serpin e1"[tw] OR "serpine1"[tw] OR "serpine1 protein"[tw] OR "type 1 plasminogen activator inhibitor"[tw] OR "serpin b2"[tw] OR "type 2 plasminogen activator inhibitor"[tw] OR "pai 2"[tw] OR "pai 3"[tw] OR "plasminogen activator inhibitor"[tw] OR "activated protein c inhibitor"[tw] OR "plasminogen activator inhibitor 3"[tw] OR "phosphorylcholine"[tw] OR "acetyl glyceryl phosphorylcholine"[tw] OR "aggregating factor"[tw] OR "platelet aggregation enhancing factor"[tw] OR "agepc"[tw] OR "thrombocyte aggregating activity"[tw] OR "alkyl 2 acetyl sn glycerophosphocholine"[tw] OR "1 alkyl 2 acetyl sn glyceryl 3 phosphorylcholine"[tw] OR "1 alkyl 2 acetylglycerophosphocholine"[tw] OR "paf acether"[tw] OR "platelet activating substance"[tw] OR "platelet activating substances"[tw] OR "pf 3"[tw] OR "platelet factor 3"[tw] OR "chemokine cxcl4"[tw] OR "cxcl4"[tw] OR "chemokine pf4"[tw] OR "platelet factor 4"[tw] OR "heparin neutralizing protein"[tw] OR "antiheparin factor"[tw] OR "thrombase"[tw] OR "thrombostat"[tw] OR "alpha thrombin"[tw] OR "thrombin jmi"[tw] OR "jmi"[tw] OR "thrombin"[tw] OR "beta thrombin"[tw] OR "gamma thrombin"[tw] OR "plasminogen activator"[tw] OR "tissue activator d 44"[tw] OR "tisokinase"[tw] OR "tissue type plasminogen activator"[tw] OR "ttpa"[tw] OR "t plasminogen activator"[tw] OR "tissue type activator"[tw] OR "rt pa"[tw] OR "factor viiir ag"[tw] OR "factor viiir rco"[tw] OR "ristocetin willebrand factor"[tw] OR "von willebrand protein"[tw] OR "von willebrand factor"[tw] OR "factor viii related antigen"[tw] OR "ristocetin cofactor"[tw] OR "plasma factor viii complex"[tw] OR "vitamin k dependent protein s"[tw] OR "cofactor protein s"[tw] OR "protein s"[tw] OR "protein c"[tw] OR "antiphospholipid"[tw] OR "glutamate"[tw] OR "beta 2 glycoprotein"[tw] OR "anti beta 2 glycoprotein"[tw] OR "adp"[tw] OR "adenosine diphosphate"[tw] OR "adenosine pyrophosphate"[tw] OR "adenosine 5' pyrophosphate"[tw] OR "serotonin"[tw] OR "5 hydroxytryptamine"[tw] OR "hippophaine"[tw] OR "3 2 aminoethyl 2 methyl 1 h indol 5 ol"[tw] OR "enteramine"[tw] OR "5 ht"[tw] OR "hydroxytryptamine"[tw] OR "thromboxane a2"[tw] OR "txa2"[tw] OR "arachidonic acid"[tw] OR "aa"[tw] OR "acetylsalicylic acid"[tw] OR "aspirin"[tw] OR "platelet derived growth factor"[tw] OR "pdgf receptor"[tw] OR "pdgf"[tw] OR "receptor tgf beta"[tw] OR "tgfbeta"[tw] OR "tgf beta"[tw] OR "platelet transforming growth factor"[tw] OR "gmp 140"[tw] OR "p selectin"[tw] OR "padgem"[tw] OR "cd62p antigen"[tw] OR "cd62p antigens"[tw] OR "cd62p"[tw] OR "alpha granule membrane protein"[tw] OR "lecam3"[tw] OR "gpvi"[tw] OR "platelet membrane glycoprotein p62"[tw] OR "glycoprotein gp vi"[tw] OR "platelet glycoprotein vi"[tw] OR "human glycoprotein vi"[tw] OR "human alpha2beta1"[tw] OR "integrin vla 2"[tw] OR "vla 2"[tw] OR "glycoprotein ia iia"[tw] OR "alpha2beta1integrin"[tw] OR "very late antigen 2"[tw] OR "late antigen 2"[tw] OR "cd49b cd29"[tw] OR "platelet membrane glycoprotein ia/iia"[tw] OR "platelet membrane glycoprotein ia iia"[tw] OR "platelet membrane glycoprotein ia/iia complex"[tw] OR "platelet membrane glycoprotein ia iia complex"[tw] OR "platelet glycoprotein gpiib iiia complex"[tw] OR "glycoproteins iib iiia"[tw] OR "glycoproteins iib iiia complex"[tw] OR "integrin alpha iib"[tw] OR "integrin alpha iib beta 3"[tw] OR "platelet glycoprotein gpib ix complex"[tw] OR "platelet glycoprotein gpib ix complex"[tw] OR "glycoprotein ib ix complex"[tw] OR "platelet membrane glycoprotein ib"[tw] OR "glycoprotein ib beta"[tw] OR "cd42c"[tw] OR "calcitonin gene related peptide"[tw] OR "calcitonin gene related peptide ii"[tw] OR "beta cgrp"[tw] OR "beta calcitonin gene related peptide"[tw] OR "alpha cgrp"[tw] OR "alpha calcitonin gene related peptide"[tw] OR "calcitonin gene related peptide i"[tw] OR "cgrp"[tw] OR "cyclo oxygenase i"[tw] OR "cox1"[tw] OR "prostaglandin h synthase 1"[tw] OR "prostaglandin synthase cyclooxygenase"[tw] OR "prostaglandin synthase"[tw] OR "cox 1 synthesis"[tw] OR "cox 1 prostaglandin"[tw] OR "cyclooxygenase 1"[tw] OR "endothelium derived vasoconstrictor factors"[tw] OR "vasoconstrictor factors"[tw] OR "endothelium derived endothelin 1"[tw] OR "nitric nitrogen"[tw] OR "endothelium derived nitric oxide"[tw] OR "vasodilator nitrates"[tw] OR "vasodilator nitric oxide"[tw] OR "von willebrand factor cleaving protease"[tw] OR "adamts13"[tw] OR "vwf cleaving protease"[tw] OR "vwf cleaving protease adamts13"[tw] OR "p2y12 receptors"[tw] OR "p2y12 receptor"[tw] OR "p2y12"[tw] OR "purinergic p2y12 receptors"[tw] OR "purinergic p2y12 receptor"[tw] OR "purinergic receptor p2y"[tw] OR "p2y adp receptor"[tw] OR "p2y adp receptors"[tw] OR "p selectin glycoprotein ligand 1"[tw] OR "p selectin"[tw] OR CD162[tw] OR "platelet p selectin"[tw] OR "psgl 1 protein"[tw] OR "selectin P ligand protein"[tw] OR "endocannabinoid"[tw] OR "endocannabinoids"[tw] OR "cb2 receptor"[tw] OR "cannabinoid receptor cb2"[tw] OR "cannabinoid receptor 2"[tw] OR "cannabinoid receptor"[tw] OR "cannabinoid cb1 receptor"[tw] OR "cannabinoid cb2 receptor"[tw] OR "cannabinoid receptor cb1"[tw] OR "cannabinoid receptor 1"[tw] OR "cb1 cannabinoid receptor"[tw] OR "cb2 cannabinoid receptor"[tw] OR "glyceryl 2 arachidonate"[tw] OR "2 ag"[tw] OR "aea"[tw] OR "2 arachidonoylglycerol"[tw] OR "anandamide"[tw] OR "n arachidonoylethanolamine"[tw] OR "metalloproteinase 9"[tw] OR "matrix metalloproteinase 9"[tw] OR "mmp9"[tw] OR "metalloproteinase"[tw] OR "metallopeptidases"[tw] OR "metalloproteinases"[tw] OR "metallopeptidase"[tw] OR "type iv collagenase"[tw] OR "type iv collagenase mmp 2"[tw] OR "type iv collagenase mmp 9"[tw] OR "metalloproteinase mmp 9"[tw] OR "metalloproteinase 1"[tw] OR "mmp1"[tw] OR "metalloproteinase mmp1"[tw] OR "mmp 1"[tw] OR "mmp 9"[tw] OR "matrix metalloproteinase 1"[tw] OR "metalloproteinase 3"[tw] OR "mmp3"[tw] OR "matrix metalloproteinase 3"[tw] OR "metalloproteinase 2"[tw] OR "mmp2"[tw] OR "matrix metalloproteinase 2"[tw] OR "type iv collagenase mmp 2"[tw] OR "mmp 2 gelatinase a"[tw]) AND ("female"[tiab] OR "females"[tiab] OR "women"[tiab] OR "woman"[tiab] OR "girl"[tiab] OR "girls"[tiab]))) NOT (("Case Reports"[ptyp] OR "case report"[ti] OR "Review"[ptyp] OR "review"[ti] OR "systematic"[sb] OR "systematic review"[ti] OR "meta-analysis"[ptyp] OR "meta-analysis"[ti]) NOT ("Clinical Study"[ptyp] OR "trial"[ti] OR "RCT"[ti])) NOT ("Stroke/therapy"[majr] OR therap*[ti] OR treat*[ti]) AND (english[la] OR dutch[la]) NOT (("Child"[mesh] OR "Infant"[mesh]) NOT ("Adult"[mesh])) NOT ("Tissue Plasminogen Activator/pharmacology"[Mesh] OR "Aspirin/pharmacology"[Mesh] OR "Stroke/therapy"[Mesh])

**Embase**

(((exp "cerebrovascular accident"/ OR "stroke".ti,ab OR "cva".ti,ab OR "cerebrovascular accident".ti,ab OR "cerebrovascular accidents".ti,ab OR "cerebrovascular apoplexy".ti,ab OR "apoplexy".ti,ab OR "brain vascular accidents".ti,ab OR "vascular accidents".ti,ab OR "cerebrovascular stroke".ti,ab OR "cerebrovascular strokes".ti,ab OR "cerebrovascular apoplexy".ti,ab OR "cerebral stroke".ti,ab OR "cerebral strokes".ti,ab OR "acute stroke".ti,ab OR "acute strokes".ti,ab OR "acute cerebrovascular accident".ti,ab OR "acute cerebrovascular accidents".ti,ab OR "cerebrovascular accidents".ti,ab OR "brain infarction".ti,ab OR "brain infarctions".ti,ab OR "brain infarct".ti,ab OR "brain infarcts".ti,ab OR "anterior circulation infarction".ti,ab OR "venous infarction".ti,ab OR "venous infarctions".ti,ab OR "posterior circulation infarction".ti,ab OR "posterior circulation infarcts".ti,ab OR "brain ischemia".ti,ab OR "ischemic encephalopathy".ti,ab OR "ischemic encephalopathies".ti,ab OR "cerebral ischemia".ti,ab OR "cerebral ischemias".ti,ab) AND (exp "Migraine"/ OR "migraine".ti,ab OR "migraine disorder".ti,ab OR "migraine disorders".ti,ab OR "migraines".ti,ab OR "migraine headache".ti,ab OR "migraine headaches".ti,ab OR "headache".ti,ab OR "headaches".ti,ab OR "acute confusional migraine".ti,ab OR "migraine hemicrania".ti,ab OR "migraine variants".ti,ab OR "migraine variant".ti,ab OR "common migraine".ti,ab OR "common migraines".ti,ab OR "familial hemiplegic migraine".ti,ab OR "familial hemiplegic migraines".ti,ab OR "hemiplegic migraine".ti,ab OR "classical migraine".ti,ab OR "classical migraine attacks".ti,ab OR "migraine with aura".ti,ab OR "migraine with auras".ti,ab OR "prolonged aura migraine".ti,ab OR "migraineur".ti,ab OR "migraineurs".ti,ab OR "migraineurs without aura".ti,ab OR "migrainous attack".ti,ab OR "migrainous attacks".ti,ab OR "migrainous aura".ti,ab OR "migrainous auras".ti,ab) AND ("blood coagulation".ti,ab OR "blood coagulation factor".ti,ab OR "blood coagulation factors".ti,ab OR "coagulation factor".ti,ab OR "coagulation factors".ti,ab OR "clotting factor".ti,ab OR "clotting factors".ti,ab OR "blood clotting".ti,ab OR "blood clotting factor".ti,ab OR "blood clotting factors".ti,ab OR "thromboses".ti,ab OR "thrombus".ti,ab OR "blood clot".ti,ab OR "blood clots".ti,ab OR "thrombus formation".ti,ab OR "hemostases".ti,ab OR "platelet activation".ti,ab OR "platelet activations".ti,ab OR "platelet aggregation".ti,ab OR "platelet clotting".ti,ab OR "platelet adhesion".ti,ab OR exp *"Blood Clotting Factor"/ OR "factor i".ti,ab OR "blood coagulation factor i".ti,ab OR "coagulation factor i".ti,ab OR "fibrinogen".ti,ab OR "gamma fibrinogen".ti,ab OR "gamma fibrinogen".ti,ab OR "fi".ti,ab OR "factor ii".ti,ab OR "blood coagulation factor ii".ti,ab OR "differentiation reversal factor".ti,ab OR "coagulation factor ii".ti,ab OR "prothrombin".ti,ab OR "fii".ti,ab OR "thromboplastin".ti,ab OR "coagulin".ti,ab OR "factor iii".ti,ab OR "fiii".ti,ab OR "coagulation factor iii".ti,ab OR "coagulation tissue factor".ti,ab OR "tissue thromboplastin".ti,ab OR "thromboplastin".ti,ab OR "procoagulant".ti,ab OR "tissue factor procoagulant".ti,ab OR "coagulation factor iv".ti,ab OR "factor iv".ti,ab OR "calcium 40".ti,ab OR "calcium".ti,ab OR "fv".ti,ab OR "factor five".ti,ab OR "blood coagulation factor v".ti,ab OR "coagulation factor v".ti,ab OR "coagulation factor v".ti,ab OR "factor v".ti,ab OR "activated factor v".ti,ab OR "coagulation factor va".ti,ab OR "factor va".ti,ab OR "factor v leiden".ti,ab OR "factor five leiden".ti,ab OR "factor seven".ti,ab OR "coagulation factor vii".ti,ab OR "factor vii".ti,ab OR "blood coagulation factor vii".ti,ab OR "proconvertin".ti,ab OR "coagulation factor 7".ti,ab OR "factor 7".ti,ab OR "coagulation factor viia".ti,ab OR "factor viia".ti,ab OR "activated factor vii".ti,ab OR "activated factor vii".ti,ab OR "factor 7a".ti,ab OR "factor 8".ti,ab OR "coagulation factor viii".ti,ab OR "thromboplastinogen".ti,ab OR "blood coagulation factor viii".ti,ab OR "factor eight".ti,ab OR "hyatt c".ti,ab OR "factor viiic".ti,ab OR "f viii c".ti,ab OR "factor viii".ti,ab OR "blood coagulation factor viii".ti,ab OR "coagulation factor viiia".ti,ab OR "factor viiia".ti,ab OR "coagulation factor viii".ti,ab OR "thrombin activated factor viii".ti,ab OR "factor 8a".ti,ab OR "activated factor viii".ti,ab OR "factor nine".ti,ab OR "autoprothrombin ii".ti,ab OR "christmas factor".ti,ab OR "factor ix".ti,ab OR "blood coagulation factor ix".ti,ab OR "factor ix complex".ti,ab OR "factor 9".ti,ab OR "coagulation factor ix".ti,ab OR "coagulation factor ixa".ti,ab OR "factor ixa".ti,ab OR "activated factor ix".ti,ab OR "factor 9a".ti,ab OR "plasma thromboplastin component".ti,ab OR "factor ten".ti,ab OR "autoprothrombin iii".ti,ab OR "coagulation factor x".ti,ab OR "factor x".ti,ab OR "blood coagulation factor x".ti,ab OR "stuart factor".ti,ab OR "stuart prower factor".ti,ab OR "factor 10".ti,ab OR "activated factor x".ti,ab OR "activated coagulation factor x".ti,ab OR "factor xa".ti,ab OR "blood coagulation factor x".ti,ab OR "factor 10a".ti,ab OR "autoprothrombin c".ti,ab OR "factor eleven".ti,ab OR "blood coagulation factor xi".ti,ab OR "plasma thromboplastin".ti,ab OR "plasma thromboplastin antecedent".ti,ab OR "antecedent".ti,ab OR "plasma thromboplastin".ti,ab OR "thromboplastin antecedent".ti,ab OR "coagulation factor xi".ti,ab OR "factor xi".ti,ab OR "coagulation factor 11".ti,ab OR "factor 11".ti,ab OR "activated factor xi".ti,ab OR "coagulation factor xia".ti,ab OR "factor xia".ti,ab OR "blood coagulation factor xi".ti,ab OR "factor 11a".ti,ab OR "factor twelve".ti,ab OR "hageman factor".ti,ab OR "factor 12".ti,ab OR "coagulation factor xii".ti,ab OR "activated factor xii".ti,ab OR "coagulation factor xiia".ti,ab OR "factor xiia".ti,ab OR "blood coagulation factor xii".ti,ab OR "activated prekallikrein".ti,ab OR "prekallikrein".ti,ab OR "factor xii".ti,ab OR "activated hageman factor".ti,ab OR "hageman factor fragment".ti,ab OR "factor thirteen".ti,ab OR "coagulation factor xiii".ti,ab OR "factor xiii".ti,ab OR "factor xiii".ti,ab OR "transamidase".ti,ab OR "fibrinase".ti,ab OR "laki lorand factor".ti,ab OR "blood coagulation factor xiii".ti,ab OR "factor 13".ti,ab OR "fibrin stabilizing factor".ti,ab OR "stabilizing factor".ti,ab OR "activated factor xiii".ti,ab OR "factor 13a".ti,ab OR "plasma transglutaminase".ti,ab OR "transglutaminase".ti,ab OR "beta thromboglobulin".ti,ab OR "beta 2 thromboglobulin".ti,ab OR "thromboglobulin".ti,ab OR "fibrinopeptides a".ti,ab OR "fibrinopeptides b".ti,ab OR "kalliginogenase".ti,ab OR "kallikrein".ti,ab OR "kallikrein kininogenase".ti,ab OR "callicrein".ti,ab OR "kinin forming enzyme".ti,ab OR "kinin forming".ti,ab OR "kallikrein light chain".ti,ab OR "kallikrein padutin".ti,ab OR "kallikrein a".ti,ab OR "beta kallikrein".ti,ab OR "kallikrein b".ti,ab OR "alpha kallikrein".ti,ab OR "plasma kallikrein".ti,ab OR "kininogenin".ti,ab OR "kallikrein i".ti,ab OR "plasma prokallikrein".ti,ab OR "prokallikrein".ti,ab OR "prokinins".ti,ab OR "cystatins".ti,ab OR "t kininogen".ti,ab OR "low molecular weight kininogens".ti,ab OR "lmwk".ti,ab OR "high molecular weight kininogens".ti,ab OR "hmwk".ti,ab OR "fitzgerald factor".ti,ab OR "plasminogen".ti,ab OR "plasminogen activator".ti,ab OR "plasminogen activator inhibitors".ti,ab OR "endothelial plasminogen activator".ti,ab OR "endothelial plasminogen activator inhibitor".ti,ab OR "pai 1".ti,ab OR "serpin e1".ti,ab OR "serpine1".ti,ab OR "serpine1 protein".ti,ab OR "type 1 plasminogen activator inhibitor".ti,ab OR "serpin b2".ti,ab OR "type 2 plasminogen activator inhibitor".ti,ab OR "pai 2".ti,ab OR "pai 3".ti,ab OR "plasminogen activator inhibitor".ti,ab OR "activated protein c inhibitor".ti,ab OR "plasminogen activator inhibitor 3".ti,ab OR "phosphorylcholine".ti,ab OR "acetyl glyceryl phosphorylcholine".ti,ab OR "aggregating factor".ti,ab OR "platelet aggregation enhancing factor".ti,ab OR "agepc".ti,ab OR "thrombocyte aggregating activity".ti,ab OR "alkyl 2 acetyl sn glycerophosphocholine".ti,ab OR "1 alkyl 2 acetyl sn glyceryl 3 phosphorylcholine".ti,ab OR "1 alkyl 2 acetylglycerophosphocholine".ti,ab OR "paf acether".ti,ab OR "platelet activating substance".ti,ab OR "platelet activating substances".ti,ab OR "pf 3".ti,ab OR "platelet factor 3".ti,ab OR "chemokine cxcl4".ti,ab OR "cxcl4".ti,ab OR "chemokine pf4".ti,ab OR "platelet factor 4".ti,ab OR "heparin neutralizing protein".ti,ab OR "antiheparin factor".ti,ab OR "thrombase".ti,ab OR "thrombostat".ti,ab OR "alpha thrombin".ti,ab OR "thrombin jmi".ti,ab OR "jmi".ti,ab OR "thrombin".ti,ab OR "beta thrombin".ti,ab OR "gamma thrombin".ti,ab OR "plasminogen activator".ti,ab OR "tissue activator d 44".ti,ab OR "tisokinase".ti,ab OR "tissue type plasminogen activator".ti,ab OR "ttpa".ti,ab OR "t plasminogen activator".ti,ab OR "tissue type activator".ti,ab OR "rt pa".ti,ab OR "factor viiir ag".ti,ab OR "factor viiir rco".ti,ab OR "ristocetin willebrand factor".ti,ab OR "von willebrand protein".ti,ab OR "von willebrand factor".ti,ab OR "factor viii related antigen".ti,ab OR "ristocetin cofactor".ti,ab OR "plasma factor viii complex".ti,ab OR "vitamin k dependent protein s".ti,ab OR "cofactor protein s".ti,ab OR "protein s".ti,ab OR "protein c".ti,ab OR "antiphospholipid".ti,ab OR "glutamate".ti,ab OR "beta 2 glycoprotein".ti,ab OR "anti beta 2 glycoprotein".ti,ab OR "adp".ti,ab OR "adenosine diphosphate".ti,ab OR "adenosine pyrophosphate".ti,ab OR "adenosine 5' pyrophosphate".ti,ab OR "serotonin".ti,ab OR "5 hydroxytryptamine".ti,ab OR "hippophaine".ti,ab OR "3 2 aminoethyl 2 methyl 1 h indol 5 ol".ti,ab OR "enteramine".ti,ab OR "5 ht".ti,ab OR "hydroxytryptamine".ti,ab OR "thromboxane a2".ti,ab OR "txa2".ti,ab OR "arachidonic acid".ti,ab OR "aa".ti,ab OR "acetylsalicylic acid".ti,ab OR "aspirin".ti,ab OR "platelet derived growth factor".ti,ab OR "pdgf receptor".ti,ab OR "pdgf".ti,ab OR "receptor tgf beta".ti,ab OR "tgfbeta".ti,ab OR "tgf beta".ti,ab OR "platelet transforming growth factor".ti,ab OR "gmp 140".ti,ab OR "p selectin".ti,ab OR "padgem".ti,ab OR "cd62p antigen".ti,ab OR "cd62p antigens".ti,ab OR "cd62p".ti,ab OR "alpha granule membrane protein".ti,ab OR "lecam3".ti,ab OR "gpvi".ti,ab OR "platelet membrane glycoprotein p62".ti,ab OR "glycoprotein gp vi".ti,ab OR "platelet glycoprotein vi".ti,ab OR "human glycoprotein vi".ti,ab OR "human alpha2beta1".ti,ab OR "integrin vla 2".ti,ab OR "vla 2".ti,ab OR "glycoprotein ia iia".ti,ab OR "alpha2beta1integrin".ti,ab OR "very late antigen 2".ti,ab OR "late antigen 2".ti,ab OR "cd49b cd29".ti,ab OR "platelet membrane glycoprotein ia/iia".ti,ab OR "platelet membrane glycoprotein ia iia".ti,ab OR "platelet membrane glycoprotein ia/iia complex".ti,ab OR "platelet membrane glycoprotein ia iia complex".ti,ab OR "platelet glycoprotein gpiib iiia complex".ti,ab OR "glycoproteins iib iiia".ti,ab OR "glycoproteins iib iiia complex".ti,ab OR "integrin alpha iib".ti,ab OR "integrin alpha iib beta 3".ti,ab OR "platelet glycoprotein gpib ix complex".ti,ab OR "platelet glycoprotein gpib ix complex".ti,ab OR "glycoprotein ib ix complex".ti,ab OR "platelet membrane glycoprotein ib".ti,ab OR "glycoprotein ib beta".ti,ab OR "cd42c".ti,ab OR "calcitonin gene related peptide".ti,ab OR "calcitonin gene related peptide ii".ti,ab OR "beta cgrp".ti,ab OR "beta calcitonin gene related peptide".ti,ab OR "alpha cgrp".ti,ab OR "alpha calcitonin gene related peptide".ti,ab OR "calcitonin gene related peptide i".ti,ab OR "cgrp".ti,ab OR "cyclo oxygenase i".ti,ab OR "cox1".ti,ab OR "prostaglandin h synthase 1".ti,ab OR "prostaglandin synthase cyclooxygenase".ti,ab OR "prostaglandin synthase".ti,ab OR "cox 1 synthesis".ti,ab OR "cox 1 prostaglandin".ti,ab OR "cyclooxygenase 1".ti,ab OR "endothelium derived vasoconstrictor factors".ti,ab OR "vasoconstrictor factors".ti,ab OR "endothelium derived endothelin 1".ti,ab OR "nitric nitrogen".ti,ab OR "endothelium derived nitric oxide".ti,ab OR "vasodilator nitrates".ti,ab OR "vasodilator nitric oxide".ti,ab OR "von willebrand factor cleaving protease".ti,ab OR "adamts13".ti,ab OR "vwf cleaving protease".ti,ab OR "vwf cleaving protease adamts13".ti,ab OR "p2y12 receptors".ti,ab OR "p2y12 receptor".ti,ab OR "p2y12".ti,ab OR "purinergic p2y12 receptors".ti,ab OR "purinergic p2y12 receptor".ti,ab OR "purinergic receptor p2y".ti,ab OR "p2y adp receptor".ti,ab OR "p2y adp receptors".ti,ab OR "p selectin glycoprotein ligand 1".ti,ab OR "p selectin".ti,ab OR CD162.ti,ab OR "platelet p selectin".ti,ab OR "psgl 1 protein".ti,ab OR "selectin P ligand protein".ti,ab OR "endocannabinoid".ti,ab OR "endocannabinoids".ti,ab OR "cb2 receptor".ti,ab OR "cannabinoid receptor cb2".ti,ab OR "cannabinoid receptor 2".ti,ab OR "cannabinoid receptor".ti,ab OR "cannabinoid cb1 receptor".ti,ab OR "cannabinoid cb2 receptor".ti,ab OR "cannabinoid receptor cb1".ti,ab OR "cannabinoid receptor 1".ti,ab OR "cb1 cannabinoid receptor".ti,ab OR "cb2 cannabinoid receptor".ti,ab OR "glyceryl 2 arachidonate".ti,ab OR "2 ag".ti,ab OR "aea".ti,ab OR "2 arachidonoylglycerol".ti,ab OR "anandamide".ti,ab OR "n arachidonoylethanolamine".ti,ab OR "metalloproteinase 9".ti,ab OR "matrix metalloproteinase 9".ti,ab OR "mmp9".ti,ab OR "metalloproteinase".ti,ab OR "metallopeptidases".ti,ab OR "metalloproteinases".ti,ab OR "metallopeptidase".ti,ab OR "type iv collagenase".ti,ab OR "type iv collagenase mmp 2".ti,ab OR "type iv collagenase mmp 9".ti,ab OR "metalloproteinase mmp 9".ti,ab OR "metalloproteinase 1".ti,ab OR "mmp1".ti,ab OR "metalloproteinase mmp1".ti,ab OR "mmp 1".ti,ab OR "mmp 9".ti,ab OR "matrix metalloproteinase 1".ti,ab OR "metalloproteinase 3".ti,ab OR "mmp3".ti,ab OR "matrix metalloproteinase 3".ti,ab OR "metalloproteinase 2".ti,ab OR "mmp2".ti,ab OR "matrix metalloproteinase 2".ti,ab OR "type iv collagenase mmp 2".ti,ab OR "mmp 2 gelatinase a".ti,ab) AND ("female".ti,ab OR "females".ti,ab OR "women".ti,ab OR "woman".ti,ab OR "girl".ti,ab OR "girls".ti,ab)) OR ((exp *"cerebrovascular accident"/ OR "stroke".ti,ab OR "cva".ti,ab OR "cerebrovascular accident".ti,ab OR "cerebrovascular accidents".ti,ab OR "cerebrovascular apoplexy".ti,ab OR "apoplexy".ti,ab OR "brain vascular accidents".ti,ab OR "vascular accidents".ti,ab OR "cerebrovascular stroke".ti,ab OR "cerebrovascular strokes".ti,ab OR "cerebrovascular apoplexy".ti,ab OR "cerebral stroke".ti,ab OR "cerebral strokes".ti,ab OR "acute stroke".ti,ab OR "acute strokes".ti,ab OR "acute cerebrovascular accident".ti,ab OR "acute cerebrovascular accidents".ti,ab OR "cerebrovascular accidents".ti,ab OR "brain infarction".ti,ab OR "brain infarctions".ti,ab OR "brain infarct".ti,ab OR "brain infarcts".ti,ab OR "anterior circulation infarction".ti,ab OR "venous infarction".ti,ab OR "venous infarctions".ti,ab OR "posterior circulation infarction".ti,ab OR "posterior circulation infarcts".ti,ab OR "brain ischemia".ti,ab OR "ischemic encephalopathy".ti,ab OR "ischemic encephalopathies".ti,ab OR "cerebral ischemia".ti,ab OR "cerebral ischemias".ti,ab) AND ("blood coagulation".ti,ab OR "blood coagulation factor".ti,ab OR "blood coagulation factors".ti,ab OR "coagulation factor".ti,ab OR "coagulation factors".ti,ab OR "clotting factor".ti,ab OR "clotting factors".ti,ab OR "blood clotting".ti,ab OR "blood clotting factor".ti,ab OR "blood clotting factors".ti,ab OR "thromboses".ti,ab OR "thrombus".ti,ab OR "blood clot".ti,ab OR "blood clots".ti,ab OR "thrombus formation".ti,ab OR "hemostases".ti,ab OR "platelet activation".ti,ab OR "platelet activations".ti,ab OR "platelet aggregation".ti,ab OR "platelet clotting".ti,ab OR "platelet adhesion".ti,ab OR exp *"Blood Clotting Factor"/ OR "factor i".ti,ab OR "blood coagulation factor i".ti,ab OR "coagulation factor i".ti,ab OR "fibrinogen".ti,ab OR "gamma fibrinogen".ti,ab OR "gamma fibrinogen".ti,ab OR "fi".ti,ab OR "factor ii".ti,ab OR "blood coagulation factor ii".ti,ab OR "differentiation reversal factor".ti,ab OR "coagulation factor ii".ti,ab OR "prothrombin".ti,ab OR "fii".ti,ab OR "thromboplastin".ti,ab OR "coagulin".ti,ab OR "factor iii".ti,ab OR "fiii".ti,ab OR "coagulation factor iii".ti,ab OR "coagulation tissue factor".ti,ab OR "tissue thromboplastin".ti,ab OR "thromboplastin".ti,ab OR "procoagulant".ti,ab OR "tissue factor procoagulant".ti,ab OR "coagulation factor iv".ti,ab OR "factor iv".ti,ab OR "calcium 40".ti,ab OR "calcium".ti,ab OR "fv".ti,ab OR "factor five".ti,ab OR "blood coagulation factor v".ti,ab OR "coagulation factor v".ti,ab OR "coagulation factor v".ti,ab OR "factor v".ti,ab OR "activated factor v".ti,ab OR "coagulation factor va".ti,ab OR "factor va".ti,ab OR "factor v leiden".ti,ab OR "factor five leiden".ti,ab OR "factor seven".ti,ab OR "coagulation factor vii".ti,ab OR "factor vii".ti,ab OR "blood coagulation factor vii".ti,ab OR "proconvertin".ti,ab OR "coagulation factor 7".ti,ab OR "factor 7".ti,ab OR "coagulation factor viia".ti,ab OR "factor viia".ti,ab OR "activated factor vii".ti,ab OR "activated factor vii".ti,ab OR "factor 7a".ti,ab OR "factor 8".ti,ab OR "coagulation factor viii".ti,ab OR "thromboplastinogen".ti,ab OR "blood coagulation factor viii".ti,ab OR "factor eight".ti,ab OR "hyatt c".ti,ab OR "factor viiic".ti,ab OR "f viii c".ti,ab OR "factor viii".ti,ab OR "blood coagulation factor viii".ti,ab OR "coagulation factor viiia".ti,ab OR "factor viiia".ti,ab OR "coagulation factor viii".ti,ab OR "thrombin activated factor viii".ti,ab OR "factor 8a".ti,ab OR "activated factor viii".ti,ab OR "factor nine".ti,ab OR "autoprothrombin ii".ti,ab OR "christmas factor".ti,ab OR "factor ix".ti,ab OR "blood coagulation factor ix".ti,ab OR "factor ix complex".ti,ab OR "factor 9".ti,ab OR "coagulation factor ix".ti,ab OR "coagulation factor ixa".ti,ab OR "factor ixa".ti,ab OR "activated factor ix".ti,ab OR "factor 9a".ti,ab OR "plasma thromboplastin component".ti,ab OR "factor ten".ti,ab OR "autoprothrombin iii".ti,ab OR "coagulation factor x".ti,ab OR "factor x".ti,ab OR "blood coagulation factor x".ti,ab OR "stuart factor".ti,ab OR "stuart prower factor".ti,ab OR "factor 10".ti,ab OR "activated factor x".ti,ab OR "activated coagulation factor x".ti,ab OR "factor xa".ti,ab OR "blood coagulation factor x".ti,ab OR "factor 10a".ti,ab OR "autoprothrombin c".ti,ab OR "factor eleven".ti,ab OR "blood coagulation factor xi".ti,ab OR "plasma thromboplastin".ti,ab OR "plasma thromboplastin antecedent".ti,ab OR "antecedent".ti,ab OR "plasma thromboplastin".ti,ab OR "thromboplastin antecedent".ti,ab OR "coagulation factor xi".ti,ab OR "factor xi".ti,ab OR "coagulation factor 11".ti,ab OR "factor 11".ti,ab OR "activated factor xi".ti,ab OR "coagulation factor xia".ti,ab OR "factor xia".ti,ab OR "blood coagulation factor xi".ti,ab OR "factor 11a".ti,ab OR "factor twelve".ti,ab OR "hageman factor".ti,ab OR "factor 12".ti,ab OR "coagulation factor xii".ti,ab OR "activated factor xii".ti,ab OR "coagulation factor xiia".ti,ab OR "factor xiia".ti,ab OR "blood coagulation factor xii".ti,ab OR "activated prekallikrein".ti,ab OR "prekallikrein".ti,ab OR "factor xii".ti,ab OR "activated hageman factor".ti,ab OR "hageman factor fragment".ti,ab OR "factor thirteen".ti,ab OR "coagulation factor xiii".ti,ab OR "factor xiii".ti,ab OR "factor xiii".ti,ab OR "transamidase".ti,ab OR "fibrinase".ti,ab OR "laki lorand factor".ti,ab OR "blood coagulation factor xiii".ti,ab OR "factor 13".ti,ab OR "fibrin stabilizing factor".ti,ab OR "stabilizing factor".ti,ab OR "activated factor xiii".ti,ab OR "factor 13a".ti,ab OR "plasma transglutaminase".ti,ab OR "transglutaminase".ti,ab OR "beta thromboglobulin".ti,ab OR "beta 2 thromboglobulin".ti,ab OR "thromboglobulin".ti,ab OR "fibrinopeptides a".ti,ab OR "fibrinopeptides b".ti,ab OR "kalliginogenase".ti,ab OR "kallikrein".ti,ab OR "kallikrein kininogenase".ti,ab OR "callicrein".ti,ab OR "kinin forming enzyme".ti,ab OR "kinin forming".ti,ab OR "kallikrein light chain".ti,ab OR "kallikrein padutin".ti,ab OR "kallikrein a".ti,ab OR "beta kallikrein".ti,ab OR "kallikrein b".ti,ab OR "alpha kallikrein".ti,ab OR "plasma kallikrein".ti,ab OR "kininogenin".ti,ab OR "kallikrein i".ti,ab OR "plasma prokallikrein".ti,ab OR "prokallikrein".ti,ab OR "prokinins".ti,ab OR "cystatins".ti,ab OR "t kininogen".ti,ab OR "low molecular weight kininogens".ti,ab OR "lmwk".ti,ab OR "high molecular weight kininogens".ti,ab OR "hmwk".ti,ab OR "fitzgerald factor".ti,ab OR "plasminogen".ti,ab OR "plasminogen activator".ti,ab OR "plasminogen activator inhibitors".ti,ab OR "endothelial plasminogen activator".ti,ab OR "endothelial plasminogen activator inhibitor".ti,ab OR "pai 1".ti,ab OR "serpin e1".ti,ab OR "serpine1".ti,ab OR "serpine1 protein".ti,ab OR "type 1 plasminogen activator inhibitor".ti,ab OR "serpin b2".ti,ab OR "type 2 plasminogen activator inhibitor".ti,ab OR "pai 2".ti,ab OR "pai 3".ti,ab OR "plasminogen activator inhibitor".ti,ab OR "activated protein c inhibitor".ti,ab OR "plasminogen activator inhibitor 3".ti,ab OR "phosphorylcholine".ti,ab OR "acetyl glyceryl phosphorylcholine".ti,ab OR "aggregating factor".ti,ab OR "platelet aggregation enhancing factor".ti,ab OR "agepc".ti,ab OR "thrombocyte aggregating activity".ti,ab OR "alkyl 2 acetyl sn glycerophosphocholine".ti,ab OR "1 alkyl 2 acetyl sn glyceryl 3 phosphorylcholine".ti,ab OR "1 alkyl 2 acetylglycerophosphocholine".ti,ab OR "paf acether".ti,ab OR "platelet activating substance".ti,ab OR "platelet activating substances".ti,ab OR "pf 3".ti,ab OR "platelet factor 3".ti,ab OR "chemokine cxcl4".ti,ab OR "cxcl4".ti,ab OR "chemokine pf4".ti,ab OR "platelet factor 4".ti,ab OR "heparin neutralizing protein".ti,ab OR "antiheparin factor".ti,ab OR "thrombase".ti,ab OR "thrombostat".ti,ab OR "alpha thrombin".ti,ab OR "thrombin jmi".ti,ab OR "jmi".ti,ab OR "thrombin".ti,ab OR "beta thrombin".ti,ab OR "gamma thrombin".ti,ab OR "plasminogen activator".ti,ab OR "tissue activator d 44".ti,ab OR "tisokinase".ti,ab OR "tissue type plasminogen activator".ti,ab OR "ttpa".ti,ab OR "t plasminogen activator".ti,ab OR "tissue type activator".ti,ab OR "rt pa".ti,ab OR "factor viiir ag".ti,ab OR "factor viiir rco".ti,ab OR "ristocetin willebrand factor".ti,ab OR "von willebrand protein".ti,ab OR "von willebrand factor".ti,ab OR "factor viii related antigen".ti,ab OR "ristocetin cofactor".ti,ab OR "plasma factor viii complex".ti,ab OR "vitamin k dependent protein s".ti,ab OR "cofactor protein s".ti,ab OR "protein s".ti,ab OR "protein c".ti,ab OR "antiphospholipid".ti,ab OR "glutamate".ti,ab OR "beta 2 glycoprotein".ti,ab OR "anti beta 2 glycoprotein".ti,ab OR "adp".ti,ab OR "adenosine diphosphate".ti,ab OR "adenosine pyrophosphate".ti,ab OR "adenosine 5' pyrophosphate".ti,ab OR "serotonin".ti,ab OR "5 hydroxytryptamine".ti,ab OR "hippophaine".ti,ab OR "3 2 aminoethyl 2 methyl 1 h indol 5 ol".ti,ab OR "enteramine".ti,ab OR "5 ht".ti,ab OR "hydroxytryptamine".ti,ab OR "thromboxane a2".ti,ab OR "txa2".ti,ab OR "arachidonic acid".ti,ab OR "aa".ti,ab OR "acetylsalicylic acid".ti,ab OR "aspirin".ti,ab OR "platelet derived growth factor".ti,ab OR "pdgf receptor".ti,ab OR "pdgf".ti,ab OR "receptor tgf beta".ti,ab OR "tgfbeta".ti,ab OR "tgf beta".ti,ab OR "platelet transforming growth factor".ti,ab OR "gmp 140".ti,ab OR "p selectin".ti,ab OR "padgem".ti,ab OR "cd62p antigen".ti,ab OR "cd62p antigens".ti,ab OR "cd62p".ti,ab OR "alpha granule membrane protein".ti,ab OR "lecam3".ti,ab OR "gpvi".ti,ab OR "platelet membrane glycoprotein p62".ti,ab OR "glycoprotein gp vi".ti,ab OR "platelet glycoprotein vi".ti,ab OR "human glycoprotein vi".ti,ab OR "human alpha2beta1".ti,ab OR "integrin vla 2".ti,ab OR "vla 2".ti,ab OR "glycoprotein ia iia".ti,ab OR "alpha2beta1integrin".ti,ab OR "very late antigen 2".ti,ab OR "late antigen 2".ti,ab OR "cd49b cd29".ti,ab OR "platelet membrane glycoprotein ia/iia".ti,ab OR "platelet membrane glycoprotein ia iia".ti,ab OR "platelet membrane glycoprotein ia/iia complex".ti,ab OR "platelet membrane glycoprotein ia iia complex".ti,ab OR "platelet glycoprotein gpiib iiia complex".ti,ab OR "glycoproteins iib iiia".ti,ab OR "glycoproteins iib iiia complex".ti,ab OR "integrin alpha iib".ti,ab OR "integrin alpha iib beta 3".ti,ab OR "platelet glycoprotein gpib ix complex".ti,ab OR "platelet glycoprotein gpib ix complex".ti,ab OR "glycoprotein ib ix complex".ti,ab OR "platelet membrane glycoprotein ib".ti,ab OR "glycoprotein ib beta".ti,ab OR "cd42c".ti,ab OR "calcitonin gene related peptide".ti,ab OR "calcitonin gene related peptide ii".ti,ab OR "beta cgrp".ti,ab OR "beta calcitonin gene related peptide".ti,ab OR "alpha cgrp".ti,ab OR "alpha calcitonin gene related peptide".ti,ab OR "calcitonin gene related peptide i".ti,ab OR "cgrp".ti,ab OR "cyclo oxygenase i".ti,ab OR "cox1".ti,ab OR "prostaglandin h synthase 1".ti,ab OR "prostaglandin synthase cyclooxygenase".ti,ab OR "prostaglandin synthase".ti,ab OR "cox 1 synthesis".ti,ab OR "cox 1 prostaglandin".ti,ab OR "cyclooxygenase 1".ti,ab OR "endothelium derived vasoconstrictor factors".ti,ab OR "vasoconstrictor factors".ti,ab OR "endothelium derived endothelin 1".ti,ab OR "nitric nitrogen".ti,ab OR "endothelium derived nitric oxide".ti,ab OR "vasodilator nitrates".ti,ab OR "vasodilator nitric oxide".ti,ab OR "von willebrand factor cleaving protease".ti,ab OR "adamts13".ti,ab OR "vwf cleaving protease".ti,ab OR "vwf cleaving protease adamts13".ti,ab OR "p2y12 receptors".ti,ab OR "p2y12 receptor".ti,ab OR "p2y12".ti,ab OR "purinergic p2y12 receptors".ti,ab OR "purinergic p2y12 receptor".ti,ab OR "purinergic receptor p2y".ti,ab OR "p2y adp receptor".ti,ab OR "p2y adp receptors".ti,ab OR "p selectin glycoprotein ligand 1".ti,ab OR "p selectin".ti,ab OR CD162.ti,ab OR "platelet p selectin".ti,ab OR "psgl 1 protein".ti,ab OR "selectin P ligand protein".ti,ab OR "endocannabinoid".ti,ab OR "endocannabinoids".ti,ab OR "cb2 receptor".ti,ab OR "cannabinoid receptor cb2".ti,ab OR "cannabinoid receptor 2".ti,ab OR "cannabinoid receptor".ti,ab OR "cannabinoid cb1 receptor".ti,ab OR "cannabinoid cb2 receptor".ti,ab OR "cannabinoid receptor cb1".ti,ab OR "cannabinoid receptor 1".ti,ab OR "cb1 cannabinoid receptor".ti,ab OR "cb2 cannabinoid receptor".ti,ab OR "glyceryl 2 arachidonate".ti,ab OR "2 ag".ti,ab OR "aea".ti,ab OR "2 arachidonoylglycerol".ti,ab OR "anandamide".ti,ab OR "n arachidonoylethanolamine".ti,ab OR "metalloproteinase 9".ti,ab OR "matrix metalloproteinase 9".ti,ab OR "mmp9".ti,ab OR "metalloproteinase".ti,ab OR "metallopeptidases".ti,ab OR "metalloproteinases".ti,ab OR "metallopeptidase".ti,ab OR "type iv collagenase".ti,ab OR "type iv collagenase mmp 2".ti,ab OR "type iv collagenase mmp 9".ti,ab OR "metalloproteinase mmp 9".ti,ab OR "metalloproteinase 1".ti,ab OR "mmp1".ti,ab OR "metalloproteinase mmp1".ti,ab OR "mmp 1".ti,ab OR "mmp 9".ti,ab OR "matrix metalloproteinase 1".ti,ab OR "metalloproteinase 3".ti,ab OR "mmp3".ti,ab OR "matrix metalloproteinase 3".ti,ab OR "metalloproteinase 2".ti,ab OR "mmp2".ti,ab OR "matrix metalloproteinase 2".ti,ab OR "type iv collagenase mmp 2".ti,ab OR "mmp 2 gelatinase a".ti,ab) AND ("female".ti,ab OR "females".ti,ab OR "women".ti,ab OR "woman".ti,ab OR "girl".ti,ab OR "girls".ti,ab))) NOT ("Tissue Plasminogen Activator"/pd OR "Aspirin"/pd OR exp "cerebrovascular accident"/th) NOT (("Case Report"/ OR "case report".ti OR exp "Review"/ OR "review".ti OR "systematic review"/ OR "systematic review".ti OR exp "meta analysis"/ OR "meta-analysis".ti) NOT ("Clinical Study"/ OR exp "Clinical Trial"/ OR "trial".ti OR "RCT".ti)) NOT (exp *"cerebrovascular accident"/th OR therap*.ti OR treat*.ti) AND (english.la OR dutch.la) NOT ((exp "Child"/ OR exp "Infant"/) NOT (exp "Adult"/)) NOT (conference abstract OR conference review).pt

**Web of Science**

((ts=("cerebrovascular accident" OR "stroke" OR "cva" OR "cerebrovascular accident" OR "cerebrovascular accidents" OR "cerebrovascular apoplexy" OR "apoplexy" OR "brain vascular accidents" OR "vascular accidents" OR "cerebrovascular stroke" OR "cerebrovascular strokes" OR "cerebrovascular apoplexy" OR "cerebral stroke" OR "cerebral strokes" OR "acute stroke" OR "acute strokes" OR "acute cerebrovascular accident" OR "acute cerebrovascular accidents" OR "cerebrovascular accidents" OR "brain infarction" OR "brain infarctions" OR "brain infarct" OR "brain infarcts" OR "anterior circulation infarction" OR "venous infarction" OR "venous infarctions" OR "posterior circulation infarction" OR "posterior circulation infarcts" OR "brain ischemia" OR "ischemic encephalopathy" OR "ischemic encephalopathies" OR "cerebral ischemia" OR "cerebral ischemias") AND ts=("Migraine" OR "migraine" OR "migraine disorder" OR "migraine disorders" OR "migraines" OR "migraine headache" OR "migraine headaches" OR "headache" OR "headaches" OR "acute confusional migraine" OR "migraine hemicrania" OR "migraine variants" OR "migraine variant" OR "common migraine" OR "common migraines" OR "familial hemiplegic migraine" OR "familial hemiplegic migraines" OR "hemiplegic migraine" OR "classical migraine" OR "classical migraine attacks" OR "migraine with aura" OR "migraine with auras" OR "prolonged aura migraine" OR "migraineur" OR "migraineurs" OR "migraineurs without aura" OR "migrainous attack" OR "migrainous attacks" OR "migrainous aura" OR "migrainous auras" OR migrain*) AND ts=("blood coagulation" OR "blood coagulation factor" OR "blood coagulation factors" OR "coagulation factor" OR "coagulation factors" OR "clotting factor" OR "clotting factors" OR "blood clotting" OR "blood clotting factor" OR "blood clotting factors" OR "thromboses" OR "thrombus" OR "blood clot" OR "blood clots" OR "thrombus formation" OR "hemostases" OR "platelet activation" OR "platelet activations" OR "platelet aggregation" OR "platelet clotting" OR "platelet adhesion" OR "Blood Clotting Factor" OR "factor i" OR "blood coagulation factor i" OR "coagulation factor i" OR "fibrinogen" OR "gamma fibrinogen" OR "gamma fibrinogen" OR "fi" OR "factor ii" OR "blood coagulation factor ii" OR "differentiation reversal factor" OR "coagulation factor ii" OR "prothrombin" OR "fii" OR "thromboplastin" OR "coagulin" OR "factor iii" OR "fiii" OR "coagulation factor iii" OR "coagulation tissue factor" OR "tissue thromboplastin" OR "thromboplastin" OR "procoagulant" OR "tissue factor procoagulant" OR "coagulation factor iv" OR "factor iv" OR "calcium 40" OR "calcium" OR "fv" OR "factor five" OR "blood coagulation factor v" OR "coagulation factor v" OR "coagulation factor v" OR "factor v" OR "activated factor v" OR "coagulation factor va" OR "factor va" OR "factor v leiden" OR "factor five leiden" OR "factor seven" OR "coagulation factor vii" OR "factor vii" OR "blood coagulation factor vii" OR "proconvertin" OR "coagulation factor 7" OR "factor 7" OR "coagulation factor viia" OR "factor viia" OR "activated factor vii" OR "activated factor vii" OR "factor 7a" OR "factor 8" OR "coagulation factor viii" OR "thromboplastinogen" OR "blood coagulation factor viii" OR "factor eight" OR "hyatt c" OR "factor viiic" OR "f viii c" OR "factor viii" OR "blood coagulation factor viii" OR "coagulation factor viiia" OR "factor viiia" OR "coagulation factor viii" OR "thrombin activated factor viii" OR "factor 8a" OR "activated factor viii" OR "factor nine" OR "autoprothrombin ii" OR "christmas factor" OR "factor ix" OR "blood coagulation factor ix" OR "factor ix complex" OR "factor 9" OR "coagulation factor ix" OR "coagulation factor ixa" OR "factor ixa" OR "activated factor ix" OR "factor 9a" OR "plasma thromboplastin component" OR "factor ten" OR "autoprothrombin iii" OR "coagulation factor x" OR "factor x" OR "blood coagulation factor x" OR "stuart factor" OR "stuart prower factor" OR "factor 10" OR "activated factor x" OR "activated coagulation factor x" OR "factor xa" OR "blood coagulation factor x" OR "factor 10a" OR "autoprothrombin c" OR "factor eleven" OR "blood coagulation factor xi" OR "plasma thromboplastin" OR "plasma thromboplastin antecedent" OR "antecedent" OR "plasma thromboplastin" OR "thromboplastin antecedent" OR "coagulation factor xi" OR "factor xi" OR "coagulation factor 11" OR "factor 11" OR "activated factor xi" OR "coagulation factor xia" OR "factor xia" OR "blood coagulation factor xi" OR "factor 11a" OR "factor twelve" OR "hageman factor" OR "factor 12" OR "coagulation factor xii" OR "activated factor xii" OR "coagulation factor xiia" OR "factor xiia" OR "blood coagulation factor xii" OR "activated prekallikrein" OR "prekallikrein" OR "factor xii" OR "activated hageman factor" OR "hageman factor fragment" OR "factor thirteen" OR "coagulation factor xiii" OR "factor xiii" OR "factor xiii" OR "transamidase" OR "fibrinase" OR "laki lorand factor" OR "blood coagulation factor xiii" OR "factor 13" OR "fibrin stabilizing factor" OR "stabilizing factor" OR "activated factor xiii" OR "factor 13a" OR "plasma transglutaminase" OR "transglutaminase" OR "beta thromboglobulin" OR "beta 2 thromboglobulin" OR "thromboglobulin" OR "fibrinopeptides a" OR "fibrinopeptides b" OR "kalliginogenase" OR "kallikrein" OR "kallikrein kininogenase" OR "callicrein" OR "kinin forming enzyme" OR "kinin forming" OR "kallikrein light chain" OR "kallikrein padutin" OR "kallikrein a" OR "beta kallikrein" OR "kallikrein b" OR "alpha kallikrein" OR "plasma kallikrein" OR "kininogenin" OR "kallikrein i" OR "plasma prokallikrein" OR "prokallikrein" OR "prokinins" OR "cystatins" OR "t kininogen" OR "low molecular weight kininogens" OR "lmwk" OR "high molecular weight kininogens" OR "hmwk" OR "fitzgerald factor" OR "plasminogen" OR "plasminogen activator" OR "plasminogen activator inhibitors" OR "endothelial plasminogen activator" OR "endothelial plasminogen activator inhibitor" OR "pai 1" OR "serpin e1" OR "serpine1" OR "serpine1 protein" OR "type 1 plasminogen activator inhibitor" OR "serpin b2" OR "type 2 plasminogen activator inhibitor" OR "pai 2" OR "pai 3" OR "plasminogen activator inhibitor" OR "activated protein c inhibitor" OR "plasminogen activator inhibitor 3" OR "phosphorylcholine" OR "acetyl glyceryl phosphorylcholine" OR "aggregating factor" OR "platelet aggregation enhancing factor" OR "agepc" OR "thrombocyte aggregating activity" OR "alkyl 2 acetyl sn glycerophosphocholine" OR "1 alkyl 2 acetyl sn glyceryl 3 phosphorylcholine" OR "1 alkyl 2 acetylglycerophosphocholine" OR "paf acether" OR "platelet activating substance" OR "platelet activating substances" OR "pf 3" OR "platelet factor 3" OR "chemokine cxcl4" OR "cxcl4" OR "chemokine pf4" OR "platelet factor 4" OR "heparin neutralizing protein" OR "antiheparin factor" OR "thrombase" OR "thrombostat" OR "alpha thrombin" OR "thrombin jmi" OR "jmi" OR "thrombin" OR "beta thrombin" OR "gamma thrombin" OR "plasminogen activator" OR "tissue activator d 44" OR "tisokinase" OR "tissue type plasminogen activator" OR "ttpa" OR "t plasminogen activator" OR "tissue type activator" OR "rt pa" OR "factor viiir ag" OR "factor viiir rco" OR "ristocetin willebrand factor" OR "von willebrand protein" OR "von willebrand factor" OR "factor viii related antigen" OR "ristocetin cofactor" OR "plasma factor viii complex" OR "vitamin k dependent protein s" OR "cofactor protein s" OR "protein s" OR "protein c" OR "antiphospholipid" OR "glutamate" OR "beta 2 glycoprotein" OR "anti beta 2 glycoprotein" OR "adp" OR "adenosine diphosphate" OR "adenosine pyrophosphate" OR "adenosine 5' pyrophosphate" OR "serotonin" OR "5 hydroxytryptamine" OR "hippophaine" OR "3 2 aminoethyl 2 methyl 1 h indol 5 ol" OR "enteramine" OR "5 ht" OR "hydroxytryptamine" OR "thromboxane a2" OR "txa2" OR "arachidonic acid" OR "aa" OR "acetylsalicylic acid" OR "aspirin" OR "platelet derived growth factor" OR "pdgf receptor" OR "pdgf" OR "receptor tgf beta" OR "tgfbeta" OR "tgf beta" OR "platelet transforming growth factor" OR "gmp 140" OR "p selectin" OR "padgem" OR "cd62p antigen" OR "cd62p antigens" OR "cd62p" OR "alpha granule membrane protein" OR "lecam3" OR "gpvi" OR "platelet membrane glycoprotein p62" OR "glycoprotein gp vi" OR "platelet glycoprotein vi" OR "human glycoprotein vi" OR "human alpha2beta1" OR "integrin vla 2" OR "vla 2" OR "glycoprotein ia iia" OR "alpha2beta1integrin" OR "very late antigen 2" OR "late antigen 2" OR "cd49b cd29" OR "platelet membrane glycoprotein iaiia" OR "platelet membrane glycoprotein ia iia" OR "platelet membrane glycoprotein iaiia complex" OR "platelet membrane glycoprotein ia iia complex" OR "platelet glycoprotein gpiib iiia complex" OR "glycoproteins iib iiia" OR "glycoproteins iib iiia complex" OR "integrin alpha iib" OR "integrin alpha iib beta 3" OR "platelet glycoprotein gpib ix complex" OR "platelet glycoprotein gpib ix complex" OR "glycoprotein ib ix complex" OR "platelet membrane glycoprotein ib" OR "glycoprotein ib beta" OR "cd42c" OR "calcitonin gene related peptide" OR "calcitonin gene related peptide ii" OR "beta cgrp" OR "beta calcitonin gene related peptide" OR "alpha cgrp" OR "alpha calcitonin gene related peptide" OR "calcitonin gene related peptide i" OR "cgrp" OR "cyclo oxygenase i" OR "cox1" OR "prostaglandin h synthase 1" OR "prostaglandin synthase cyclooxygenase" OR "prostaglandin synthase" OR "cox 1 synthesis" OR "cox 1 prostaglandin" OR "cyclooxygenase 1" OR "endothelium derived vasoconstrictor factors" OR "vasoconstrictor factors" OR "endothelium derived endothelin 1" OR "nitric nitrogen" OR "endothelium derived nitric oxide" OR "vasodilator nitrates" OR "vasodilator nitric oxide" OR "von willebrand factor cleaving protease" OR "adamts13" OR "vwf cleaving protease" OR "vwf cleaving protease adamts13" OR "p2y12 receptors" OR "p2y12 receptor" OR "p2y12" OR "purinergic p2y12 receptors" OR "purinergic p2y12 receptor" OR "purinergic receptor p2y" OR "p2y adp receptor" OR "p2y adp receptors" OR "p selectin glycoprotein ligand 1" OR "p selectin" OR CD162 OR "platelet p selectin" OR "psgl 1 protein" OR "selectin P ligand protein" OR "endocannabinoid" OR "endocannabinoids" OR "cb2 receptor" OR "cannabinoid receptor cb2" OR "cannabinoid receptor 2" OR "cannabinoid receptor" OR "cannabinoid cb1 receptor" OR "cannabinoid cb2 receptor" OR "cannabinoid receptor cb1" OR "cannabinoid receptor 1" OR "cb1 cannabinoid receptor" OR "cb2 cannabinoid receptor" OR "glyceryl 2 arachidonate" OR "2 ag" OR "aea" OR "2 arachidonoylglycerol" OR "anandamide" OR "n arachidonoylethanolamine" OR "metalloproteinase 9" OR "matrix metalloproteinase 9" OR "mmp9" OR "metalloproteinase" OR "metallopeptidases" OR "metalloproteinases" OR "metallopeptidase" OR "type iv collagenase" OR "type iv collagenase mmp 2" OR "type iv collagenase mmp 9" OR "metalloproteinase mmp 9" OR "metalloproteinase 1" OR "mmp1" OR "metalloproteinase mmp1" OR "mmp 1" OR "mmp 9" OR "matrix metalloproteinase 1" OR "metalloproteinase 3" OR "mmp3" OR "matrix metalloproteinase 3" OR "metalloproteinase 2" OR "mmp2" OR "matrix metalloproteinase 2" OR "type iv collagenase mmp 2" OR "mmp 2 gelatinase a") AND ts=("female" OR "females" OR "women" OR "woman" OR "girl" OR "girls")) OR (ti=("cerebrovascular accident" OR "stroke" OR "cva" OR "cerebrovascular accident" OR "cerebrovascular accidents" OR "cerebrovascular apoplexy" OR "apoplexy" OR "brain vascular accidents" OR "vascular accidents" OR "cerebrovascular stroke" OR "cerebrovascular strokes" OR "cerebrovascular apoplexy" OR "cerebral stroke" OR "cerebral strokes" OR "acute stroke" OR "acute strokes" OR "acute cerebrovascular accident" OR "acute cerebrovascular accidents" OR "cerebrovascular accidents" OR "brain infarction" OR "brain infarctions" OR "brain infarct" OR "brain infarcts" OR "anterior circulation infarction" OR "venous infarction" OR "venous infarctions" OR "posterior circulation infarction" OR "posterior circulation infarcts" OR "brain ischemia" OR "ischemic encephalopathy" OR "ischemic encephalopathies" OR "cerebral ischemia" OR "cerebral ischemias") AND ti=("blood coagulation" OR "blood coagulation factor" OR "blood coagulation factors" OR "coagulation factor" OR "coagulation factors" OR "clotting factor" OR "clotting factors" OR "blood clotting" OR "blood clotting factor" OR "blood clotting factors" OR "thromboses" OR "thrombus" OR "blood clot" OR "blood clots" OR "thrombus formation" OR "hemostases" OR "platelet activation" OR "platelet activations" OR "platelet aggregation" OR "platelet clotting" OR "platelet adhesion" OR "Blood Clotting Factor" OR "factor i" OR "blood coagulation factor i" OR "coagulation factor i" OR "fibrinogen" OR "gamma fibrinogen" OR "gamma fibrinogen" OR "fi" OR "factor ii" OR "blood coagulation factor ii" OR "differentiation reversal factor" OR "coagulation factor ii" OR "prothrombin" OR "fii" OR "thromboplastin" OR "coagulin" OR "factor iii" OR "fiii" OR "coagulation factor iii" OR "coagulation tissue factor" OR "tissue thromboplastin" OR "thromboplastin" OR "procoagulant" OR "tissue factor procoagulant" OR "coagulation factor iv" OR "factor iv" OR "calcium 40" OR "calcium" OR "fv" OR "factor five" OR "blood coagulation factor v" OR "coagulation factor v" OR "coagulation factor v" OR "factor v" OR "activated factor v" OR "coagulation factor va" OR "factor va" OR "factor v leiden" OR "factor five leiden" OR "factor seven" OR "coagulation factor vii" OR "factor vii" OR "blood coagulation factor vii" OR "proconvertin" OR "coagulation factor 7" OR "factor 7" OR "coagulation factor viia" OR "factor viia" OR "activated factor vii" OR "activated factor vii" OR "factor 7a" OR "factor 8" OR "coagulation factor viii" OR "thromboplastinogen" OR "blood coagulation factor viii" OR "factor eight" OR "hyatt c" OR "factor viiic" OR "f viii c" OR "factor viii" OR "blood coagulation factor viii" OR "coagulation factor viiia" OR "factor viiia" OR "coagulation factor viii" OR "thrombin activated factor viii" OR "factor 8a" OR "activated factor viii" OR "factor nine" OR "autoprothrombin ii" OR "christmas factor" OR "factor ix" OR "blood coagulation factor ix" OR "factor ix complex" OR "factor 9" OR "coagulation factor ix" OR "coagulation factor ixa" OR "factor ixa" OR "activated factor ix" OR "factor 9a" OR "plasma thromboplastin component" OR "factor ten" OR "autoprothrombin iii" OR "coagulation factor x" OR "factor x" OR "blood coagulation factor x" OR "stuart factor" OR "stuart prower factor" OR "factor 10" OR "activated factor x" OR "activated coagulation factor x" OR "factor xa" OR "blood coagulation factor x" OR "factor 10a" OR "autoprothrombin c" OR "factor eleven" OR "blood coagulation factor xi" OR "plasma thromboplastin" OR "plasma thromboplastin antecedent" OR "antecedent" OR "plasma thromboplastin" OR "thromboplastin antecedent" OR "coagulation factor xi" OR "factor xi" OR "coagulation factor 11" OR "factor 11" OR "activated factor xi" OR "coagulation factor xia" OR "factor xia" OR "blood coagulation factor xi" OR "factor 11a" OR "factor twelve" OR "hageman factor" OR "factor 12" OR "coagulation factor xii" OR "activated factor xii" OR "coagulation factor xiia" OR "factor xiia" OR "blood coagulation factor xii" OR "activated prekallikrein" OR "prekallikrein" OR "factor xii" OR "activated hageman factor" OR "hageman factor fragment" OR "factor thirteen" OR "coagulation factor xiii" OR "factor xiii" OR "factor xiii" OR "transamidase" OR "fibrinase" OR "laki lorand factor" OR "blood coagulation factor xiii" OR "factor 13" OR "fibrin stabilizing factor" OR "stabilizing factor" OR "activated factor xiii" OR "factor 13a" OR "plasma transglutaminase" OR "transglutaminase" OR "beta thromboglobulin" OR "beta 2 thromboglobulin" OR "thromboglobulin" OR "fibrinopeptides a" OR "fibrinopeptides b" OR "kalliginogenase" OR "kallikrein" OR "kallikrein kininogenase" OR "callicrein" OR "kinin forming enzyme" OR "kinin forming" OR "kallikrein light chain" OR "kallikrein padutin" OR "kallikrein a" OR "beta kallikrein" OR "kallikrein b" OR "alpha kallikrein" OR "plasma kallikrein" OR "kininogenin" OR "kallikrein i" OR "plasma prokallikrein" OR "prokallikrein" OR "prokinins" OR "cystatins" OR "t kininogen" OR "low molecular weight kininogens" OR "lmwk" OR "high molecular weight kininogens" OR "hmwk" OR "fitzgerald factor" OR "plasminogen" OR "plasminogen activator" OR "plasminogen activator inhibitors" OR "endothelial plasminogen activator" OR "endothelial plasminogen activator inhibitor" OR "pai 1" OR "serpin e1" OR "serpine1" OR "serpine1 protein" OR "type 1 plasminogen activator inhibitor" OR "serpin b2" OR "type 2 plasminogen activator inhibitor" OR "pai 2" OR "pai 3" OR "plasminogen activator inhibitor" OR "activated protein c inhibitor" OR "plasminogen activator inhibitor 3" OR "phosphorylcholine" OR "acetyl glyceryl phosphorylcholine" OR "aggregating factor" OR "platelet aggregation enhancing factor" OR "agepc" OR "thrombocyte aggregating activity" OR "alkyl 2 acetyl sn glycerophosphocholine" OR "1 alkyl 2 acetyl sn glyceryl 3 phosphorylcholine" OR "1 alkyl 2 acetylglycerophosphocholine" OR "paf acether" OR "platelet activating substance" OR "platelet activating substances" OR "pf 3" OR "platelet factor 3" OR "chemokine cxcl4" OR "cxcl4" OR "chemokine pf4" OR "platelet factor 4" OR "heparin neutralizing protein" OR "antiheparin factor" OR "thrombase" OR "thrombostat" OR "alpha thrombin" OR "thrombin jmi" OR "jmi" OR "thrombin" OR "beta thrombin" OR "gamma thrombin" OR "plasminogen activator" OR "tissue activator d 44" OR "tisokinase" OR "tissue type plasminogen activator" OR "ttpa" OR "t plasminogen activator" OR "tissue type activator" OR "rt pa" OR "factor viiir ag" OR "factor viiir rco" OR "ristocetin willebrand factor" OR "von willebrand protein" OR "von willebrand factor" OR "factor viii related antigen" OR "ristocetin cofactor" OR "plasma factor viii complex" OR "vitamin k dependent protein s" OR "cofactor protein s" OR "protein s" OR "protein c" OR "antiphospholipid" OR "glutamate" OR "beta 2 glycoprotein" OR "anti beta 2 glycoprotein" OR "adp" OR "adenosine diphosphate" OR "adenosine pyrophosphate" OR "adenosine 5' pyrophosphate" OR "serotonin" OR "5 hydroxytryptamine" OR "hippophaine" OR "3 2 aminoethyl 2 methyl 1 h indol 5 ol" OR "enteramine" OR "5 ht" OR "hydroxytryptamine" OR "thromboxane a2" OR "txa2" OR "arachidonic acid" OR "aa" OR "acetylsalicylic acid" OR "aspirin" OR "platelet derived growth factor" OR "pdgf receptor" OR "pdgf" OR "receptor tgf beta" OR "tgfbeta" OR "tgf beta" OR "platelet transforming growth factor" OR "gmp 140" OR "p selectin" OR "padgem" OR "cd62p antigen" OR "cd62p antigens" OR "cd62p" OR "alpha granule membrane protein" OR "lecam3" OR "gpvi" OR "platelet membrane glycoprotein p62" OR "glycoprotein gp vi" OR "platelet glycoprotein vi" OR "human glycoprotein vi" OR "human alpha2beta1" OR "integrin vla 2" OR "vla 2" OR "glycoprotein ia iia" OR "alpha2beta1integrin" OR "very late antigen 2" OR "late antigen 2" OR "cd49b cd29" OR "platelet membrane glycoprotein iaiia" OR "platelet membrane glycoprotein ia iia" OR "platelet membrane glycoprotein iaiia complex" OR "platelet membrane glycoprotein ia iia complex" OR "platelet glycoprotein gpiib iiia complex" OR "glycoproteins iib iiia" OR "glycoproteins iib iiia complex" OR "integrin alpha iib" OR "integrin alpha iib beta 3" OR "platelet glycoprotein gpib ix complex" OR "platelet glycoprotein gpib ix complex" OR "glycoprotein ib ix complex" OR "platelet membrane glycoprotein ib" OR "glycoprotein ib beta" OR "cd42c" OR "calcitonin gene related peptide" OR "calcitonin gene related peptide ii" OR "beta cgrp" OR "beta calcitonin gene related peptide" OR "alpha cgrp" OR "alpha calcitonin gene related peptide" OR "calcitonin gene related peptide i" OR "cgrp" OR "cyclo oxygenase i" OR "cox1" OR "prostaglandin h synthase 1" OR "prostaglandin synthase cyclooxygenase" OR "prostaglandin synthase" OR "cox 1 synthesis" OR "cox 1 prostaglandin" OR "cyclooxygenase 1" OR "endothelium derived vasoconstrictor factors" OR "vasoconstrictor factors" OR "endothelium derived endothelin 1" OR "nitric nitrogen" OR "endothelium derived nitric oxide" OR "vasodilator nitrates" OR "vasodilator nitric oxide" OR "von willebrand factor cleaving protease" OR "adamts13" OR "vwf cleaving protease" OR "vwf cleaving protease adamts13" OR "p2y12 receptors" OR "p2y12 receptor" OR "p2y12" OR "purinergic p2y12 receptors" OR "purinergic p2y12 receptor" OR "purinergic receptor p2y" OR "p2y adp receptor" OR "p2y adp receptors" OR "p selectin glycoprotein ligand 1" OR "p selectin" OR CD162 OR "platelet p selectin" OR "psgl 1 protein" OR "selectin P ligand protein" OR "endocannabinoid" OR "endocannabinoids" OR "cb2 receptor" OR "cannabinoid receptor cb2" OR "cannabinoid receptor 2" OR "cannabinoid receptor" OR "cannabinoid cb1 receptor" OR "cannabinoid cb2 receptor" OR "cannabinoid receptor cb1" OR "cannabinoid receptor 1" OR "cb1 cannabinoid receptor" OR "cb2 cannabinoid receptor" OR "glyceryl 2 arachidonate" OR "2 ag" OR "aea" OR "2 arachidonoylglycerol" OR "anandamide" OR "n arachidonoylethanolamine" OR "metalloproteinase 9" OR "matrix metalloproteinase 9" OR "mmp9" OR "metalloproteinase" OR "metallopeptidases" OR "metalloproteinases" OR "metallopeptidase" OR "type iv collagenase" OR "type iv collagenase mmp 2" OR "type iv collagenase mmp 9" OR "metalloproteinase mmp 9" OR "metalloproteinase 1" OR "mmp1" OR "metalloproteinase mmp1" OR "mmp 1" OR "mmp 9" OR "matrix metalloproteinase 1" OR "metalloproteinase 3" OR "mmp3" OR "matrix metalloproteinase 3" OR "metalloproteinase 2" OR "mmp2" OR "matrix metalloproteinase 2" OR "type iv collagenase mmp 2" OR "mmp 2 gelatinase a") AND ts=("female" OR "females" OR "women" OR "woman" OR "girl" OR "girls"))) NOT ti=("Tissue Plasminogen Activator*" OR "Aspirin*" OR therap* OR treat*) NOT ti=(("Case Report" OR "case report" OR "Review" OR "review" OR "systematic review" OR "systematic review" OR "meta analysis" OR "meta-analysis") NOT ("Clinical Study" OR "Clinical Trial" OR "trial" OR "RCT")) AND la=(english OR dutch) NOT TI=(("Child*" OR "Infant*" OR pediat* OR paediat*) NOT ("Adult*")) NOT dt=(meeting abstract)

**Cochrane**

<https://www.cochranelibrary.com/advanced-search/search-manager>

((("cerebrovascular accident" OR "stroke" OR "cva" OR "cerebrovascular accident" OR "cerebrovascular accidents" OR "cerebrovascular apoplexy" OR "apoplexy" OR "brain vascular accidents" OR "vascular accidents" OR "cerebrovascular stroke" OR "cerebrovascular strokes" OR "cerebrovascular apoplexy" OR "cerebral stroke" OR "cerebral strokes" OR "acute stroke" OR "acute strokes" OR "acute cerebrovascular accident" OR "acute cerebrovascular accidents" OR "cerebrovascular accidents" OR "brain infarction" OR "brain infarctions" OR "brain infarct" OR "brain infarcts" OR "anterior circulation infarction" OR "venous infarction" OR "venous infarctions" OR "posterior circulation infarction" OR "posterior circulation infarcts" OR "brain ischemia" OR "ischemic encephalopathy" OR "ischemic encephalopathies" OR "cerebral ischemia" OR "cerebral ischemias"):ti,ab,kw AND ("Migraine" OR "migraine" OR "migraine disorder" OR "migraine disorders" OR "migraines" OR "migraine headache" OR "migraine headaches" OR "headache" OR "headaches" OR "acute confusional migraine" OR "migraine hemicrania" OR "migraine variants" OR "migraine variant" OR "common migraine" OR "common migraines" OR "familial hemiplegic migraine" OR "familial hemiplegic migraines" OR "hemiplegic migraine" OR "classical migraine" OR "classical migraine attacks" OR "migraine with aura" OR "migraine with auras" OR "prolonged aura migraine" OR "migraineur" OR "migraineurs" OR "migraineurs without aura" OR "migrainous attack" OR "migrainous attacks" OR "migrainous aura" OR "migrainous auras" OR migrain*):ti,ab,kw AND ("blood coagulation" OR "blood coagulation factor" OR "blood coagulation factors" OR "coagulation factor" OR "coagulation factors" OR "clotting factor" OR "clotting factors" OR "blood clotting" OR "blood clotting factor" OR "blood clotting factors" OR "thromboses" OR "thrombus" OR "blood clot" OR "blood clots" OR "thrombus formation" OR "hemostases" OR "platelet activation" OR "platelet activations" OR "platelet aggregation" OR "platelet clotting" OR "platelet adhesion" OR "Blood Clotting Factor" OR "factor i" OR "blood coagulation factor i" OR "coagulation factor i" OR "fibrinogen" OR "gamma fibrinogen" OR "gamma fibrinogen" OR "fi" OR "factor ii" OR "blood coagulation factor ii" OR "differentiation reversal factor" OR "coagulation factor ii" OR "prothrombin" OR "fii" OR "thromboplastin" OR "coagulin" OR "factor iii" OR "fiii" OR "coagulation factor iii" OR "coagulation tissue factor" OR "tissue thromboplastin" OR "thromboplastin" OR "procoagulant" OR "tissue factor procoagulant" OR "coagulation factor iv" OR "factor iv" OR "calcium 40" OR "calcium" OR "fv" OR "factor five" OR "blood coagulation factor v" OR "coagulation factor v" OR "coagulation factor v" OR "factor v" OR "activated factor v" OR "coagulation factor va" OR "factor va" OR "factor v leiden" OR "factor five leiden" OR "factor seven" OR "coagulation factor vii" OR "factor vii" OR "blood coagulation factor vii" OR "proconvertin" OR "coagulation factor 7" OR "factor 7" OR "coagulation factor viia" OR "factor viia" OR "activated factor vii" OR "activated factor vii" OR "factor 7a" OR "factor 8" OR "coagulation factor viii" OR "thromboplastinogen" OR "blood coagulation factor viii" OR "factor eight" OR "hyatt c" OR "factor viiic" OR "f viii c" OR "factor viii" OR "blood coagulation factor viii" OR "coagulation factor viiia" OR "factor viiia" OR "coagulation factor viii" OR "thrombin activated factor viii" OR "factor 8a" OR "activated factor viii" OR "factor nine" OR "autoprothrombin ii" OR "christmas factor" OR "factor ix" OR "blood coagulation factor ix" OR "factor ix complex" OR "factor 9" OR "coagulation factor ix" OR "coagulation factor ixa" OR "factor ixa" OR "activated factor ix" OR "factor 9a" OR "plasma thromboplastin component" OR "factor ten" OR "autoprothrombin iii" OR "coagulation factor x" OR "factor x" OR "blood coagulation factor x" OR "stuart factor" OR "stuart prower factor" OR "factor 10" OR "activated factor x" OR "activated coagulation factor x" OR "factor xa" OR "blood coagulation factor x" OR "factor 10a" OR "autoprothrombin c" OR "factor eleven" OR "blood coagulation factor xi" OR "plasma thromboplastin" OR "plasma thromboplastin antecedent" OR "antecedent" OR "plasma thromboplastin" OR "thromboplastin antecedent" OR "coagulation factor xi" OR "factor xi" OR "coagulation factor 11" OR "factor 11" OR "activated factor xi" OR "coagulation factor xia" OR "factor xia" OR "blood coagulation factor xi" OR "factor 11a" OR "factor twelve" OR "hageman factor" OR "factor 12" OR "coagulation factor xii" OR "activated factor xii" OR "coagulation factor xiia" OR "factor xiia" OR "blood coagulation factor xii" OR "activated prekallikrein" OR "prekallikrein" OR "factor xii" OR "activated hageman factor" OR "hageman factor fragment" OR "factor thirteen" OR "coagulation factor xiii" OR "factor xiii" OR "factor xiii" OR "transamidase" OR "fibrinase" OR "laki lorand factor" OR "blood coagulation factor xiii" OR "factor 13" OR "fibrin stabilizing factor" OR "stabilizing factor" OR "activated factor xiii" OR "factor 13a" OR "plasma transglutaminase" OR "transglutaminase" OR "beta thromboglobulin" OR "beta 2 thromboglobulin" OR "thromboglobulin" OR "fibrinopeptides a" OR "fibrinopeptides b" OR "kalliginogenase" OR "kallikrein" OR "kallikrein kininogenase" OR "callicrein" OR "kinin forming enzyme" OR "kinin forming" OR "kallikrein light chain" OR "kallikrein padutin" OR "kallikrein a" OR "beta kallikrein" OR "kallikrein b" OR "alpha kallikrein" OR "plasma kallikrein" OR "kininogenin" OR "kallikrein i" OR "plasma prokallikrein" OR "prokallikrein" OR "prokinins" OR "cystatins" OR "t kininogen" OR "low molecular weight kininogens" OR "lmwk" OR "high molecular weight kininogens" OR "hmwk" OR "fitzgerald factor" OR "plasminogen" OR "plasminogen activator" OR "plasminogen activator inhibitors" OR "endothelial plasminogen activator" OR "endothelial plasminogen activator inhibitor" OR "pai 1" OR "serpin e1" OR "serpine1" OR "serpine1 protein" OR "type 1 plasminogen activator inhibitor" OR "serpin b2" OR "type 2 plasminogen activator inhibitor" OR "pai 2" OR "pai 3" OR "plasminogen activator inhibitor" OR "activated protein c inhibitor" OR "plasminogen activator inhibitor 3" OR "phosphorylcholine" OR "acetyl glyceryl phosphorylcholine" OR "aggregating factor" OR "platelet aggregation enhancing factor" OR "agepc" OR "thrombocyte aggregating activity" OR "alkyl 2 acetyl sn glycerophosphocholine" OR "1 alkyl 2 acetyl sn glyceryl 3 phosphorylcholine" OR "1 alkyl 2 acetylglycerophosphocholine" OR "paf acether" OR "platelet activating substance" OR "platelet activating substances" OR "pf 3" OR "platelet factor 3" OR "chemokine cxcl4" OR "cxcl4" OR "chemokine pf4" OR "platelet factor 4" OR "heparin neutralizing protein" OR "antiheparin factor" OR "thrombase" OR "thrombostat" OR "alpha thrombin" OR "thrombin jmi" OR "jmi" OR "thrombin" OR "beta thrombin" OR "gamma thrombin" OR "plasminogen activator" OR "tissue activator d 44" OR "tisokinase" OR "tissue type plasminogen activator" OR "ttpa" OR "t plasminogen activator" OR "tissue type activator" OR "rt pa" OR "factor viiir ag" OR "factor viiir rco" OR "ristocetin willebrand factor" OR "von willebrand protein" OR "von willebrand factor" OR "factor viii related antigen" OR "ristocetin cofactor" OR "plasma factor viii complex" OR "vitamin k dependent protein s" OR "cofactor protein s" OR "protein s" OR "protein c" OR "antiphospholipid" OR "glutamate" OR "beta 2 glycoprotein" OR "anti beta 2 glycoprotein" OR "adp" OR "adenosine diphosphate" OR "adenosine pyrophosphate" OR "adenosine 5' pyrophosphate" OR "serotonin" OR "5 hydroxytryptamine" OR "hippophaine" OR "3 2 aminoethyl 2 methyl 1 h indol 5 ol" OR "enteramine" OR "5 ht" OR "hydroxytryptamine" OR "thromboxane a2" OR "txa2" OR "arachidonic acid" OR "aa" OR "acetylsalicylic acid" OR "aspirin" OR "platelet derived growth factor" OR "pdgf receptor" OR "pdgf" OR "receptor tgf beta" OR "tgfbeta" OR "tgf beta" OR "platelet transforming growth factor" OR "gmp 140" OR "p selectin" OR "padgem" OR "cd62p antigen" OR "cd62p antigens" OR "cd62p" OR "alpha granule membrane protein" OR "lecam3" OR "gpvi" OR "platelet membrane glycoprotein p62" OR "glycoprotein gp vi" OR "platelet glycoprotein vi" OR "human glycoprotein vi" OR "human alpha2beta1" OR "integrin vla 2" OR "vla 2" OR "glycoprotein ia iia" OR "alpha2beta1integrin" OR "very late antigen 2" OR "late antigen 2" OR "cd49b cd29" OR "platelet membrane glycoprotein iaiia" OR "platelet membrane glycoprotein ia iia" OR "platelet membrane glycoprotein iaiia complex" OR "platelet membrane glycoprotein ia iia complex" OR "platelet glycoprotein gpiib iiia complex" OR "glycoproteins iib iiia" OR "glycoproteins iib iiia complex" OR "integrin alpha iib" OR "integrin alpha iib beta 3" OR "platelet glycoprotein gpib ix complex" OR "platelet glycoprotein gpib ix complex" OR "glycoprotein ib ix complex" OR "platelet membrane glycoprotein ib" OR "glycoprotein ib beta" OR "cd42c" OR "calcitonin gene related peptide" OR "calcitonin gene related peptide ii" OR "beta cgrp" OR "beta calcitonin gene related peptide" OR "alpha cgrp" OR "alpha calcitonin gene related peptide" OR "calcitonin gene related peptide i" OR "cgrp" OR "cyclo oxygenase i" OR "cox1" OR "prostaglandin h synthase 1" OR "prostaglandin synthase cyclooxygenase" OR "prostaglandin synthase" OR "cox 1 synthesis" OR "cox 1 prostaglandin" OR "cyclooxygenase 1" OR "endothelium derived vasoconstrictor factors" OR "vasoconstrictor factors" OR "endothelium derived endothelin 1" OR "nitric nitrogen" OR "endothelium derived nitric oxide" OR "vasodilator nitrates" OR "vasodilator nitric oxide" OR "von willebrand factor cleaving protease" OR "adamts13" OR "vwf cleaving protease" OR "vwf cleaving protease adamts13" OR "p2y12 receptors" OR "p2y12 receptor" OR "p2y12" OR "purinergic p2y12 receptors" OR "purinergic p2y12 receptor" OR "purinergic receptor p2y" OR "p2y adp receptor" OR "p2y adp receptors" OR "p selectin glycoprotein ligand 1" OR "p selectin" OR CD162 OR "platelet p selectin" OR "psgl 1 protein" OR "selectin P ligand protein" OR "endocannabinoid" OR "endocannabinoids" OR "cb2 receptor" OR "cannabinoid receptor cb2" OR "cannabinoid receptor 2" OR "cannabinoid receptor" OR "cannabinoid cb1 receptor" OR "cannabinoid cb2 receptor" OR "cannabinoid receptor cb1" OR "cannabinoid receptor 1" OR "cb1 cannabinoid receptor" OR "cb2 cannabinoid receptor" OR "glyceryl 2 arachidonate" OR "2 ag" OR "aea" OR "2 arachidonoylglycerol" OR "anandamide" OR "n arachidonoylethanolamine" OR "metalloproteinase 9" OR "matrix metalloproteinase 9" OR "mmp9" OR "metalloproteinase" OR "metallopeptidases" OR "metalloproteinases" OR "metallopeptidase" OR "type iv collagenase" OR "type iv collagenase mmp 2" OR "type iv collagenase mmp 9" OR "metalloproteinase mmp 9" OR "metalloproteinase 1" OR "mmp1" OR "metalloproteinase mmp1" OR "mmp 1" OR "mmp 9" OR "matrix metalloproteinase 1" OR "metalloproteinase 3" OR "mmp3" OR "matrix metalloproteinase 3" OR "metalloproteinase 2" OR "mmp2" OR "matrix metalloproteinase 2" OR "type iv collagenase mmp 2" OR "mmp 2 gelatinase a"):ti,ab,kw AND ("female" OR "females" OR "women" OR "woman" OR "girl" OR "girls"):ti,ab,kw) OR (("cerebrovascular accident" OR "stroke" OR "cva" OR "cerebrovascular accident" OR "cerebrovascular accidents" OR "cerebrovascular apoplexy" OR "apoplexy" OR "brain vascular accidents" OR "vascular accidents" OR "cerebrovascular stroke" OR "cerebrovascular strokes" OR "cerebrovascular apoplexy" OR "cerebral stroke" OR "cerebral strokes" OR "acute stroke" OR "acute strokes" OR "acute cerebrovascular accident" OR "acute cerebrovascular accidents" OR "cerebrovascular accidents" OR "brain infarction" OR "brain infarctions" OR "brain infarct" OR "brain infarcts" OR "anterior circulation infarction" OR "venous infarction" OR "venous infarctions" OR "posterior circulation infarction" OR "posterior circulation infarcts" OR "brain ischemia" OR "ischemic encephalopathy" OR "ischemic encephalopathies" OR "cerebral ischemia" OR "cerebral ischemias"):ti AND ("blood coagulation" OR "blood coagulation factor" OR "blood coagulation factors" OR "coagulation factor" OR "coagulation factors" OR "clotting factor" OR "clotting factors" OR "blood clotting" OR "blood clotting factor" OR "blood clotting factors" OR "thromboses" OR "thrombus" OR "blood clot" OR "blood clots" OR "thrombus formation" OR "hemostases" OR "platelet activation" OR "platelet activations" OR "platelet aggregation" OR "platelet clotting" OR "platelet adhesion" OR "Blood Clotting Factor" OR "factor i" OR "blood coagulation factor i" OR "coagulation factor i" OR "fibrinogen" OR "gamma fibrinogen" OR "gamma fibrinogen" OR "fi" OR "factor ii" OR "blood coagulation factor ii" OR "differentiation reversal factor" OR "coagulation factor ii" OR "prothrombin" OR "fii" OR "thromboplastin" OR "coagulin" OR "factor iii" OR "fiii" OR "coagulation factor iii" OR "coagulation tissue factor" OR "tissue thromboplastin" OR "thromboplastin" OR "procoagulant" OR "tissue factor procoagulant" OR "coagulation factor iv" OR "factor iv" OR "calcium 40" OR "calcium" OR "fv" OR "factor five" OR "blood coagulation factor v" OR "coagulation factor v" OR "coagulation factor v" OR "factor v" OR "activated factor v" OR "coagulation factor va" OR "factor va" OR "factor v leiden" OR "factor five leiden" OR "factor seven" OR "coagulation factor vii" OR "factor vii" OR "blood coagulation factor vii" OR "proconvertin" OR "coagulation factor 7" OR "factor 7" OR "coagulation factor viia" OR "factor viia" OR "activated factor vii" OR "activated factor vii" OR "factor 7a" OR "factor 8" OR "coagulation factor viii" OR "thromboplastinogen" OR "blood coagulation factor viii" OR "factor eight" OR "hyatt c" OR "factor viiic" OR "f viii c" OR "factor viii" OR "blood coagulation factor viii" OR "coagulation factor viiia" OR "factor viiia" OR "coagulation factor viii" OR "thrombin activated factor viii" OR "factor 8a" OR "activated factor viii" OR "factor nine" OR "autoprothrombin ii" OR "christmas factor" OR "factor ix" OR "blood coagulation factor ix" OR "factor ix complex" OR "factor 9" OR "coagulation factor ix" OR "coagulation factor ixa" OR "factor ixa" OR "activated factor ix" OR "factor 9a" OR "plasma thromboplastin component" OR "factor ten" OR "autoprothrombin iii" OR "coagulation factor x" OR "factor x" OR "blood coagulation factor x" OR "stuart factor" OR "stuart prower factor" OR "factor 10" OR "activated factor x" OR "activated coagulation factor x" OR "factor xa" OR "blood coagulation factor x" OR "factor 10a" OR "autoprothrombin c" OR "factor eleven" OR "blood coagulation factor xi" OR "plasma thromboplastin" OR "plasma thromboplastin antecedent" OR "antecedent" OR "plasma thromboplastin" OR "thromboplastin antecedent" OR "coagulation factor xi" OR "factor xi" OR "coagulation factor 11" OR "factor 11" OR "activated factor xi" OR "coagulation factor xia" OR "factor xia" OR "blood coagulation factor xi" OR "factor 11a" OR "factor twelve" OR "hageman factor" OR "factor 12" OR "coagulation factor xii" OR "activated factor xii" OR "coagulation factor xiia" OR "factor xiia" OR "blood coagulation factor xii" OR "activated prekallikrein" OR "prekallikrein" OR "factor xii" OR "activated hageman factor" OR "hageman factor fragment" OR "factor thirteen" OR "coagulation factor xiii" OR "factor xiii" OR "factor xiii" OR "transamidase" OR "fibrinase" OR "laki lorand factor" OR "blood coagulation factor xiii" OR "factor 13" OR "fibrin stabilizing factor" OR "stabilizing factor" OR "activated factor xiii" OR "factor 13a" OR "plasma transglutaminase" OR "transglutaminase" OR "beta thromboglobulin" OR "beta 2 thromboglobulin" OR "thromboglobulin" OR "fibrinopeptides a" OR "fibrinopeptides b" OR "kalliginogenase" OR "kallikrein" OR "kallikrein kininogenase" OR "callicrein" OR "kinin forming enzyme" OR "kinin forming" OR "kallikrein light chain" OR "kallikrein padutin" OR "kallikrein a" OR "beta kallikrein" OR "kallikrein b" OR "alpha kallikrein" OR "plasma kallikrein" OR "kininogenin" OR "kallikrein i" OR "plasma prokallikrein" OR "prokallikrein" OR "prokinins" OR "cystatins" OR "t kininogen" OR "low molecular weight kininogens" OR "lmwk" OR "high molecular weight kininogens" OR "hmwk" OR "fitzgerald factor" OR "plasminogen" OR "plasminogen activator" OR "plasminogen activator inhibitors" OR "endothelial plasminogen activator" OR "endothelial plasminogen activator inhibitor" OR "pai 1" OR "serpin e1" OR "serpine1" OR "serpine1 protein" OR "type 1 plasminogen activator inhibitor" OR "serpin b2" OR "type 2 plasminogen activator inhibitor" OR "pai 2" OR "pai 3" OR "plasminogen activator inhibitor" OR "activated protein c inhibitor" OR "plasminogen activator inhibitor 3" OR "phosphorylcholine" OR "acetyl glyceryl phosphorylcholine" OR "aggregating factor" OR "platelet aggregation enhancing factor" OR "agepc" OR "thrombocyte aggregating activity" OR "alkyl 2 acetyl sn glycerophosphocholine" OR "1 alkyl 2 acetyl sn glyceryl 3 phosphorylcholine" OR "1 alkyl 2 acetylglycerophosphocholine" OR "paf acether" OR "platelet activating substance" OR "platelet activating substances" OR "pf 3" OR "platelet factor 3" OR "chemokine cxcl4" OR "cxcl4" OR "chemokine pf4" OR "platelet factor 4" OR "heparin neutralizing protein" OR "antiheparin factor" OR "thrombase" OR "thrombostat" OR "alpha thrombin" OR "thrombin jmi" OR "jmi" OR "thrombin" OR "beta thrombin" OR "gamma thrombin" OR "plasminogen activator" OR "tissue activator d 44" OR "tisokinase" OR "tissue type plasminogen activator" OR "ttpa" OR "t plasminogen activator" OR "tissue type activator" OR "rt pa" OR "factor viiir ag" OR "factor viiir rco" OR "ristocetin willebrand factor" OR "von willebrand protein" OR "von willebrand factor" OR "factor viii related antigen" OR "ristocetin cofactor" OR "plasma factor viii complex" OR "vitamin k dependent protein s" OR "cofactor protein s" OR "protein s" OR "protein c" OR "antiphospholipid" OR "glutamate" OR "beta 2 glycoprotein" OR "anti beta 2 glycoprotein" OR "adp" OR "adenosine diphosphate" OR "adenosine pyrophosphate" OR "adenosine 5' pyrophosphate" OR "serotonin" OR "5 hydroxytryptamine" OR "hippophaine" OR "3 2 aminoethyl 2 methyl 1 h indol 5 ol" OR "enteramine" OR "5 ht" OR "hydroxytryptamine" OR "thromboxane a2" OR "txa2" OR "arachidonic acid" OR "aa" OR "acetylsalicylic acid" OR "aspirin" OR "platelet derived growth factor" OR "pdgf receptor" OR "pdgf" OR "receptor tgf beta" OR "tgfbeta" OR "tgf beta" OR "platelet transforming growth factor" OR "gmp 140" OR "p selectin" OR "padgem" OR "cd62p antigen" OR "cd62p antigens" OR "cd62p" OR "alpha granule membrane protein" OR "lecam3" OR "gpvi" OR "platelet membrane glycoprotein p62" OR "glycoprotein gp vi" OR "platelet glycoprotein vi" OR "human glycoprotein vi" OR "human alpha2beta1" OR "integrin vla 2" OR "vla 2" OR "glycoprotein ia iia" OR "alpha2beta1integrin" OR "very late antigen 2" OR "late antigen 2" OR "cd49b cd29" OR "platelet membrane glycoprotein iaiia" OR "platelet membrane glycoprotein ia iia" OR "platelet membrane glycoprotein iaiia complex" OR "platelet membrane glycoprotein ia iia complex" OR "platelet glycoprotein gpiib iiia complex" OR "glycoproteins iib iiia" OR "glycoproteins iib iiia complex" OR "integrin alpha iib" OR "integrin alpha iib beta 3" OR "platelet glycoprotein gpib ix complex" OR "platelet glycoprotein gpib ix complex" OR "glycoprotein ib ix complex" OR "platelet membrane glycoprotein ib" OR "glycoprotein ib beta" OR "cd42c" OR "calcitonin gene related peptide" OR "calcitonin gene related peptide ii" OR "beta cgrp" OR "beta calcitonin gene related peptide" OR "alpha cgrp" OR "alpha calcitonin gene related peptide" OR "calcitonin gene related peptide i" OR "cgrp" OR "cyclo oxygenase i" OR "cox1" OR "prostaglandin h synthase 1" OR "prostaglandin synthase cyclooxygenase" OR "prostaglandin synthase" OR "cox 1 synthesis" OR "cox 1 prostaglandin" OR "cyclooxygenase 1" OR "endothelium derived vasoconstrictor factors" OR "vasoconstrictor factors" OR "endothelium derived endothelin 1" OR "nitric nitrogen" OR "endothelium derived nitric oxide" OR "vasodilator nitrates" OR "vasodilator nitric oxide" OR "von willebrand factor cleaving protease" OR "adamts13" OR "vwf cleaving protease" OR "vwf cleaving protease adamts13" OR "p2y12 receptors" OR "p2y12 receptor" OR "p2y12" OR "purinergic p2y12 receptors" OR "purinergic p2y12 receptor" OR "purinergic receptor p2y" OR "p2y adp receptor" OR "p2y adp receptors" OR "p selectin glycoprotein ligand 1" OR "p selectin" OR CD162 OR "platelet p selectin" OR "psgl 1 protein" OR "selectin P ligand protein" OR "endocannabinoid" OR "endocannabinoids" OR "cb2 receptor" OR "cannabinoid receptor cb2" OR "cannabinoid receptor 2" OR "cannabinoid receptor" OR "cannabinoid cb1 receptor" OR "cannabinoid cb2 receptor" OR "cannabinoid receptor cb1" OR "cannabinoid receptor 1" OR "cb1 cannabinoid receptor" OR "cb2 cannabinoid receptor" OR "glyceryl 2 arachidonate" OR "2 ag" OR "aea" OR "2 arachidonoylglycerol" OR "anandamide" OR "n arachidonoylethanolamine" OR "metalloproteinase 9" OR "matrix metalloproteinase 9" OR "mmp9" OR "metalloproteinase" OR "metallopeptidases" OR "metalloproteinases" OR "metallopeptidase" OR "type iv collagenase" OR "type iv collagenase mmp 2" OR "type iv collagenase mmp 9" OR "metalloproteinase mmp 9" OR "metalloproteinase 1" OR "mmp1" OR "metalloproteinase mmp1" OR "mmp 1" OR "mmp 9" OR "matrix metalloproteinase 1" OR "metalloproteinase 3" OR "mmp3" OR "matrix metalloproteinase 3" OR "metalloproteinase 2" OR "mmp2" OR "matrix metalloproteinase 2" OR "type iv collagenase mmp 2" OR "mmp 2 gelatinase a"):ti AND ("female" OR "females" OR "women" OR "woman" OR "girl" OR "girls"):ti,ab,kw)) NOT ("Tissue Plasminogen Activator*" OR "Aspirin*" OR therap* OR treat*):ti NOT (("Child*" OR "Infant*" OR pediat* OR paediat*) NOT "Adult*"):ti NOT (conference abstract):pt

**Emcare** <http://ovidsp.ovid.com/ovidweb.cgi?T=JS&NEWS=n&CSC=Y&PAGE=main&D=emcr>

(((exp "cerebrovascular accident"/ OR "stroke".ti,ab OR "cva".ti,ab OR "cerebrovascular accident".ti,ab OR "cerebrovascular accidents".ti,ab OR "cerebrovascular apoplexy".ti,ab OR "apoplexy".ti,ab OR "brain vascular accidents".ti,ab OR "vascular accidents".ti,ab OR "cerebrovascular stroke".ti,ab OR "cerebrovascular strokes".ti,ab OR "cerebrovascular apoplexy".ti,ab OR "cerebral stroke".ti,ab OR "cerebral strokes".ti,ab OR "acute stroke".ti,ab OR "acute strokes".ti,ab OR "acute cerebrovascular accident".ti,ab OR "acute cerebrovascular accidents".ti,ab OR "cerebrovascular accidents".ti,ab OR "brain infarction".ti,ab OR "brain infarctions".ti,ab OR "brain infarct".ti,ab OR "brain infarcts".ti,ab OR "anterior circulation infarction".ti,ab OR "venous infarction".ti,ab OR "venous infarctions".ti,ab OR "posterior circulation infarction".ti,ab OR "posterior circulation infarcts".ti,ab OR "brain ischemia".ti,ab OR "ischemic encephalopathy".ti,ab OR "ischemic encephalopathies".ti,ab OR "cerebral ischemia".ti,ab OR "cerebral ischemias".ti,ab) AND (exp "Migraine"/ OR "migraine".ti,ab OR "migraine disorder".ti,ab OR "migraine disorders".ti,ab OR "migraines".ti,ab OR "migraine headache".ti,ab OR "migraine headaches".ti,ab OR "headache".ti,ab OR "headaches".ti,ab OR "acute confusional migraine".ti,ab OR "migraine hemicrania".ti,ab OR "migraine variants".ti,ab OR "migraine variant".ti,ab OR "common migraine".ti,ab OR "common migraines".ti,ab OR "familial hemiplegic migraine".ti,ab OR "familial hemiplegic migraines".ti,ab OR "hemiplegic migraine".ti,ab OR "classical migraine".ti,ab OR "classical migraine attacks".ti,ab OR "migraine with aura".ti,ab OR "migraine with auras".ti,ab OR "prolonged aura migraine".ti,ab OR "migraineur".ti,ab OR "migraineurs".ti,ab OR "migraineurs without aura".ti,ab OR "migrainous attack".ti,ab OR "migrainous attacks".ti,ab OR "migrainous aura".ti,ab OR "migrainous auras".ti,ab) AND ("blood coagulation".ti,ab OR "blood coagulation factor".ti,ab OR "blood coagulation factors".ti,ab OR "coagulation factor".ti,ab OR "coagulation factors".ti,ab OR "clotting factor".ti,ab OR "clotting factors".ti,ab OR "blood clotting".ti,ab OR "blood clotting factor".ti,ab OR "blood clotting factors".ti,ab OR "thromboses".ti,ab OR "thrombus".ti,ab OR "blood clot".ti,ab OR "blood clots".ti,ab OR "thrombus formation".ti,ab OR "hemostases".ti,ab OR "platelet activation".ti,ab OR "platelet activations".ti,ab OR "platelet aggregation".ti,ab OR "platelet clotting".ti,ab OR "platelet adhesion".ti,ab OR exp *"Blood Clotting Factor"/ OR "factor i".ti,ab OR "blood coagulation factor i".ti,ab OR "coagulation factor i".ti,ab OR "fibrinogen".ti,ab OR "gamma fibrinogen".ti,ab OR "gamma fibrinogen".ti,ab OR "fi".ti,ab OR "factor ii".ti,ab OR "blood coagulation factor ii".ti,ab OR "differentiation reversal factor".ti,ab OR "coagulation factor ii".ti,ab OR "prothrombin".ti,ab OR "fii".ti,ab OR "thromboplastin".ti,ab OR "coagulin".ti,ab OR "factor iii".ti,ab OR "fiii".ti,ab OR "coagulation factor iii".ti,ab OR "coagulation tissue factor".ti,ab OR "tissue thromboplastin".ti,ab OR "thromboplastin".ti,ab OR "procoagulant".ti,ab OR "tissue factor procoagulant".ti,ab OR "coagulation factor iv".ti,ab OR "factor iv".ti,ab OR "calcium 40".ti,ab OR "calcium".ti,ab OR "fv".ti,ab OR "factor five".ti,ab OR "blood coagulation factor v".ti,ab OR "coagulation factor v".ti,ab OR "coagulation factor v".ti,ab OR "factor v".ti,ab OR "activated factor v".ti,ab OR "coagulation factor va".ti,ab OR "factor va".ti,ab OR "factor v leiden".ti,ab OR "factor five leiden".ti,ab OR "factor seven".ti,ab OR "coagulation factor vii".ti,ab OR "factor vii".ti,ab OR "blood coagulation factor vii".ti,ab OR "proconvertin".ti,ab OR "coagulation factor 7".ti,ab OR "factor 7".ti,ab OR "coagulation factor viia".ti,ab OR "factor viia".ti,ab OR "activated factor vii".ti,ab OR "activated factor vii".ti,ab OR "factor 7a".ti,ab OR "factor 8".ti,ab OR "coagulation factor viii".ti,ab OR "thromboplastinogen".ti,ab OR "blood coagulation factor viii".ti,ab OR "factor eight".ti,ab OR "hyatt c".ti,ab OR "factor viiic".ti,ab OR "f viii c".ti,ab OR "factor viii".ti,ab OR "blood coagulation factor viii".ti,ab OR "coagulation factor viiia".ti,ab OR "factor viiia".ti,ab OR "coagulation factor viii".ti,ab OR "thrombin activated factor viii".ti,ab OR "factor 8a".ti,ab OR "activated factor viii".ti,ab OR "factor nine".ti,ab OR "autoprothrombin ii".ti,ab OR "christmas factor".ti,ab OR "factor ix".ti,ab OR "blood coagulation factor ix".ti,ab OR "factor ix complex".ti,ab OR "factor 9".ti,ab OR "coagulation factor ix".ti,ab OR "coagulation factor ixa".ti,ab OR "factor ixa".ti,ab OR "activated factor ix".ti,ab OR "factor 9a".ti,ab OR "plasma thromboplastin component".ti,ab OR "factor ten".ti,ab OR "autoprothrombin iii".ti,ab OR "coagulation factor x".ti,ab OR "factor x".ti,ab OR "blood coagulation factor x".ti,ab OR "stuart factor".ti,ab OR "stuart prower factor".ti,ab OR "factor 10".ti,ab OR "activated factor x".ti,ab OR "activated coagulation factor x".ti,ab OR "factor xa".ti,ab OR "blood coagulation factor x".ti,ab OR "factor 10a".ti,ab OR "autoprothrombin c".ti,ab OR "factor eleven".ti,ab OR "blood coagulation factor xi".ti,ab OR "plasma thromboplastin".ti,ab OR "plasma thromboplastin antecedent".ti,ab OR "antecedent".ti,ab OR "plasma thromboplastin".ti,ab OR "thromboplastin antecedent".ti,ab OR "coagulation factor xi".ti,ab OR "factor xi".ti,ab OR "coagulation factor 11".ti,ab OR "factor 11".ti,ab OR "activated factor xi".ti,ab OR "coagulation factor xia".ti,ab OR "factor xia".ti,ab OR "blood coagulation factor xi".ti,ab OR "factor 11a".ti,ab OR "factor twelve".ti,ab OR "hageman factor".ti,ab OR "factor 12".ti,ab OR "coagulation factor xii".ti,ab OR "activated factor xii".ti,ab OR "coagulation factor xiia".ti,ab OR "factor xiia".ti,ab OR "blood coagulation factor xii".ti,ab OR "activated prekallikrein".ti,ab OR "prekallikrein".ti,ab OR "factor xii".ti,ab OR "activated hageman factor".ti,ab OR "hageman factor fragment".ti,ab OR "factor thirteen".ti,ab OR "coagulation factor xiii".ti,ab OR "factor xiii".ti,ab OR "factor xiii".ti,ab OR "transamidase".ti,ab OR "fibrinase".ti,ab OR "laki lorand factor".ti,ab OR "blood coagulation factor xiii".ti,ab OR "factor 13".ti,ab OR "fibrin stabilizing factor".ti,ab OR "stabilizing factor".ti,ab OR "activated factor xiii".ti,ab OR "factor 13a".ti,ab OR "plasma transglutaminase".ti,ab OR "transglutaminase".ti,ab OR "beta thromboglobulin".ti,ab OR "beta 2 thromboglobulin".ti,ab OR "thromboglobulin".ti,ab OR "fibrinopeptides a".ti,ab OR "fibrinopeptides b".ti,ab OR "kalliginogenase".ti,ab OR "kallikrein".ti,ab OR "kallikrein kininogenase".ti,ab OR "callicrein".ti,ab OR "kinin forming enzyme".ti,ab OR "kinin forming".ti,ab OR "kallikrein light chain".ti,ab OR "kallikrein padutin".ti,ab OR "kallikrein a".ti,ab OR "beta kallikrein".ti,ab OR "kallikrein b".ti,ab OR "alpha kallikrein".ti,ab OR "plasma kallikrein".ti,ab OR "kininogenin".ti,ab OR "kallikrein i".ti,ab OR "plasma prokallikrein".ti,ab OR "prokallikrein".ti,ab OR "prokinins".ti,ab OR "cystatins".ti,ab OR "t kininogen".ti,ab OR "low molecular weight kininogens".ti,ab OR "lmwk".ti,ab OR "high molecular weight kininogens".ti,ab OR "hmwk".ti,ab OR "fitzgerald factor".ti,ab OR "plasminogen".ti,ab OR "plasminogen activator".ti,ab OR "plasminogen activator inhibitors".ti,ab OR "endothelial plasminogen activator".ti,ab OR "endothelial plasminogen activator inhibitor".ti,ab OR "pai 1".ti,ab OR "serpin e1".ti,ab OR "serpine1".ti,ab OR "serpine1 protein".ti,ab OR "type 1 plasminogen activator inhibitor".ti,ab OR "serpin b2".ti,ab OR "type 2 plasminogen activator inhibitor".ti,ab OR "pai 2".ti,ab OR "pai 3".ti,ab OR "plasminogen activator inhibitor".ti,ab OR "activated protein c inhibitor".ti,ab OR "plasminogen activator inhibitor 3".ti,ab OR "phosphorylcholine".ti,ab OR "acetyl glyceryl phosphorylcholine".ti,ab OR "aggregating factor".ti,ab OR "platelet aggregation enhancing factor".ti,ab OR "agepc".ti,ab OR "thrombocyte aggregating activity".ti,ab OR "alkyl 2 acetyl sn glycerophosphocholine".ti,ab OR "1 alkyl 2 acetyl sn glyceryl 3 phosphorylcholine".ti,ab OR "1 alkyl 2 acetylglycerophosphocholine".ti,ab OR "paf acether".ti,ab OR "platelet activating substance".ti,ab OR "platelet activating substances".ti,ab OR "pf 3".ti,ab OR "platelet factor 3".ti,ab OR "chemokine cxcl4".ti,ab OR "cxcl4".ti,ab OR "chemokine pf4".ti,ab OR "platelet factor 4".ti,ab OR "heparin neutralizing protein".ti,ab OR "antiheparin factor".ti,ab OR "thrombase".ti,ab OR "thrombostat".ti,ab OR "alpha thrombin".ti,ab OR "thrombin jmi".ti,ab OR "jmi".ti,ab OR "thrombin".ti,ab OR "beta thrombin".ti,ab OR "gamma thrombin".ti,ab OR "plasminogen activator".ti,ab OR "tissue activator d 44".ti,ab OR "tisokinase".ti,ab OR "tissue type plasminogen activator".ti,ab OR "ttpa".ti,ab OR "t plasminogen activator".ti,ab OR "tissue type activator".ti,ab OR "rt pa".ti,ab OR "factor viiir ag".ti,ab OR "factor viiir rco".ti,ab OR "ristocetin willebrand factor".ti,ab OR "von willebrand protein".ti,ab OR "von willebrand factor".ti,ab OR "factor viii related antigen".ti,ab OR "ristocetin cofactor".ti,ab OR "plasma factor viii complex".ti,ab OR "vitamin k dependent protein s".ti,ab OR "cofactor protein s".ti,ab OR "protein s".ti,ab OR "protein c".ti,ab OR "antiphospholipid".ti,ab OR "glutamate".ti,ab OR "beta 2 glycoprotein".ti,ab OR "anti beta 2 glycoprotein".ti,ab OR "adp".ti,ab OR "adenosine diphosphate".ti,ab OR "adenosine pyrophosphate".ti,ab OR "adenosine 5' pyrophosphate".ti,ab OR "serotonin".ti,ab OR "5 hydroxytryptamine".ti,ab OR "hippophaine".ti,ab OR "3 2 aminoethyl 2 methyl 1 h indol 5 ol".ti,ab OR "enteramine".ti,ab OR "5 ht".ti,ab OR "hydroxytryptamine".ti,ab OR "thromboxane a2".ti,ab OR "txa2".ti,ab OR "arachidonic acid".ti,ab OR "aa".ti,ab OR "acetylsalicylic acid".ti,ab OR "aspirin".ti,ab OR "platelet derived growth factor".ti,ab OR "pdgf receptor".ti,ab OR "pdgf".ti,ab OR "receptor tgf beta".ti,ab OR "tgfbeta".ti,ab OR "tgf beta".ti,ab OR "platelet transforming growth factor".ti,ab OR "gmp 140".ti,ab OR "p selectin".ti,ab OR "padgem".ti,ab OR "cd62p antigen".ti,ab OR "cd62p antigens".ti,ab OR "cd62p".ti,ab OR "alpha granule membrane protein".ti,ab OR "lecam3".ti,ab OR "gpvi".ti,ab OR "platelet membrane glycoprotein p62".ti,ab OR "glycoprotein gp vi".ti,ab OR "platelet glycoprotein vi".ti,ab OR "human glycoprotein vi".ti,ab OR "human alpha2beta1".ti,ab OR "integrin vla 2".ti,ab OR "vla 2".ti,ab OR "glycoprotein ia iia".ti,ab OR "alpha2beta1integrin".ti,ab OR "very late antigen 2".ti,ab OR "late antigen 2".ti,ab OR "cd49b cd29".ti,ab OR "platelet membrane glycoprotein ia/iia".ti,ab OR "platelet membrane glycoprotein ia iia".ti,ab OR "platelet membrane glycoprotein ia/iia complex".ti,ab OR "platelet membrane glycoprotein ia iia complex".ti,ab OR "platelet glycoprotein gpiib iiia complex".ti,ab OR "glycoproteins iib iiia".ti,ab OR "glycoproteins iib iiia complex".ti,ab OR "integrin alpha iib".ti,ab OR "integrin alpha iib beta 3".ti,ab OR "platelet glycoprotein gpib ix complex".ti,ab OR "platelet glycoprotein gpib ix complex".ti,ab OR "glycoprotein ib ix complex".ti,ab OR "platelet membrane glycoprotein ib".ti,ab OR "glycoprotein ib beta".ti,ab OR "cd42c".ti,ab OR "calcitonin gene related peptide".ti,ab OR "calcitonin gene related peptide ii".ti,ab OR "beta cgrp".ti,ab OR "beta calcitonin gene related peptide".ti,ab OR "alpha cgrp".ti,ab OR "alpha calcitonin gene related peptide".ti,ab OR "calcitonin gene related peptide i".ti,ab OR "cgrp".ti,ab OR "cyclo oxygenase i".ti,ab OR "cox1".ti,ab OR "prostaglandin h synthase 1".ti,ab OR "prostaglandin synthase cyclooxygenase".ti,ab OR "prostaglandin synthase".ti,ab OR "cox 1 synthesis".ti,ab OR "cox 1 prostaglandin".ti,ab OR "cyclooxygenase 1".ti,ab OR "endothelium derived vasoconstrictor factors".ti,ab OR "vasoconstrictor factors".ti,ab OR "endothelium derived endothelin 1".ti,ab OR "nitric nitrogen".ti,ab OR "endothelium derived nitric oxide".ti,ab OR "vasodilator nitrates".ti,ab OR "vasodilator nitric oxide".ti,ab OR "von willebrand factor cleaving protease".ti,ab OR "adamts13".ti,ab OR "vwf cleaving protease".ti,ab OR "vwf cleaving protease adamts13".ti,ab OR "p2y12 receptors".ti,ab OR "p2y12 receptor".ti,ab OR "p2y12".ti,ab OR "purinergic p2y12 receptors".ti,ab OR "purinergic p2y12 receptor".ti,ab OR "purinergic receptor p2y".ti,ab OR "p2y adp receptor".ti,ab OR "p2y adp receptors".ti,ab OR "p selectin glycoprotein ligand 1".ti,ab OR "p selectin".ti,ab OR CD162.ti,ab OR "platelet p selectin".ti,ab OR "psgl 1 protein".ti,ab OR "selectin P ligand protein".ti,ab OR "endocannabinoid".ti,ab OR "endocannabinoids".ti,ab OR "cb2 receptor".ti,ab OR "cannabinoid receptor cb2".ti,ab OR "cannabinoid receptor 2".ti,ab OR "cannabinoid receptor".ti,ab OR "cannabinoid cb1 receptor".ti,ab OR "cannabinoid cb2 receptor".ti,ab OR "cannabinoid receptor cb1".ti,ab OR "cannabinoid receptor 1".ti,ab OR "cb1 cannabinoid receptor".ti,ab OR "cb2 cannabinoid receptor".ti,ab OR "glyceryl 2 arachidonate".ti,ab OR "2 ag".ti,ab OR "aea".ti,ab OR "2 arachidonoylglycerol".ti,ab OR "anandamide".ti,ab OR "n arachidonoylethanolamine".ti,ab OR "metalloproteinase 9".ti,ab OR "matrix metalloproteinase 9".ti,ab OR "mmp9".ti,ab OR "metalloproteinase".ti,ab OR "metallopeptidases".ti,ab OR "metalloproteinases".ti,ab OR "metallopeptidase".ti,ab OR "type iv collagenase".ti,ab OR "type iv collagenase mmp 2".ti,ab OR "type iv collagenase mmp 9".ti,ab OR "metalloproteinase mmp 9".ti,ab OR "metalloproteinase 1".ti,ab OR "mmp1".ti,ab OR "metalloproteinase mmp1".ti,ab OR "mmp 1".ti,ab OR "mmp 9".ti,ab OR "matrix metalloproteinase 1".ti,ab OR "metalloproteinase 3".ti,ab OR "mmp3".ti,ab OR "matrix metalloproteinase 3".ti,ab OR "metalloproteinase 2".ti,ab OR "mmp2".ti,ab OR "matrix metalloproteinase 2".ti,ab OR "type iv collagenase mmp 2".ti,ab OR "mmp 2 gelatinase a".ti,ab) AND ("female".ti,ab OR "females".ti,ab OR "women".ti,ab OR "woman".ti,ab OR "girl".ti,ab OR "girls".ti,ab)) OR ((exp *"cerebrovascular accident"/ OR "stroke".ti,ab OR "cva".ti,ab OR "cerebrovascular accident".ti,ab OR "cerebrovascular accidents".ti,ab OR "cerebrovascular apoplexy".ti,ab OR "apoplexy".ti,ab OR "brain vascular accidents".ti,ab OR "vascular accidents".ti,ab OR "cerebrovascular stroke".ti,ab OR "cerebrovascular strokes".ti,ab OR "cerebrovascular apoplexy".ti,ab OR "cerebral stroke".ti,ab OR "cerebral strokes".ti,ab OR "acute stroke".ti,ab OR "acute strokes".ti,ab OR "acute cerebrovascular accident".ti,ab OR "acute cerebrovascular accidents".ti,ab OR "cerebrovascular accidents".ti,ab OR "brain infarction".ti,ab OR "brain infarctions".ti,ab OR "brain infarct".ti,ab OR "brain infarcts".ti,ab OR "anterior circulation infarction".ti,ab OR "venous infarction".ti,ab OR "venous infarctions".ti,ab OR "posterior circulation infarction".ti,ab OR "posterior circulation infarcts".ti,ab OR "brain ischemia".ti,ab OR "ischemic encephalopathy".ti,ab OR "ischemic encephalopathies".ti,ab OR "cerebral ischemia".ti,ab OR "cerebral ischemias".ti,ab) AND ("blood coagulation".ti,ab OR "blood coagulation factor".ti,ab OR "blood coagulation factors".ti,ab OR "coagulation factor".ti,ab OR "coagulation factors".ti,ab OR "clotting factor".ti,ab OR "clotting factors".ti,ab OR "blood clotting".ti,ab OR "blood clotting factor".ti,ab OR "blood clotting factors".ti,ab OR "thromboses".ti,ab OR "thrombus".ti,ab OR "blood clot".ti,ab OR "blood clots".ti,ab OR "thrombus formation".ti,ab OR "hemostases".ti,ab OR "platelet activation".ti,ab OR "platelet activations".ti,ab OR "platelet aggregation".ti,ab OR "platelet clotting".ti,ab OR "platelet adhesion".ti,ab OR exp *"Blood Clotting Factor"/ OR "factor i".ti,ab OR "blood coagulation factor i".ti,ab OR "coagulation factor i".ti,ab OR "fibrinogen".ti,ab OR "gamma fibrinogen".ti,ab OR "gamma fibrinogen".ti,ab OR "fi".ti,ab OR "factor ii".ti,ab OR "blood coagulation factor ii".ti,ab OR "differentiation reversal factor".ti,ab OR "coagulation factor ii".ti,ab OR "prothrombin".ti,ab OR "fii".ti,ab OR "thromboplastin".ti,ab OR "coagulin".ti,ab OR "factor iii".ti,ab OR "fiii".ti,ab OR "coagulation factor iii".ti,ab OR "coagulation tissue factor".ti,ab OR "tissue thromboplastin".ti,ab OR "thromboplastin".ti,ab OR "procoagulant".ti,ab OR "tissue factor procoagulant".ti,ab OR "coagulation factor iv".ti,ab OR "factor iv".ti,ab OR "calcium 40".ti,ab OR "calcium".ti,ab OR "fv".ti,ab OR "factor five".ti,ab OR "blood coagulation factor v".ti,ab OR "coagulation factor v".ti,ab OR "coagulation factor v".ti,ab OR "factor v".ti,ab OR "activated factor v".ti,ab OR "coagulation factor va".ti,ab OR "factor va".ti,ab OR "factor v leiden".ti,ab OR "factor five leiden".ti,ab OR "factor seven".ti,ab OR "coagulation factor vii".ti,ab OR "factor vii".ti,ab OR "blood coagulation factor vii".ti,ab OR "proconvertin".ti,ab OR "coagulation factor 7".ti,ab OR "factor 7".ti,ab OR "coagulation factor viia".ti,ab OR "factor viia".ti,ab OR "activated factor vii".ti,ab OR "activated factor vii".ti,ab OR "factor 7a".ti,ab OR "factor 8".ti,ab OR "coagulation factor viii".ti,ab OR "thromboplastinogen".ti,ab OR "blood coagulation factor viii".ti,ab OR "factor eight".ti,ab OR "hyatt c".ti,ab OR "factor viiic".ti,ab OR "f viii c".ti,ab OR "factor viii".ti,ab OR "blood coagulation factor viii".ti,ab OR "coagulation factor viiia".ti,ab OR "factor viiia".ti,ab OR "coagulation factor viii".ti,ab OR "thrombin activated factor viii".ti,ab OR "factor 8a".ti,ab OR "activated factor viii".ti,ab OR "factor nine".ti,ab OR "autoprothrombin ii".ti,ab OR "christmas factor".ti,ab OR "factor ix".ti,ab OR "blood coagulation factor ix".ti,ab OR "factor ix complex".ti,ab OR "factor 9".ti,ab OR "coagulation factor ix".ti,ab OR "coagulation factor ixa".ti,ab OR "factor ixa".ti,ab OR "activated factor ix".ti,ab OR "factor 9a".ti,ab OR "plasma thromboplastin component".ti,ab OR "factor ten".ti,ab OR "autoprothrombin iii".ti,ab OR "coagulation factor x".ti,ab OR "factor x".ti,ab OR "blood coagulation factor x".ti,ab OR "stuart factor".ti,ab OR "stuart prower factor".ti,ab OR "factor 10".ti,ab OR "activated factor x".ti,ab OR "activated coagulation factor x".ti,ab OR "factor xa".ti,ab OR "blood coagulation factor x".ti,ab OR "factor 10a".ti,ab OR "autoprothrombin c".ti,ab OR "factor eleven".ti,ab OR "blood coagulation factor xi".ti,ab OR "plasma thromboplastin".ti,ab OR "plasma thromboplastin antecedent".ti,ab OR "antecedent".ti,ab OR "plasma thromboplastin".ti,ab OR "thromboplastin antecedent".ti,ab OR "coagulation factor xi".ti,ab OR "factor xi".ti,ab OR "coagulation factor 11".ti,ab OR "factor 11".ti,ab OR "activated factor xi".ti,ab OR "coagulation factor xia".ti,ab OR "factor xia".ti,ab OR "blood coagulation factor xi".ti,ab OR "factor 11a".ti,ab OR "factor twelve".ti,ab OR "hageman factor".ti,ab OR "factor 12".ti,ab OR "coagulation factor xii".ti,ab OR "activated factor xii".ti,ab OR "coagulation factor xiia".ti,ab OR "factor xiia".ti,ab OR "blood coagulation factor xii".ti,ab OR "activated prekallikrein".ti,ab OR "prekallikrein".ti,ab OR "factor xii".ti,ab OR "activated hageman factor".ti,ab OR "hageman factor fragment".ti,ab OR "factor thirteen".ti,ab OR "coagulation factor xiii".ti,ab OR "factor xiii".ti,ab OR "factor xiii".ti,ab OR "transamidase".ti,ab OR "fibrinase".ti,ab OR "laki lorand factor".ti,ab OR "blood coagulation factor xiii".ti,ab OR "factor 13".ti,ab OR "fibrin stabilizing factor".ti,ab OR "stabilizing factor".ti,ab OR "activated factor xiii".ti,ab OR "factor 13a".ti,ab OR "plasma transglutaminase".ti,ab OR "transglutaminase".ti,ab OR "beta thromboglobulin".ti,ab OR "beta 2 thromboglobulin".ti,ab OR "thromboglobulin".ti,ab OR "fibrinopeptides a".ti,ab OR "fibrinopeptides b".ti,ab OR "kalliginogenase".ti,ab OR "kallikrein".ti,ab OR "kallikrein kininogenase".ti,ab OR "callicrein".ti,ab OR "kinin forming enzyme".ti,ab OR "kinin forming".ti,ab OR "kallikrein light chain".ti,ab OR "kallikrein padutin".ti,ab OR "kallikrein a".ti,ab OR "beta kallikrein".ti,ab OR "kallikrein b".ti,ab OR "alpha kallikrein".ti,ab OR "plasma kallikrein".ti,ab OR "kininogenin".ti,ab OR "kallikrein i".ti,ab OR "plasma prokallikrein".ti,ab OR "prokallikrein".ti,ab OR "prokinins".ti,ab OR "cystatins".ti,ab OR "t kininogen".ti,ab OR "low molecular weight kininogens".ti,ab OR "lmwk".ti,ab OR "high molecular weight kininogens".ti,ab OR "hmwk".ti,ab OR "fitzgerald factor".ti,ab OR "plasminogen".ti,ab OR "plasminogen activator".ti,ab OR "plasminogen activator inhibitors".ti,ab OR "endothelial plasminogen activator".ti,ab OR "endothelial plasminogen activator inhibitor".ti,ab OR "pai 1".ti,ab OR "serpin e1".ti,ab OR "serpine1".ti,ab OR "serpine1 protein".ti,ab OR "type 1 plasminogen activator inhibitor".ti,ab OR "serpin b2".ti,ab OR "type 2 plasminogen activator inhibitor".ti,ab OR "pai 2".ti,ab OR "pai 3".ti,ab OR "plasminogen activator inhibitor".ti,ab OR "activated protein c inhibitor".ti,ab OR "plasminogen activator inhibitor 3".ti,ab OR "phosphorylcholine".ti,ab OR "acetyl glyceryl phosphorylcholine".ti,ab OR "aggregating factor".ti,ab OR "platelet aggregation enhancing factor".ti,ab OR "agepc".ti,ab OR "thrombocyte aggregating activity".ti,ab OR "alkyl 2 acetyl sn glycerophosphocholine".ti,ab OR "1 alkyl 2 acetyl sn glyceryl 3 phosphorylcholine".ti,ab OR "1 alkyl 2 acetylglycerophosphocholine".ti,ab OR "paf acether".ti,ab OR "platelet activating substance".ti,ab OR "platelet activating substances".ti,ab OR "pf 3".ti,ab OR "platelet factor 3".ti,ab OR "chemokine cxcl4".ti,ab OR "cxcl4".ti,ab OR "chemokine pf4".ti,ab OR "platelet factor 4".ti,ab OR "heparin neutralizing protein".ti,ab OR "antiheparin factor".ti,ab OR "thrombase".ti,ab OR "thrombostat".ti,ab OR "alpha thrombin".ti,ab OR "thrombin jmi".ti,ab OR "jmi".ti,ab OR "thrombin".ti,ab OR "beta thrombin".ti,ab OR "gamma thrombin".ti,ab OR "plasminogen activator".ti,ab OR "tissue activator d 44".ti,ab OR "tisokinase".ti,ab OR "tissue type plasminogen activator".ti,ab OR "ttpa".ti,ab OR "t plasminogen activator".ti,ab OR "tissue type activator".ti,ab OR "rt pa".ti,ab OR "factor viiir ag".ti,ab OR "factor viiir rco".ti,ab OR "ristocetin willebrand factor".ti,ab OR "von willebrand protein".ti,ab OR "von willebrand factor".ti,ab OR "factor viii related antigen".ti,ab OR "ristocetin cofactor".ti,ab OR "plasma factor viii complex".ti,ab OR "vitamin k dependent protein s".ti,ab OR "cofactor protein s".ti,ab OR "protein s".ti,ab OR "protein c".ti,ab OR "antiphospholipid".ti,ab OR "glutamate".ti,ab OR "beta 2 glycoprotein".ti,ab OR "anti beta 2 glycoprotein".ti,ab OR "adp".ti,ab OR "adenosine diphosphate".ti,ab OR "adenosine pyrophosphate".ti,ab OR "adenosine 5' pyrophosphate".ti,ab OR "serotonin".ti,ab OR "5 hydroxytryptamine".ti,ab OR "hippophaine".ti,ab OR "3 2 aminoethyl 2 methyl 1 h indol 5 ol".ti,ab OR "enteramine".ti,ab OR "5 ht".ti,ab OR "hydroxytryptamine".ti,ab OR "thromboxane a2".ti,ab OR "txa2".ti,ab OR "arachidonic acid".ti,ab OR "aa".ti,ab OR "acetylsalicylic acid".ti,ab OR "aspirin".ti,ab OR "platelet derived growth factor".ti,ab OR "pdgf receptor".ti,ab OR "pdgf".ti,ab OR "receptor tgf beta".ti,ab OR "tgfbeta".ti,ab OR "tgf beta".ti,ab OR "platelet transforming growth factor".ti,ab OR "gmp 140".ti,ab OR "p selectin".ti,ab OR "padgem".ti,ab OR "cd62p antigen".ti,ab OR "cd62p antigens".ti,ab OR "cd62p".ti,ab OR "alpha granule membrane protein".ti,ab OR "lecam3".ti,ab OR "gpvi".ti,ab OR "platelet membrane glycoprotein p62".ti,ab OR "glycoprotein gp vi".ti,ab OR "platelet glycoprotein vi".ti,ab OR "human glycoprotein vi".ti,ab OR "human alpha2beta1".ti,ab OR "integrin vla 2".ti,ab OR "vla 2".ti,ab OR "glycoprotein ia iia".ti,ab OR "alpha2beta1integrin".ti,ab OR "very late antigen 2".ti,ab OR "late antigen 2".ti,ab OR "cd49b cd29".ti,ab OR "platelet membrane glycoprotein ia/iia".ti,ab OR "platelet membrane glycoprotein ia iia".ti,ab OR "platelet membrane glycoprotein ia/iia complex".ti,ab OR "platelet membrane glycoprotein ia iia complex".ti,ab OR "platelet glycoprotein gpiib iiia complex".ti,ab OR "glycoproteins iib iiia".ti,ab OR "glycoproteins iib iiia complex".ti,ab OR "integrin alpha iib".ti,ab OR "integrin alpha iib beta 3".ti,ab OR "platelet glycoprotein gpib ix complex".ti,ab OR "platelet glycoprotein gpib ix complex".ti,ab OR "glycoprotein ib ix complex".ti,ab OR "platelet membrane glycoprotein ib".ti,ab OR "glycoprotein ib beta".ti,ab OR "cd42c".ti,ab OR "calcitonin gene related peptide".ti,ab OR "calcitonin gene related peptide ii".ti,ab OR "beta cgrp".ti,ab OR "beta calcitonin gene related peptide".ti,ab OR "alpha cgrp".ti,ab OR "alpha calcitonin gene related peptide".ti,ab OR "calcitonin gene related peptide i".ti,ab OR "cgrp".ti,ab OR "cyclo oxygenase i".ti,ab OR "cox1".ti,ab OR "prostaglandin h synthase 1".ti,ab OR "prostaglandin synthase cyclooxygenase".ti,ab OR "prostaglandin synthase".ti,ab OR "cox 1 synthesis".ti,ab OR "cox 1 prostaglandin".ti,ab OR "cyclooxygenase 1".ti,ab OR "endothelium derived vasoconstrictor factors".ti,ab OR "vasoconstrictor factors".ti,ab OR "endothelium derived endothelin 1".ti,ab OR "nitric nitrogen".ti,ab OR "endothelium derived nitric oxide".ti,ab OR "vasodilator nitrates".ti,ab OR "vasodilator nitric oxide".ti,ab OR "von willebrand factor cleaving protease".ti,ab OR "adamts13".ti,ab OR "vwf cleaving protease".ti,ab OR "vwf cleaving protease adamts13".ti,ab OR "p2y12 receptors".ti,ab OR "p2y12 receptor".ti,ab OR "p2y12".ti,ab OR "purinergic p2y12 receptors".ti,ab OR "purinergic p2y12 receptor".ti,ab OR "purinergic receptor p2y".ti,ab OR "p2y adp receptor".ti,ab OR "p2y adp receptors".ti,ab OR "p selectin glycoprotein ligand 1".ti,ab OR "p selectin".ti,ab OR CD162.ti,ab OR "platelet p selectin".ti,ab OR "psgl 1 protein".ti,ab OR "selectin P ligand protein".ti,ab OR "endocannabinoid".ti,ab OR "endocannabinoids".ti,ab OR "cb2 receptor".ti,ab OR "cannabinoid receptor cb2".ti,ab OR "cannabinoid receptor 2".ti,ab OR "cannabinoid receptor".ti,ab OR "cannabinoid cb1 receptor".ti,ab OR "cannabinoid cb2 receptor".ti,ab OR "cannabinoid receptor cb1".ti,ab OR "cannabinoid receptor 1".ti,ab OR "cb1 cannabinoid receptor".ti,ab OR "cb2 cannabinoid receptor".ti,ab OR "glyceryl 2 arachidonate".ti,ab OR "2 ag".ti,ab OR "aea".ti,ab OR "2 arachidonoylglycerol".ti,ab OR "anandamide".ti,ab OR "n arachidonoylethanolamine".ti,ab OR "metalloproteinase 9".ti,ab OR "matrix metalloproteinase 9".ti,ab OR "mmp9".ti,ab OR "metalloproteinase".ti,ab OR "metallopeptidases".ti,ab OR "metalloproteinases".ti,ab OR "metallopeptidase".ti,ab OR "type iv collagenase".ti,ab OR "type iv collagenase mmp 2".ti,ab OR "type iv collagenase mmp 9".ti,ab OR "metalloproteinase mmp 9".ti,ab OR "metalloproteinase 1".ti,ab OR "mmp1".ti,ab OR "metalloproteinase mmp1".ti,ab OR "mmp 1".ti,ab OR "mmp 9".ti,ab OR "matrix metalloproteinase 1".ti,ab OR "metalloproteinase 3".ti,ab OR "mmp3".ti,ab OR "matrix metalloproteinase 3".ti,ab OR "metalloproteinase 2".ti,ab OR "mmp2".ti,ab OR "matrix metalloproteinase 2".ti,ab OR "type iv collagenase mmp 2".ti,ab OR "mmp 2 gelatinase a".ti,ab) AND ("female".ti,ab OR "females".ti,ab OR "women".ti,ab OR "woman".ti,ab OR "girl".ti,ab OR "girls".ti,ab))) NOT ("Tissue Plasminogen Activator"/ OR "Aspirin") NOT (("Case Report"/ OR "case report".ti OR exp "Review"/ OR "review".ti OR "systematic review"/ OR "systematic review".ti OR exp "meta analysis"/ OR "meta-analysis".ti) NOT ("Clinical Study"/ OR exp "Clinical Trial"/ OR "trial".ti OR "RCT".ti)) NOT (therap*.ti OR treat*.ti) AND (english.la OR dutch.la) NOT ((exp "Child"/ OR exp "Infant"/) NOT (exp "Adult"/))
