# Supplementary material for: Sex Differences in Hemostatic Factors in Patients With Ischemic Stroke and the Relation With Migraine—A Systematic Review
Source: Front Cell Neurosci. 2021 Nov 11;15:711604. doi: 10.3389/fncel.2021.711604 (PMC8632366; doi:10.3389/fncel.2021.711604)
Supplement: Supplementary file 2 [file Table_1.DOCX]

**Supplementary table 1 Study characteristics**

| **Men (N)** | **Women (N)** | **Stroke type (n)** | **age**  **(mean ± SD or median (IQR))** | **women**  **(mean ± SD or median (IQR))** | **men**  **(mean ± SD or median (IQR))** | **Study** |
| --- | --- | --- | --- | --- | --- | --- |
| 141 | 82 | IS |  | 63.6 ± 9.6 | 63.1 ± 8.7 | Lee, 1987 |
| 156 | 149 | TACI (67) PACI/POCI (123) LACI (82) |  | 74 (68-82) | 72 64-79) | Carter, 1997 |
| 131 | 171 | IS (cortical (87), subcortical (96)) |  | 76.9 (75.5-78.3) | 72.2 (70.4-74.0) | Jeppesen, 1998 |
| 72 | 48 | IS | 72 (64-77) |  |  | Mansfield, 1998 |
| 117 | 150 | large vessel occlusion (35) small vessel occlusion (90) cardioembolic (66) undetermined (63) other (13) | 71.0 ± 11.8 |  |  | Tuhrim, 1999 |
| 90 | 50 | IS |  | 67 (43-88) | 65 (44-96) | Kain, 2001 |
| 20 | 19 | non-lacunar stroke MCA (84) cardioembolic (44) | 74 ± 15 |  |  | Montaner, 2001 |
| 40 | 15 | atherothrombotic (16) cardioembolic (9) lacunar (11) undetermined (16) other (3) |  | 56.9 ± 13.0 | 61.5 ± 10.6 | Haapaniemi, 2002 |
| 71 | 30 | IS |  | 62 (43-73) | 60 (38-73) | Kain, 2002 |
| 40 | 15 | atherothrombotic (16) cardioembolic (9) lacunar (11) undetermined (16)  other (3) |  | 56.9 ± 13.0 | 61.5 ± 10.6 | Haapaniemi, 2004 |
| 96 | 68 | Lacunar (87) large artery occlusion (14) undetermined (54) other (4) conflicting etiology (5) | 62.6 ± 10.9* |  |  | Furie, 2004 |
| 212 | 255 | atherothrombotic (77) cardioembolic (86) lacunar (109) undetermined (195) | 68.9 ± 12.7 |  |  | Elkind, 2006 |
| 63 | 72 | IS | 59.2 ± 10.3 |  |  | Saidi, 2007 |
| 114 | 104 | cardioembolic (18) small vessel disease (13) large vessel atherosclerosic (27) undetermined (160) | 57 (23-80) |  |  | Santamaria, 2007 |
| 46 | 34 | cardioembolic (33) atherothrombotic (22) lacunar (21) | 71.0 ± 11.3 |  |  | Skoloudik, 2010 |
| 28 | 15 | IS | 62.4 ± 12.5 |  |  | Blum, 2012 |
| 59 | 46 | IS |  | 67.2 ± 14.8 | 60.2 ± 12.7 | Kisialiou, 2012 |
| 141 | 99 | TACI (28), PACI (89), LACI (46), POCI (58) large vessel occlusion (44) small vessel occlusion (45) cardioembolic (90) undetermined (22) other (20) | 66 (57-77) |  |  | Dong, 2014 |
| 35 | 38 | atherothrombotic (17) cardioembolic (18) lacunar (32) undetermined (6) | 77 (69-85) |  |  | de la Morena-Barrio, 2015 |
| 148 | 94 | TACI (35), PACI (45), LACI (92), POCI (70) large vessel occlusion (62) small vessel occlusion (56) cardioembolic (93) undetermined (16) other (15) | 65.6 ± 12.2 |  |  | Meng, 2015 |
| 15 | 15 | IS | 61.0 ± 7.1 |  |  | Abdelnaseer, 2017 |
| 112 | 113 | IS | 67 (62-73) |  |  | Zhang, 2017 |
| 130 | 116 | IS | 64.2 ± 4.8 |  |  | Li, 2018 |
| 2132 | 1210 | thrombotic (2560) embolic (165) lacunar (698) | 61.8 ± 10.9 |  |  | Zhong, 2019 |

***** calculated weighted mean ± SD
