# Supplementary material for: Sex Differences in Hemostatic Factors in Patients With Ischemic Stroke and the Relation With Migraine—A Systematic Review
Source: Front Cell Neurosci. 2021 Nov 11;15:711604. doi: 10.3389/fncel.2021.711604 (PMC8632366; doi:10.3389/fncel.2021.711604)
Supplement: Supplementary file 3 [file Table_2.DOCX]

**Supplementary table 2 Risk factors**

| **Smoking (%)** | | **Diabetes Mellitus (%)** | | **Hypertension (%)** | | **BMI (kg m^-2^)** | | **Study** |
| --- | --- | --- | --- | --- | --- | --- | --- | --- |
| **Men** | **Women** | **Men** | **Women** | **Men** | **Women** | **Men** | **Women** |  |
| NA | | NA | | NA | | NA | | Lee, 1987 |
| 40 | 29 | NA | | NA | | 24.3 | 24.2 | Carter, 1997 |
| 60.0 | 45.0 | 20.8 | 21.1 | NA | | NA | | Jeppesen, 1998 |
| NA | | 33.3 | | NA | | 25.4 | | Mansfield, 1998 |
| 12.7 | | 36.0 | | 67.8 | | NA | | Tuhrim, 1999 |
| 18.0 | 0.0 | 58.0 | 50.0 | 51.0 | 62.0 | 24.3 | 24.1 | Kain, 2001 |
| NA | | data not shown | | data not shown | | NA | | Montaner, 2001 |
| data not shown | | data not shown | | data not shown | | data not shown | | Haapaniemi, 2002 |
| 18.0 | 0.0 | 58.0 | 50.0 | 51.0 | 62.0 | 24.0 | 24.0 | Kain, 2002 |
| 61.8 | | 12.7 | | 49.1 | | <27 for 47.3% | | Haapaniemi, 2004 |
| 24.4 | | 27.4 | | 68.9 | | NA | | Furie, 2004 |
| 22.7 | | 32.2 | | 67.9 | | NA | | Elkind, 2006 |
| 37.0 | | 26.0 | | 55.3 | | 26.2 | | Saidi, 2007 |
| 45.0 | | 19.0 | | 43.9 | | >30 for 4.4% | | Santamaria, 2007 |
| NA | | 26.2 | | 73.8 | | NA | | Skoloudik, 2010 |
| NA | | NA | | NA | | 26.8 | | Blum, 2012 |
| 22.0 | 10.8 | 37.3 | 41.3 | 69.5 | 44.1 | 25.7 | 25.2 | Kisialiou, 2012 |
| 20.4 | | 28.1 | | 71.5 | | 23.9 | | Dong, 2014 |
| 23.3 | | 49.3 | | 83.6 | | NA | | de la Morena-Barrio, 2015 |
| 21.9 | | 40.0 | | 64.0 | | NA | | Meng, 2015 |
| NA | | 60.0 | | 60.0 | | NA | | Abdelnaseer, 2017 |
| 33.3 | | 38.2 | | 57.3 | | NA | | Zhang, 2017 |
| 52.0 | | 26.0 | | 52.8 | | NA | | Li, 2018 |
| 36.6 | | 17.4 | | 78.6 | | 24.9 | | Zhong, 2019 |
