# Supplementary material for: Sex Differences in Hemostatic Factors in Patients With Ischemic Stroke and the Relation With Migraine—A Systematic Review
Source: Front Cell Neurosci. 2021 Nov 11;15:711604. doi: 10.3389/fncel.2021.711604 (PMC8632366; doi:10.3389/fncel.2021.711604)
Supplement: Supplementary file 4 [file Table_3.DOCX]

**Supplementary table 3 Risk of bias**

| **Representative study population?** | **Selection (non)-exposed from same population?** | **Matching or adjusting?** | **Assessment confounders?** | **Blood sampling different for (non)-exposed?** | **Confidence in assessment of outcome?** | **Confidence outcome of interest not present at start of the study?** | **Confidence in assessment of exposure?** | **Confidence in outcome of interest?** | **Study** |
| --- | --- | --- | --- | --- | --- | --- | --- | --- | --- |
| some concerns | low | high | high | some concerns | some concerns | NA | low | some concerns | Lee, 1987 |
| some concerns | low | some concerns | high | some concerns | some concerns | NA | low | high | Carter, 1997 |
| some concerns | low | low | high | some concerns | some concerns | NA | low | some concerns | Jeppesen, 1998 |
| some concerns | low | some concerns | low | low | low | NA | low | high | Mansfield, 1998 |
| some concerns | low | low | low | some concerns | low | NA | low | high | Tuhrim, 1999 |
| some concerns | low | some concerns | high | low | low | NA | low | high | Kain, 2001 |
| some concerns | low | high | low | low | low | NA | low | low | Montaner, 2001 |
| some concerns | low | high | low | low | low | NA | low | high | Haapaniemi, 2002 |
| some concerns | low | low | low | low | low | NA | low | high | Kain, 2002 |
| some concerns | low | high | low | low | low | NA | low | high | Haapaniemi, 2004 |
| Low | low | high | high | low | low | NA | low | some concerns | Furie, 2004 |
| some concerns | low | low | low | low | low | NA | low | high | Elkind, 2006 |
| some concerns | low | high | low | some concerns | some concerns | NA | low | some concerns | Saidi, 2007 |
| some concerns | low | some concerns | low | low | low | NA | low | high | Santamaria, 2007 |
| some concerns | low | high | high | some concerns | low | NA | low | high | Skoloudik, 2010 |
| some concerns | low | high | high | some concerns | low | NA | low | high | Blum, 2012 |
| some concerns | low | high | low | low | low | NA | low | some concerns | Kisialiou, 2012 |
| High | low | high | high | some concerns | low | NA | low | high | Dong, 2014 |
| some concerns | low | high | high | some concerns | low | NA | low | high | de la Morena-Barrio, 2015 |
| some concerns | low | high | low | low | low | NA | low | low | Meng, 2015 |
| some concerns | low | low | high | low | low | NA | low | low | Abdelnaseer, 2017 |
| some concerns | low | high | low | low | low | NA | low | high | Zhang, 2017 |
| some concerns | low | high | high | high | high | NA | low | high | Li, 2018 |
| low | low | high | low | some concerns | low | NA | low | high | Zhong, 2019 |

Risk of bias was based on the following:
**Representative study population:** low risk (selection of representative population roster such as national association database), some concerns (selection from single centre/city/region), high risk (source population cannot be defined, selection based on self-recruitment).
**Selection (non)-exposed from same population:** low risk (women and men both selected from same data base and same time frame), high risk (women and men from different source population or at different time frame)
**Matching or adjusting?:** low risk (matching or adjustment for most plausible confounders), some concerns (matching or adjustment for some plausible confounders), high risk (matching or adjustment for barely any confounders or no matching or adjustments done)
**Assessment confounders?:** low risk (interview with participants, questionnaires, medical records), high risk (no information on how information was collected, or no data on confounders was collected)
**Blood sampling different for (non)-exposed?:** low risk (no difference between men and women regarding timing of blood sampling, or study regarding genetic data), some concerns (possible difference between men and women due to short time after admission for example), high risk (no information on timing (exception for genetic studies)
**Confidence in assessment of outcome?:** low risk (clear timing of blood sampling), some concerns (large time frame of drawing blood), high risk (timing of blood sampling is unclear)
**Confidence outcome of interest not present at start of the study?:** not applicable for this systematic review.
**Confidence in assessment of exposure?:** exposure is defined as female sex, therefore there is low risk of bias for all studies included.
**Confidence in outcome of interest?:** low risk (blood samples taken before start of treatment), some concerns (provide information on treatment patients received before blood sampling), high risk (blood samples taken after treatment, with no information on treatment, or when it is unknown whether blood samples are taken before or after treatment)
